# Adaptive Machine Learning for Electronic Nose‐Based Forensic VOC Classification

**DOI:** 10.1002/advs.202504657

**Published:** 2025-06-24

**Authors:** Ivan Shtepliuk, Kerstin Montelius, Jens Eriksson, Donatella Puglisi

**Affiliations:** ^1^ Department of Physics Chemistry and Biology Linköping University Linköping 581 83 Sweden; ^2^ Department of Forensic Genetics and Forensic Toxicology National Board of Forensic Medicine Linköping 587 58 Sweden

**Keywords:** artificial olfactory sensor system, electronic nose (e‐nose), machine learning, odor‐based forensic analysis, postmortem interval estimation, volatile organic compounds (VOCs)

## Abstract

Odor detection as forensic evidence is an emerging and transformative approach for identifying individuals in cases where conventional techniques— such as DNA profiling, fingerprints, or gas chromatography‐mass spectroscopy— are insufficient. Key challenges include distinguishing between living and deceased individuals, differentiating human from animal remains, and estimating postmortem intervals. While specially trained dogs remain the gold standard in some contexts, there is growing demand for faster, scalable, and cost‐effective alternatives. Here, a bio‐inspired electronic nose comprising 32 metal‐oxide sensors, integrated with machine learning, is presented as a non‐invasive, real‐time, and reliable tool for volatile organic compound profiling. The system accurately classifies postmortem versus antemortem human biosamples (98.1%), discriminates human from animal tissue (97.2%), and estimates postmortem intervals with high temporal resolution. This study introduces a robust AI‐driven olfactory platform for forensic scent detection, highlighting its potential to complement or replace traditional methods. By coupling volatilome analysis with sensor miniaturization and algorithmic refinement, this approach lays the groundwork for next‐generation forensic diagnostics and odor‐based biomarker discovery.

## Graphical abstract

A 32‐element electronic nose enhanced with machine learning demonstrates exceptional accuracy in differentiating between living and deceased individuals (98.1%), discriminating human and animal specimens (97.2%), and estimating postmortem intervals. This bio‐inspired artificial olfactory sensor system detects unique volatile organic compounds from biological materials, showing its potential to advance scent‐based forensics and other areas where odor detection is critical.

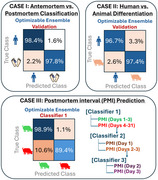

## 1 Introduction

The Nobel Prize in Physics 2024, awarded for discoveries related to artificial intelligence (AI) and machine learning (ML), underscores the pivotal role of these technologies in the advancement of modern science and society.^[^ ^1^ ^]^ Contemporary breakthrough research extensively utilizes these tools, revitalizing established technologies.^[^ ^2^, ^3^, ^4^, ^5^, ^6^, ^7^, ^8^, ^9^, ^10^, ^11^ ^]^ Artificial olfactory sensor systems, particularly electronic noses (*e*‐noses), have emerged as crucial technologies for volatile organic compound (VOC) detection and scent identification.^[^ ^12^, ^13^, ^14^, ^15^ ^]^ The field's foundation was established in the 1980s by Persaud and Dodd's seminal work on gas sensor arrays and artificial neural networks for odor recognition and classification.^[^ ^16^ ^]^ While the 1990s saw increased exploration of ML‐based hardware systems and AI/ML techniques for olfactory systems, progress was hindered by sensor array limitations and restricted access to advanced ML methods.^[^ ^17^, ^18^ ^]^ Early studies primarily employed neural networks and generic algorithms, alongside basic techniques like *k*‐nearest neighbors (kNN), support vector machines (SVM), and discriminant analysis, which were less effective than modern ensemble learning methods such as gently adaptive boosting (*GentleBoost*), introduced in the 2000s.^[^ ^19^ ^]^ Recent advances in ML techniques have significantly enhanced *e*‐nose data analysis capabilities, as investigated herein.

The current ubiquity of ML methodologies, supported by enhanced computational capabilities and big data analytics, facilitates ML‐enhanced *e*‐nose implementation across critical domains. These applications span medicine and life sciences (disease detection, prevention, treatment), food science and agritech (quality assurance, safety, inspection), environmental monitoring (pollution assessment, climate change, sustainability), and forensic sciences (human remains identification, search and rescue, crime prevention).^[^ ^20^, ^21^, ^22^, ^23^, ^24^, ^25^, ^26^, ^27^, ^28^ ^]^ In forensic investigations, *e*‐noses can be used to detect VOCs from various biological matrices including blood, breath, and bodily tissues.^[^ ^29^ ^]^ Each organism possesses a unique volatilome—a distinctive VOC profile influenced by factors like habitat, age, and diet.^[^ ^30^, ^31^ ^]^ This principle underlies gas sensing technologies such as MOS‐based *e*‐noses to distinguish individual body odors.^[^ ^32^, ^33^ ^]^

Notably, metabolic processes associated with specific pathologies (e.g., diabetes, Alzheimer, cancer) or corpse decomposition are common to most humans and mammals, suggesting the generation of VOCs (biomarkers) and changes in the volatilome in a similar manner, allowing to correlate altered odor profiles with disease detection, tissue identification, or postmortem interval (PMI) estimation.^[^ ^31^, ^34^, ^35^, ^36^, ^37^ ^]^ Although gas chromatography‐mass spectrometry (GC‐MS) remains the primary method for VOC identification, it is a time‐consuming and resource‐intensive process.^[^ ^38^, ^39^, ^40^, ^41^ ^]^ Studies have identified approximately 300 characteristic VOCs from human corpses, with variations across different tissues.^[^ ^42^, ^43^ ^]^ To address the challenges of linking VOC concentration changes to decomposition stages, a pattern recognition approach proves more practical than identifying individual VOCs.^[^ ^44^, ^45^ ^]^ By analyzing gas sensor array (*e*‐nose) responses to VOC emissions from various sources (e.g., living/deceased humans, healthy/sick patients), researchers can generate comprehensive datasets to train ML models or so‐called classifiers. This methodology harnesses ML capabilities to identify complex patterns in sensor data, potentially distinguishing subtle VOC profile differences without individual compound identification and quantification.

In this study, we demonstrate the capabilities of a 32‐element *e*‐nose based on metal oxide semiconductor (MOS) sensor technology, augmented by supervised ML algorithms, to address critical forensic challenges. While *e*‐nose technology has been extensively researched, its forensic applications remain underexplored. Current forensic methodologies primarily utilize GC‐MS, DNA profiling, fingerprints, and canine units. In this context, *e*‐nose technology serves as a complementary analytical tool, expanding the range of forensic techniques towards real‐time applicability.

The integration of *e*‐nose with advanced ML methods enables superior handling of high‐dimensional sensor data compared to traditional multivariate approaches like principal component analysis (PCA) or partial least squares‐discriminant analysis (PLS‐DA). Previous studies have successfully applied this combination for PMI estimation using Analysis of Variance (ANOVA) and Multivariate Analysis of Variance (MANOVA), differentiation of living and deceased individuals through fuzzy logic algorithms, and VOC profile analysis using PCA and PLS‐DA.^[^ ^46^, ^50^, ^51^ ^]^ PCA has also been used to estimate time since death for surface‐deposited remains.^[^ ^26^ ^]^ However, existing approaches predominantly rely on dimensionality reduction and clustering algorithms, which are valuable for exploratory data analysis, but may not fully optimize sensor data for specific forensic tasks. Our research addresses this limitation by investigating supervised ML algorithms’ performance with *e*‐nose technology in forensic applications.

## 2 Results and Discussion

We evaluated our *e*‐nose system using a diverse sample collection comprising blood plasma from living individuals, biological materials from deceased individuals (blood, muscle tissue, putrefaction fluids), and animal tissue (pig) both fresh and at various decomposition stages.

This comprehensive sampling enabled assessment of VOC detection and differentiation across various biological materials, species, and decomposition stages, addressing key forensic challenges including living versus deceased discrimination, human versus animal differentiation, and PMI estimation. Given ethical constraints in forensic research, alternative approaches have often been implemented, including the use of synthetic odors (3‐methylindole, putrescine, cadaverine) to simulate the scent of human decomposition and pig tissue as human analogues. However, while pigs present physiological similarities, early‐stage decomposition patterns may differ from humans.^[^ ^27^, ^46^, ^47^, ^48^, ^49^ ^]^ Our methodology incorporated both human biological samples and pig tissue at various decomposition stages to maximize investigative scope within ethical boundaries.

### 2.1 CASE I: Postmortem Versus Antemortem Biosamples

The first case addressed the development of a classifier to differentiate postmortem from antemortem biological samples, analyzing 98 samples from each class. Postmortem samples comprised 34 blood, 29 putrefaction fluids, and 35 muscle tissue samples, while antemortem samples consisted of blood plasma from 98 healthy individuals. A detailed description of the samples can be found in the Experimental Section and Methods. The dataset dimensionality was (98+98) × 32, with each sample generating signals from 32 different sensors, providing sufficient data for robust model training.

**Figure 1:**
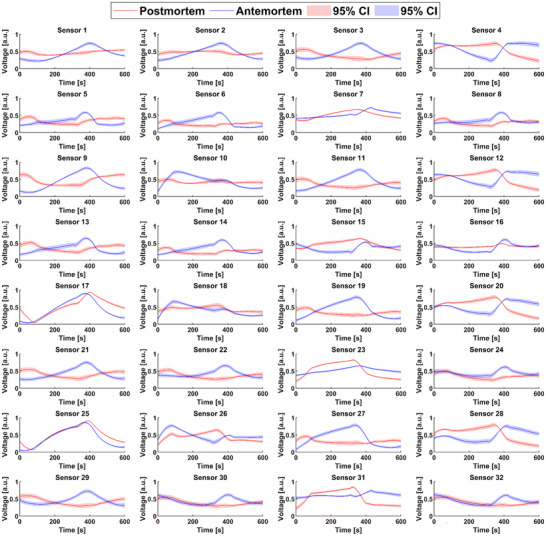
Multi‐sensor response patterns of the *e*‐nose to VOCs from postmortem and antemortem biosamples. The panels show normalized voltage signals with 95% CIs for 32 MOS sensors representing eight sensor types across four temperature‐modulated banks (Table S1, Supporting Information). Red: postmortem; blue: antemortem samples. Distinct response patterns highlight the *e*‐nose's class discrimination capability.

Initial sensor response analysis revealed varying discriminatory capabilities among sensors. **Figure** **1** illustrates the mean responses of all 32 sensors for each class with 95% confidence intervals (CIs). While certain sensors (No. 17 and 25) showed similar signals across classes, others demonstrated consistent offsets. Some sensors (No. 5 and 8) exhibited distinct class‐specific signal patterns with wider CIs, suggesting enhanced sensitivity to sample characteristics. Table S1 (Supporting Information) details sensor models, identification numbers (IDs), and target gases. The *e*‐nose system captured comprehensive VOC profiles through collective response patterns from all 32 sensors, rather than detecting individual molecules. The MOS sensors’ cross‐reactivity to various VOCs in complex mixtures enhanced the detection of class‐specific profiles, with responses reflecting interactions beyond designated target chemicals.

**Figure 2:**
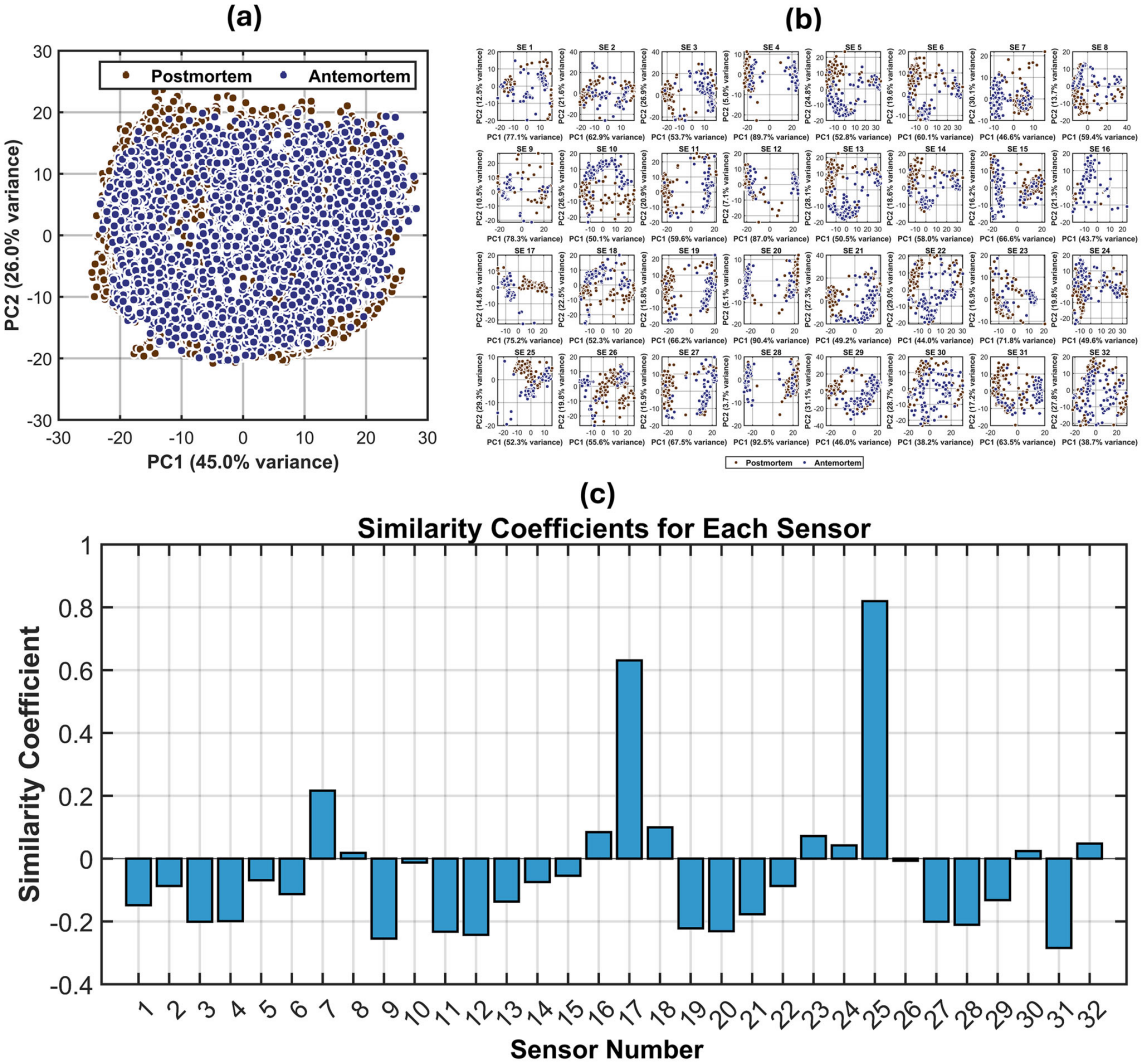
Sensor‐level analysis of *e*‐nose data for discriminating postmortem and antemortem human biosamples. a) PCA plot of the full dataset, showing poor class separation. b) Individual PCA plots for all 32 sensors revealing sensor‐specific discriminatory contributions. c) Bar plot of similarity coefficients calculated as mean of Pearson correlations between all inter‐class signal pairs per sensor; lower values indicate stronger class differentiation.

To evaluate class separation potential using *e*‐nose sensor responses, we employed PCA for data visualization and evaluation of the *e*‐nose's discriminatory power. Initial PCA on the complete dataset (**Figure** **2**a) revealed significant class overlap, indicating that principal components failed to capture class distinctions effectively. Sensor‐specific PCA (Figure 2b) showed partial clustering for sensors No. 10 and 27, suggesting stronger discriminatory power, though most sensors demonstrated substantial overlap, limiting PCA's effectiveness for robust separation. We subsequently extracted 85 features from raw and smoothed‐normalized sensor signals, encompassing statistical, time‐domain, and frequency‐domain characteristics, which enhanced classification accuracy compared to PCA‐based features. To select the most effective ML model, we evaluated 43 classification models in MATLAB's Classification Learner app using the complete feature set. Detailed performance metrics are available in Data S2 (CASEIClassifierSelection.xlsx) (Supporting Information). While 22 models (including SVM, kNN, and neural networks) exceeded 90% validation and test accuracy, the Optimizable Ensemble demonstrated superior performance through automated hyperparameter optimization, including ensemble aggregation methods and learning parameters, making it our optimal choice for binary classification. The final selected classifier was the one that minimized the estimated cross‐validation loss, ensuring an optimal balance between bias and variance.

Given the varying discriminatory abilities of individual sensors, optimizing the sensor array could enhance the Optimizable Ensemble model's classification performance. Previous studies have demonstrated diverse optimization strategies. Dutta et al. utilized inter‐sensor correlations in a 32‐element *e*‐nose (Cyranose 320), while Wijaya et al. employed multiple feature selection methods for an 11‐element *e*‐nose.^[^ ^52^, ^53^ ^]^ Zhang et al. proposed direct sensor significance evaluation using an 8‐element *e*‐nose, and Liu et al. reviewed various optimization approaches.^[^ ^54^, ^55^ ^]^

We implemented a sensor utility evaluation algorithm to compute similarity coefficients for all sensors (Figure 2c). Lower coefficients indicated better differentiation between postmortem and antemortem biosamples. Notably, 22 out of 32 sensors showed negative similarity coefficients, demonstrating distinct responses to each class, while only two sensors (No. 17 and 25) exhibited coefficients above 0.6, suggesting minimal contribution to classification.

We incrementally removed data from sensors with the highest similarity coefficients (i.e., the least useful), creating unique datasets for 90‐10 train‐test splits with a fivefold cross‐validation scheme. We trained and tested the Optimizable Ensemble model at each step. Table S2 and Figure S1 (Supporting Information) detail the included sensors at each step. As shown in Table S3 and Figure S2 (Supporting Information), the exclusion of certain sensors minimally impacted model performance, demonstrating the model's resilience and robustness to reduced sensor input.

**Figure 3:**
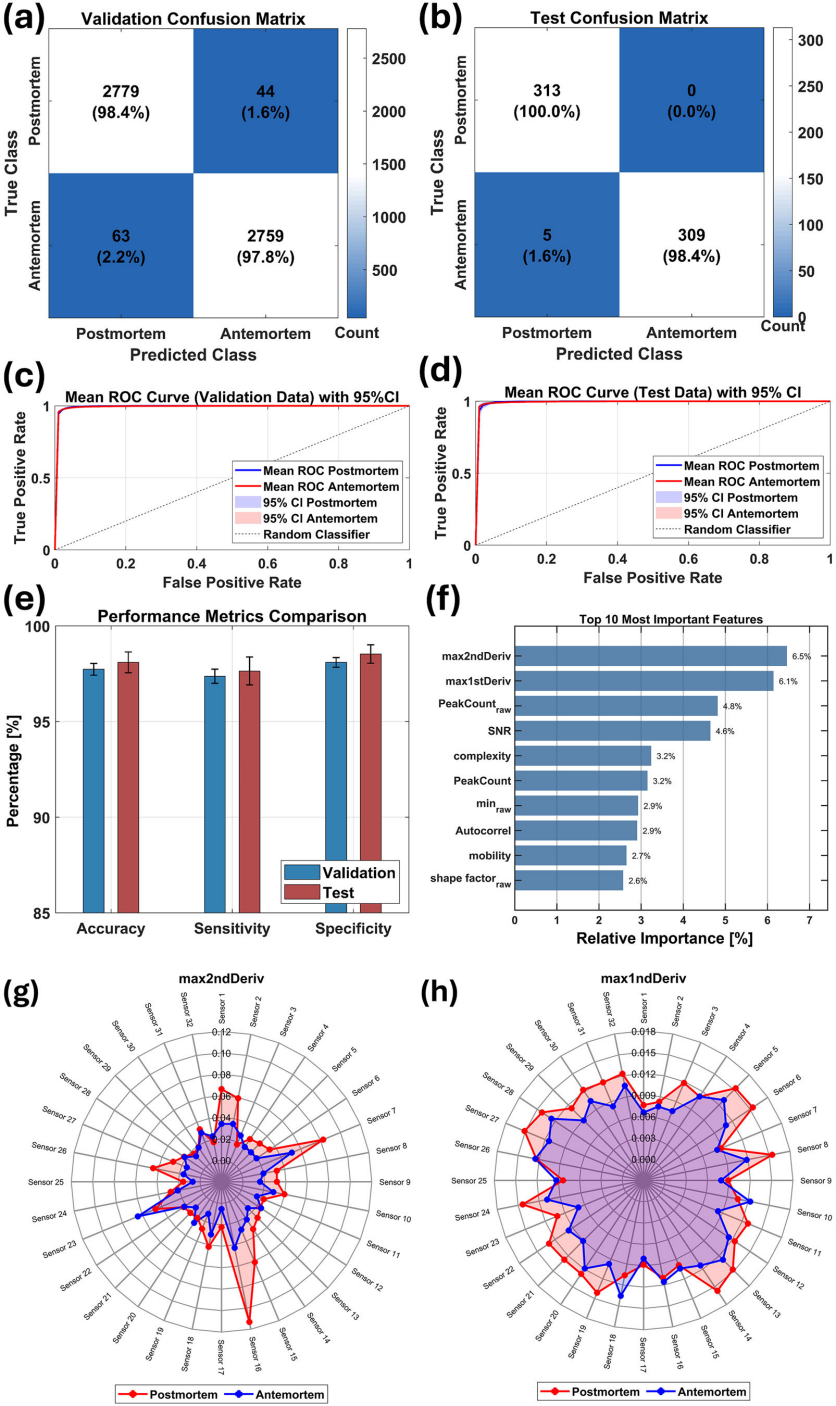
Workflow and performance of the Optimizable Ensemble model for classifying postmortem and antemortem biosamples. a, b) Confusion matrices showing classification results on validation and test datasets. c, d) Mean ROC curves with 95% CIs, demonstrating near‐perfect classification. e) Bar plot of key performance metrics (accuracy, sensitivity, specificity) with 95% CIs across validation and test datasets. f) Feature importance analysis showing the top 10 predictors. g, h) Radar plots of average *max2ndDeriv* and *max1stDeriv* values for all 32 sensors, highlighting class‐level differences between postmortem (red) and antemortem (blue) samples.

For final dataset selection, we retained all sensor data to maximize robustness. This comprehensive approach provides optimal information for model generalization across diverse samples. Multiple sensors better simulate *e*‐nose olfactory capabilities and enhance VOC profile understanding, with different sensors being crucial for distinct forensic classification tasks. **Figure** **3**a,b showcase optimal validation and test confusion matrices from 15 repeated training and testing cycles using the Optimizable Ensemble model. Hyperparameter optimization revealed *GentleBoost* method as the best approach, which strategically balanced model complexity and predictive power through carefully selected parameters: 499 learning cycles, 0.0999 learning rate, 38 maximum decision splits, and minimal leaf size of 1. This ensemble technique sequentially integrates weak learners into a robust classifier, refining errors from previous models and delivering weighted predictions.^[^ ^19^ ^]^

At the validation level, the model demonstrated exceptional performance, correctly classifying 97.8% signals (2759 of 2822) for antemortem and 98.4% (2779 of 2823) for postmortem biosamples. Testing achieved 100% accuracy for postmortem and 98.4% for antemortem samples. Near‐perfect receiver operating characteristic (ROC) curves observed at both the validation and test levels (Figure 3c,d) and narrow CIs (Figure 3e) confirm model stability and reliability.

To validate the reliability of our results, we examined the robustness of the model with more stringent data splitting protocols. Initially, our dataset splitting (90‐10 train‐test) was performed at the observation (or signal) level, allowing sensor signals from the same sample to appear in both training and testing sets. The same applied to *k*‐fold cross‐validation, where different sensor observations of the same sample could be distributed across multiple folds.

This approach offers key advantages: maximizing data utilization for robust training, reflecting real‐world sensor measurement correlations, and maintaining unbiased sensor selection through external utility criteria independent of train‐test split. Our sensor removal algorithm operates independently of sample distribution, using precomputed sensor importance rankings.

To evaluate potential sample‐level leakage effects, we conducted additional analyses using strict sample‐level data splitting, keeping all sensor observations for each sample together. We repeated the sensor removal iteration 32 times, using stratified sampling at the sample level (detailed in CASEI_output.txt and metricsTable_outputCASEI.xlsx, Data S3, Supporting Information). The model maintained high performance across all sensor exclusion stages (Figure S3, Supporting Information), demonstrating resilience to sample‐level data leakage. Sample‐level evaluation using majority voting across sensor‐based predictions achieved near‐perfect classification accuracy, confirming the model robustness.

Regarding sensor‐level data leakage, more complex considerations emerge. While one might consider separating sensor data from the same sample across training and testing sets, the limited sensor count (32) makes this impractical. For instance, with six retained sensors, attempting complete separation might allocate five sensors to training and one to testing. In fivefold cross‐validation, each fold would contain data from just one sensor, creating disjoint feature spaces and compromising model evaluation. Therefore, sensor‐level separation proves neither feasible nor beneficial for sensor ablation experiments. Since sensor exclusion precedes data splitting and relies on independent ranking criteria, the risk of sensor‐level leakage remains minimal. To further validate CASE I results and fully address the issue of potential data leakage, we applied the sensor utility algorithm to training data only in a 90‐10 split, confirming consistent sensor rankings and high model performance on unseen test data (Figures S4–S6, Supporting Information).

To interpret the Optimizable Ensemble model's predictor contributions, we conducted a feature importance analysis using MATLAB's *predictorImportance* function. Among 85 features, only the top 10 predictors significantly influenced classification accuracy (Figure 3f). The maximum value of the second derivative of the signal (*max2ndDeriv*) and the maximum value of first derivative of the signal (*max1stDeriv*) emerged as leading predictors, showing distinctive patterns in the radar plots (Figure 3g,h) that effectively differentiate postmortem and antemortem classes. Notably, individual predictor contributions remained below 7.0%, emphasizing the necessity of incorporating all 85 features for optimal model performance.

**Figure 4:**
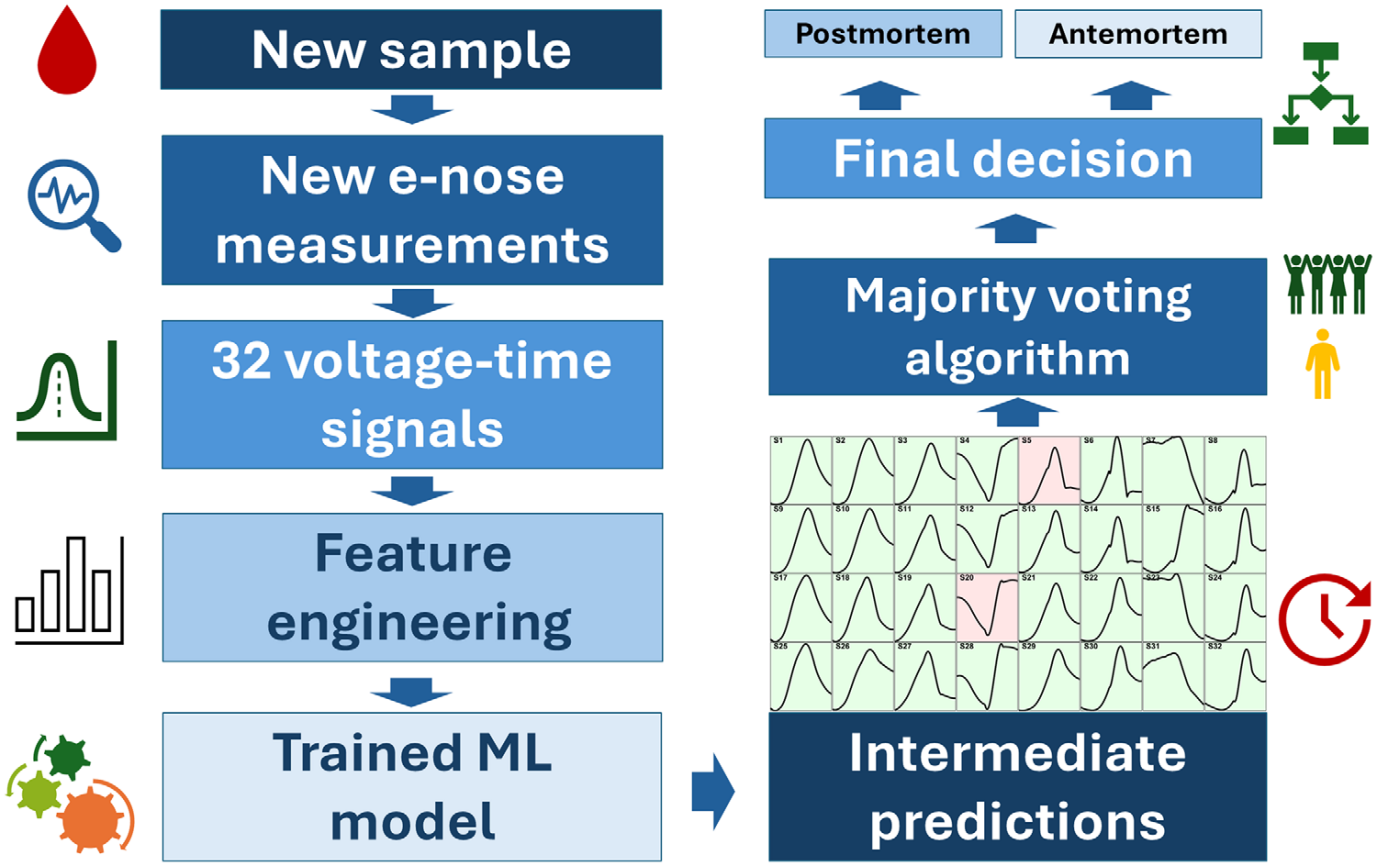
Overview of the classification pipeline from signal acquisition to final output.

Our ML model validation was conducted at the signal level, meaning we classified individual signals belonging to different classes. However, how can we apply this trained and tested model in real‐world scenarios? Let's assume we have samples (whose class is known to us but unknown to the model) that we analyze using a 32‐element sensor array. For each sample, we extract 85 features from 32 signals (32 × 85 external test set) and predict individual signal classification (postmortem or antemortem). We obtain 32 intermediate predictions regarding the sample class. To make a final decision, we develop a majority voting algorithm requiring 90% sensor agreement for class determination, as illustrated in **Figure** **4**.

### 2.2 CASE II: Animal Versus Human Biosamples

**Figure 5:**
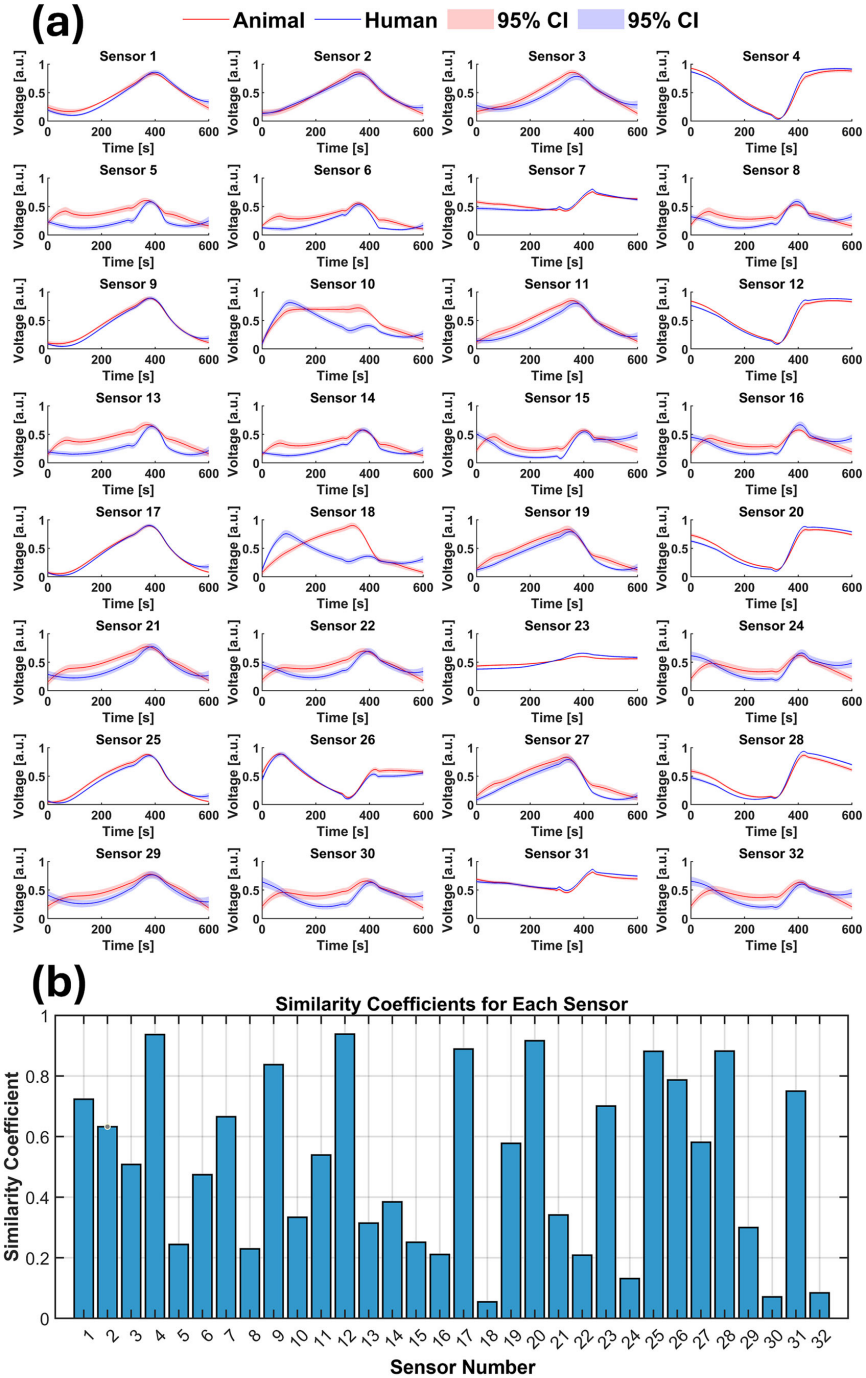
*E*‐nose response patterns and sensor‐level discrimination of animal versus human biosamples. a) Mean sensor responses with 95% CIs across all 32 sensors. Red: animal; blue: human biosamples. b) Bar plot of similarity coefficients (mean Pearson correlations) between paired signals for each sensor. Lower values reflect higher discriminatory power.

The second case addressed the development of a classifier to differentiate between animal and human biosamples, analyzing 70 tissue samples from healthy pigs and 70 blood plasma samples from healthy individuals. The balanced dataset enhanced model reliability and reflected the biological diversity required for robust classification. The use of distinct matrices emitting VOCs was intended to capture species‐specific differences in VOC signatures. Following the methodology established in CASE I, we visualized the average sensor responses with 95% CIs for both classes, enabling a preliminary assessment of each sensor's discriminatory potential (**Figure** **5**a). Sensors No. 4, 12, 17, 20, and 28 exhibited highly similar signal patterns across classes, indicating limited utility for classification. In contrast, sensor No. 18 displayed marked differences in signal shapes, with narrow CIs indicating signal stability and suggesting distinct VOC profiles within each class, resulting in consistent sensor responses.

As in CASE I, PCA analysis failed to achieve adequate separation between classes, underscoring the need for supervised modeling (Figure S7, Supporting Information). Consistent with visual signal assessments, similarity coefficient analysis (Figure 5b) identified sensors No. 18, 30, and 32 as key contributors to class separation, in contrast to the limited utility of sensors No. 4, 12, 17, 20, and 28. Model selection using the full dataset revealed that only the Optimizable Ensemble model exceeded 90% accuracy in both validation and testing (CASEIIClassifierSelection.xlsx, Data S2, Supporting Information). This model was thus selected for further development. Table S4 and Figure S8 (Supporting Information) detail the sensor configurations at each of the 32 training and testing steps. While high performance was observed even without sensor exclusion, accuracy improved substantially from 93.7% at step 1 to 96.7% at step 6 (Table S5 and Figure S9, Supporting Information), primarily through the exclusion of sensors No. 4, 12, 17, 20, and 28, corroborating previous findings on their limited utility in improving classification accuracy. The model at step 6 was selected as final due to its optimal trade‐off among accuracy, sensitivity, and specificity across the train and test datasets, as well as its compact yet informative sensor count to capture complex patterns in the data. Importantly, this model maintained high predictive and generalization power under strict data leakage control measures, including sample‐level stratified data splitting and sequential model evaluation (CASEII_output.txt and metricsTable_outputCASEII.xlsx, Data S2, Supporting Information). To further mitigate data leakage, the sensor utility algorithm was confined to the training set using a 90‐10 split. The consistency in sensor rankings and model performance on completely unseen test data underscores the robustness and real‐world applicability of the final classifier (Figures S10–S12, Supporting Information).

**Figure 6:**
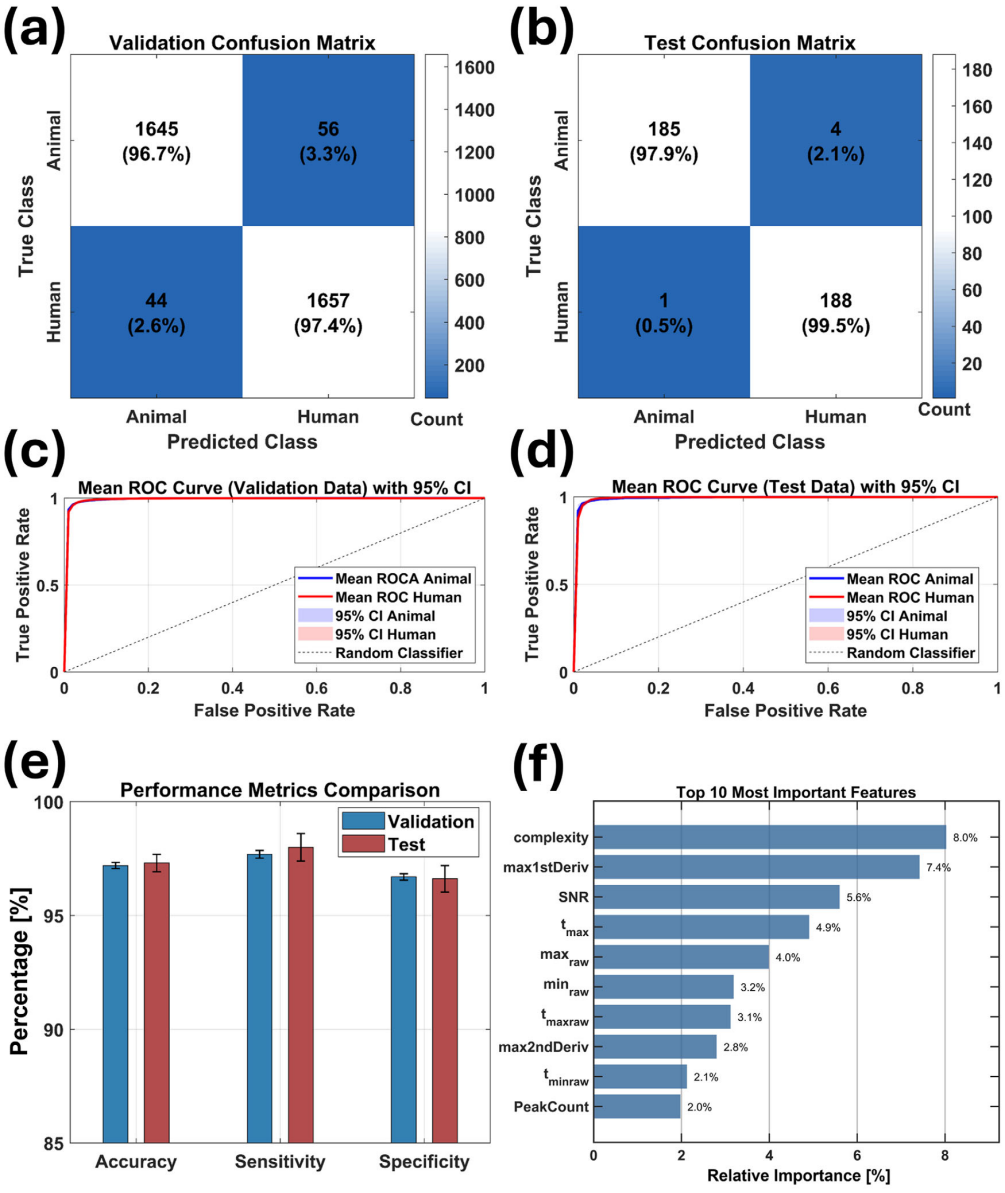
Performance of the Optimizable Ensemble model for animal versus human sample classification. a, b) Confusion matrices for validation and test datasets. c, d) Mean ROC curves with 95% CIs, showing near‐perfect classification. e) Bar plot of model robustness across accuracy, sensitivity, and specificity, with error bars indicating 95% CIs. f) Feature importance analysis of the top 10 input parameters driving model decisions.

Robustness was evaluated over 15 iterations using distinct data splits, demonstrating consistently high model performance and reliability. The optimized hyperparameters—*GentleBoost* boosting method, 490 learning cycles, a moderate learning rate (0.4917), and a small minimum leaf size of 2—reflected a model tuned for rapid adaptation and heightened sensitivity to subtle, class‐specific patterns in the data. Confusion matrices from the best validation and test cycles confirmed strong classification performance, with minimal misclassifications and high sensitivity to true positives and specificity to true negatives (**Figure** **6**a,b). Correspondingly, near‐perfect ROC curves for both phases (Figure 6c,d) further validated the model's resilience to changes in data partitioning. Average accuracy across validation and test phases remained stable at ≈97.0%, with narrow CIs, underscoring consistent model performance on both known and unseen data. Sensitivity and specificity metrics mirrored this stability, showing the model's ability to accurately distinguish between classes (Figure 6e), while tight CIs indicate low variability and reinforce the model's robustness— an essential criterion for deployment in real‐world scenarios involving heterogeneous data.

To identify the most influential features driving class separation, we performed a feature importance analysis. The top 10 predictors (Figure 6f) highlight features such as *complexity*, *max1stDeriv*, and *SNR*, underscoring the significance of signal dynamics in differentiating animal from human biosamples. Radar plots (Figure S13, Supporting Information) illustrate distinct feature profiles across classes, emphasizing their discriminative contribution.

As in CASE I, real‐world sample classification can be operationalized using a majority voting algorithm based on a defined threshold of consistent predictions. Once this threshold is surpassed, a final classification decision can be confidently made.

### 2.3 CASE III: Postmortem Interval Estimation

The third case addressed the temporal profiling of decomposition odors for PMI estimation. A total of 488 measurements were conducted on pig meat samples: 377 within the first three days postmortem (PMI: 1–3 days) and 111 from day 4 to day 32 (PMI: 4–32 days). This imbalance reflects the choice to perform repeated measurements from 50 samples only during the first three days to capture the subtle, time‐sensitive VOC signatures characteristic of initial tissue breakdown. This targeted approach is relevant from a forensic science perspective. Early‐stage VOCs, such as esters, aldehydes, and alcohols, are known to offer high diagnostic value for PMI estimation, while later stages are dominated by strongly odoriferous non‐VOCs, such as putrescine and cadaverine, which may obscure earlier chemical signals.^[^ ^35^, ^56^ ^]^ By repeating our measurements during the first three days and continuing over a month, we were able to test the model's ability to capture the critical VOC signatures of early stages and distinguish them from the stronger, odoriferous compounds that dominate later stages. To explore the model's sensitivity to early‐stage VOCs, we developed Classifier A1 to discriminate between PMI: 1–3 days and PMI: 4–32 days. The Optimizable Ensemble, trained and tested on the entire dataset, consistently outperformed other models across all subcases (Data S2, Supporting Information), although reduced performance in some instances necessitated sensor elimination strategies to enhance accuracy.

**Figure 7:**
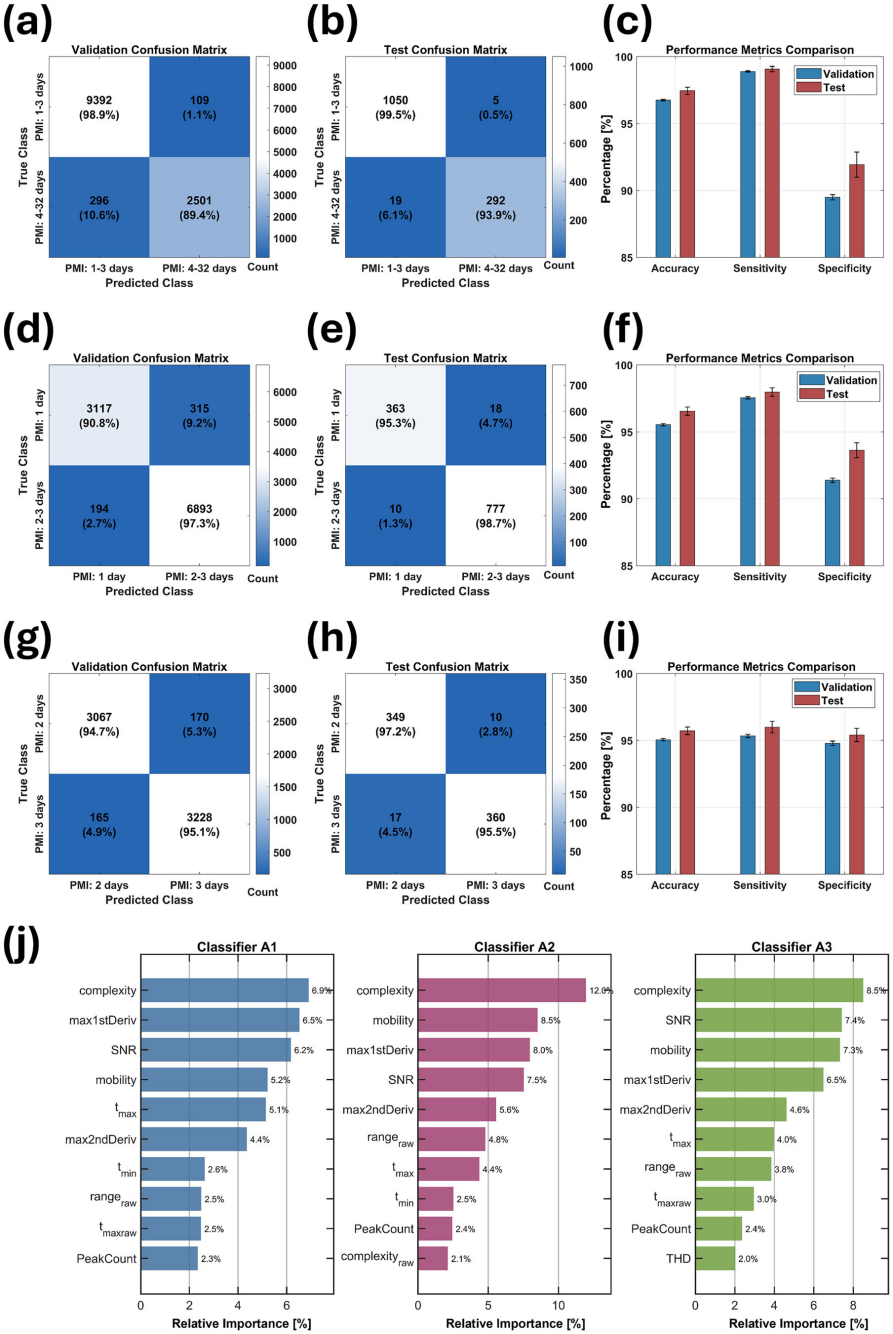
Performance of the Optimizable Ensemble model across different PMI classification tasks. a‐c) Classification between 1–3 days and 4–32 days PMIs (Classifier A1): validation/test confusion matrices and performance metrics with 95% CIs. d‐f) Classification between 1 day and 2–3 days PMIs (Classifier A2). g‐i) Classification between 2 days and 3 days PMIs (Classifier A3). j) Comparative feature importance analysis across Classifier A1, A2, and A3, highlighting key predictors for PMI discrimination.

Given the high dimensionality of the feature matrix used to develop Classifier A1 (85 features × 15616 measurements), we implemented overfitting mitigation techniques, including 5‐fold cross‐validation, 90‐10 train‐test splitting, redundant data removal, and model's robustness evaluation. As shown in **Figure** **7**a,b, Classifier A1 demonstrated strong performance, with a validation error rate of 6.1% (19 misclassifications out of 311) and a training error rate of 10.6% (296 of 2757). Robustness testing confirmed the model's stability across random data partitioning, as evidenced by narrow CIs for key parameters (Figure 7c) and ROC curves with high area under the curve (AUC) values (Figure S14, Supporting Information).

Visual analysis of averaged signals revealed only minor interclass differences (Figure S15, Supporting Information), supported by PCA, which failed to separate the classes either at the *e*‐nose or single‐sensor level (Figure S16a,b, Supporting Information). However, similarity coefficient analysis (Figure S17, Supporting Information) identified sensors No. 24, 30, and 32 as highly discriminative (coefficients < 0.2). An iterative sensor elimination algorithm was applied, resulting in 32 ML models with evolving sensor sets (Table S6 and Figure S18, Supporting Information). In early iterations, the sensitivity exceeded 97.0%, yet the accuracy remained below 90.0%, and the specificity below 62.0%— insufficient for a reliable model (Table S7 and Figure S19, Supporting Information). By step 5, excluding sensors No. 2, 9, 17, and 25 with the highest utility coefficients resulted in significant improvement in accuracy ≈97.0% and specificity above 88.0%. Specificity continued to increase through steps 12–13 peaking at 95.7%, but we selected model at step 5 as final for its optimal trade‐off between model efficiency and feature matrix size. In practice, classification was refined using a majority voting approach, improving diagnostic reliability even with imperfect specificity at the individual prediction level. For instance, an 88.0% individual specificity translated into significantly higher overall specificity when aggregating multiple predictions per sample. Nevertheless, applying sample‐level stratified splitting resulted in universally poor specificity across all 32 steps (CASEIII_CA1_output.txt and metricsTable_outputCASEIII_CA1.xlsx, Data S3, Supporting Information), likely due to dataset imbalance, and highlighting the critical role of data partitioning strategy in model performance.

Encouraged by these results, we refined our approach to enable day‐level PMI resolution within the 1–3‐day range. Classifier A2 (PMI: 1 day versus 2–3 days) and Classifier A3 (PMI: 2 days versus 3 days) achieved accurate differentiation, as illustrated in Figure 7d,e (Classifier A2) and Figure 7g,h (Classifier A3). Robustness testing (Figure 7f,i) and ROC analysis (Figures S20 and S21, Supporting Information) confirmed model stability and low sensitivity to random data partitioning, further supporting their potential for forensic time‐of‐death estimation.

Signal differences in Classifier A2 and A3 were markedly subtler and less distinct than in Classifier A1 (Figures S22 and S23, Supporting Information), as shown by signal overlaps and PCA analyses (Figures S24 and S25, Supporting Information). Despite this, the similarity coefficient algorithms successfully ranked sensors by discriminative value (Figures S26–S29, Table S8 and Figure S15, Supporting Information), allowing iterative sensor elimination to optimize model performance. For Classifier A2 (Table S10 and Figure S30, Supporting Information) high accuracy (>95%), specificity (>97.5%), and sensitivity (>99.0%) where achieved between step 1 and 5, peaking at step 2 after excluding sensor No. 4. Due to its slightly superior performance over step 1, we selected this configuration for final robustness testing. Sample‐level stratified data splitting confirmed the model's stability (CASEIII_CA2_output.txt and metricsTable_outputCASEIII_CA2.xlsx, Data S3, Supporting Information). Classifier A3 (Table S11 and Figure S31, Supporting Information) showed optimal performance between step 4 and 7, with all metrics exceeding 94.0%. We selected step 4 as final (excluding sensors No. 4, 12, and 17). Interestingly, sensor elimination combined with sample‐level stratified data splitting did not further enhance model performance (CASEIII_CA3_output.txt and metricsTable_outputCASEIII_CA3.xlsx, Data S3, Supporting Information), suggesting diminishing returns beyond a certain optimization threshold.

Table S12 (Supporting Information) outlines the optimized hyperparameters for the three classifiers. Classifier A1 and A3 used *GentleBoost*, enabling fine sensitivity through gradual learning rates and small leaf sizes, while Classifier A2 employed *AdaBoostM1* with a higher learning rate and larger leaf size.

Feature importance analysis for Classifier A1, A2, and A3 (Figure 7j) identified *complexity*, *SNR*, *mobility*, and *max1stDeriv* as top contributors across all models, though their rankings varied by classification task. Radar plots of *complexity* for each classifier (Figure S32, Supporting Information) highlight its pivotal role in model performance. This consistency underscores the relevance of these features in differentiating PMI intervals, suggesting that they capture robust signal characteristics applicable to multiple classification tasks. The varying importance scores indicate subtle changes in how each classifier weighs these features, reflecting adjustments based on the specific distinctions required between PMI classes for each case. Additional features, such as *t*_max_, *range*_raw_, and *PeakCoun*t also ranked in the top 10, indicating their usefulness in enhancing classification accuracy for PMI estimation.

**Figure 8:**
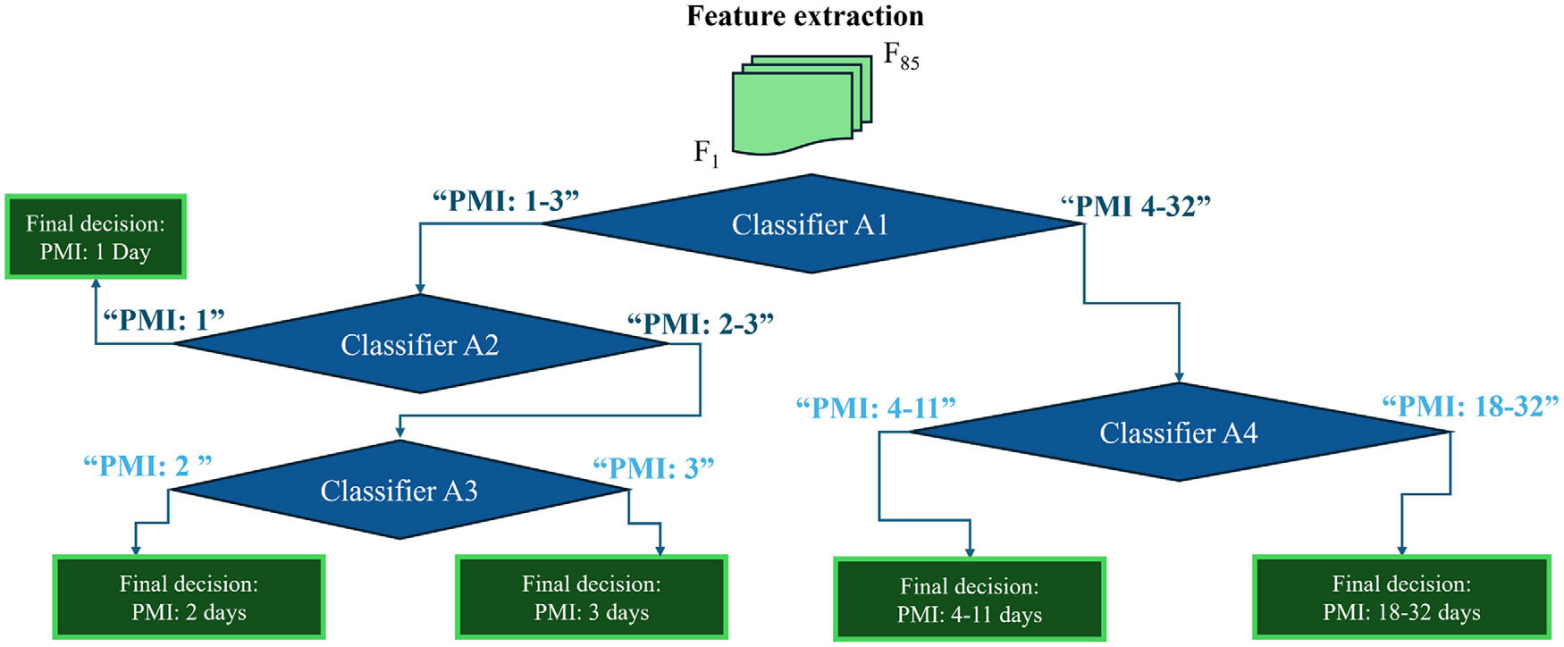
Cascade algorithm that combines Classifier A1, A2, A3, and A4 to estimate PMI.

We further explored classifying late PMI intervals by distinguishing pig meat samples aged 4–11 days versus 18–32 days. Although PCA revealed no clear class separation (Figure S33, Supporting Information), visual analysis of the averaged responses with 95% CI (Figure S34, Supporting Information) and sensor ranking (Figures S35 and S36, and Table S13, Supporting Information) supported the development of Classifier A4. This classifier, alongside Classifier A1, A2, and A3, was integrated into a cascade decision algorithm (**Figure** **8**) for PMI estimation. The system processed unknown samples through sequential classification: A1 (PMI 1–3 versus 4–32 days), A2 (1 day versus 2–3 days), A3 (2 versus 3 days), and A4 (4‐11 versus 18–32 days). Each classifier used pre‐trained models (*.**mat** file) and majority voting to ensure robust, reliable decisions.

Classifier 4 successfully distinguished late PMI ranges, with highest accuracy achieved at step 4 and during steps 13–15 and 22–28 via selective sensor exclusion (Table S14 and Figure S37, Supporting Information #1). However, extensive sensor elimination risks destabilizing model performance by removing valuable signal variability. This highlights the potential drawbacks of using reduced datasets, where important nuances in the data may be sacrificed for the sake of model simplicity or efficiency. Applying a stratified data splitting strategy at the sample‐level did not produce a significant positive response from the model to the sensor elimination procedure (CASEIII_CA4_output.txt and metricsTable_outputCASEIII_CA4.xlsx, Data S3, Supporting Information). To prevent data leakage in CASE III, all sensor utility analyses were confined to training data using a 90‐10 split. Sensor rankings based on similarity coefficients remained consistent across Classifier A1, A2, A3, and A4 (Figures S38–S45, Supporting Information), further validating the robustness of the sensor evaluation strategy.

Future studies will explore whether specific postmortem days in advanced decomposition stages can be predicted accurately using an expanded and appropriately curated training dataset.

## 3 Conclusion 

Our study demonstrates the potential of a MOS‐based *e*‐nose, coupled with advanced ML, for classifying biological samples in forensic contexts based on their VOC profiles. Through a series of classification tasks, we showed that total VOC emissions captured at ambient temperature carry discriminative signal patterns that can be effectively leveraged using pattern recognition algorithms. Rather than a limitation, the cross‐reactivity of MOS sensors to non‐target compounds broadened the VOC detection range, improving classification performance. We introduced a novel sensor ranking method based on discriminative utility, enabling the optimization of sensor combinations tailored to specific classification tasks. To assess model stability, we implemented a robustness evaluation algorithm across 15 randomized data partitions, confirming consistent performance. Observation‐level confusion matrices and ROC curves validated the model's ability to differentiate postmortem from antemortem samples, distinguish between human and animal biosamples, and estimate PMI stages using an animal proxy. Majority voting was applied to finalize classification outcomes, improving overall decision reliability. Furthermore, stratified sample‐level data splitting over 32 iterations ensured resistance to data leakage and demonstrated the robustness of our approach.

Looking ahead, further work will focus on expanding the dataset to include more diverse biological materials, enhancing model generalizability. The system's operational speed— 10 min per sample for measurement and only a few minutes for ML‐based classification— offers a practical, time‐ and cost‐effective alternative to traditional forensic methods.

This research lays the foundation for portable, AI‐driven *e*‐nose systems capable of real‐time VOC‐based analysis, with broad implications for forensics, search‐and‐rescue, disaster management, justice, and public safety. Beyond forensics, this research can contribute to the development of new technologies for environmental monitoring, disease diagnostics, and other areas where odor detection and volatilome analysis are critical.

## 4 Experimental Section

### Postmortem Human Biological Samples

98 autopsy samples including 34 blood, 29 putrefaction fluids, and 35 muscle tissue samples were collected at the National Board of Forensic Medicine, Department of Forensic Medicine in Gothenburg, Sweden, during forensic autopsies of four individuals: three males and one female, aged 27, 41, 69, and 88. These four cases were selected based on differences in biological and environmental factors. They included a fire victim, a drowning victim, a body found outdoors, and another found indoors. Three bodies were heavily decomposed or skeletonized and one body was burned. Samples were prepared in aliquots of 1 *mL* or ≈1.5 *g*, transported under standard conditions in dedicated human remains boxes to the National Board of Forensic Medicine, Department of Forensic Genetics and Forensic Toxicology in Linköping, Sweden, and stored at −20 °C ±1 °C immediately upon arrival. The samples were subsequently transported frozen to the Department of Physics, Chemistry, and Biology, Linköping University, Sweden, and thawed immediately prior to VOC measurements at ambient temperature. All ethical aspects of handling biological materials from deceased humans have been carefully considered and appropriate Ethical Committee approval has been obtained from the Swedish Ethical Review Authority (Etikprövningsmyndigheten) in accordance with ethical review legislation, Act (SFS 2003:460), Reg. No. 2023‐05783‐01.

### Antemortem Human Biological Samples

98 blood plasma samples were collected from 98 healthy living individuals aged 30 to 60 years at Linköping University Hospital, Region Östergötland, Sweden, and transported frozen in boxes filled with synthetic ice to the Department of Physics, Chemistry, and Biology, Linköping University. Samples, prepared in aliquots of 1 *mL* and stored in biobanks in tubes frozen at −80 °C ±1 °C, were kept at −20 °C ±1 °C and thawed immediately prior to VOC measurements at ambient temperature. The inclusion of a relatively broad age range in the antemortem group was intended to account for potential biological variability in signal response, ensuring the model's generalizability and enabling a more comprehensive data analysis. Written informed consent of all participating subjects has been obtained in accordance with the Swedish legislation.

### Postmortem Animal Biological Samples

50 meat samples from 50 six‐month‐old pigs were collected at a slaughterhouse in Gotland, Sweden. The samples, prepared in aliquots of ≈1.5 *g*, were packaged in refrigerated containers (at ≈ +4 °C ±1 °C) and transported to the Department of Physics, Chemistry and Biology, Linköping University, under controlled conditions to ensure preservation of their freshness. Initial measurements of the samples were conducted on the same day they were received, ≈24 h after slaughter. All measurements were performed at ambient temperature. Between measurements, samples were stored under identical refrigeration conditions at a constant temperature of +4 °C ±1 °C to attenuate the degradation process and allow it to occur under controlled environmental conditions. No Ethical Committee approval was required for handling these samples.

### Storage and Handling of Biological Samples

Preserving sample integrity requires careful storage and handling. While ‐80 °C or liquid nitrogen is recommended for long‐term storage (several years), −20 °C remains adequate for shorter durations.^[^ ^57^, ^58^, ^59^, ^60^ ^]^ For short‐term storage (up to 48 h), refrigeration at +4 °C is optimal for the stability of biological materials, with a range of +2 °C to +8 °C considered acceptable. Optimal storage conditions depend on sample type, target analytes, storage duration, and study objectives. Despite the lack of universal guidelines, −20 °C is widely accepted for storing biological samples for up to a few years.^[^ ^61^, ^62^ ^]^ Key considerations include maintaining constant temperature (i.e., minimizing temperature fluctuations), preventing contamination, and avoiding freeze‐thaw cycles.^[^ ^63^, ^64^ ^]^ A previous study on 56 semi‐volatile organic compounds (SVOCs) stored at −20 °C, 4 °C, and 22 °C over 53 days showed high SVOC stability in darkened Amber vials, importance of temperature stability, and collective SVOC disappearance after approximately one month.^[^ ^65^ ^]^ For the untargeted VOC profiling with storage duration of days to weeks, potential collective VOC loss would represent a systematic error, not compromising model robustness. Given adherence to critical handling requirements, −20 °C offers an appropriate balance between practicality and analytical fidelity for the study.

### Biological Variability

This study prioritized inter‐class discrimination over intra‐class variability, aiming to determine whether distinct VOC profiles could be robustly captured by the *e*‐nose system at the class level. The postmortem group comprised samples from individuals of varying age, sex, decomposition stages, and environmental contexts. The antemortem (control) group consisted of blood samples collected from healthy donors, with sample sizes matched across groups. To extend the decomposition‐related variability, animal (pig) meat samples were included as a proxy for non‐human tissue relevant to forensic contexts. While we did not explicitly model intra‐class heterogeneity, signal consistency was assessed by plotting mean signal responses with 95% CIs across all 32 sensors. Narrow CIs indicated strong intra‐class consistency, whereas broader intervals reflected biological diversity or sample heterogeneity. Despite the inherent variability within each group, the classification approach and statistical models consistently captured distinct inter‐class differences, underscoring the system's robustness in differentiating biological sample classes— even in the presence of intra‐class diversity relevant to real‐world forensic scenarios.

### Electronic Nose Setup and Measurement Protocol

An *e*‐nose prototype was used, developed by VOC Diagnostics AB (Linköping, Sweden).^[^ ^66^ ^]^ The system incorporated 32 commercially available metal oxide semiconductor (MOS) gas sensors from the Figaro TGS2X product line, including TGS2602, TGS2603, TGS2620, TGS2611E, TGS2600, TGS2611C, TGS2444, and TGS2610. These sensors were organized into four banks of eight sensors each, with each bank operating at a distinct temperature. Sensor specifications and corresponding IDs are listed in Table S1 (Supporting Information). A photograph of the instrument and more detailed information on the setup and operation of the *e*‐nose can be found elsewhere.^[^ ^67^ ^]^

Biological samples were placed in a dedicated chamber, maintained at ambient temperature (21 °C ± 1 °C) and inserted into the inlet of the *e*‐nose's sensor tunnel for VOC measurement. Each measurement lasted 10 min, with the first 5 min capturing baseline calibration and VOC detection from the sample, followed by sample removal to allow sensor recovery. The system recorded 32 voltage‐time signals at a 0.1 *s* sampling rate, resulting in datasets of size 32 × 6000 per measurement. VOC emissions were measured at ambient temperature without sample heating, enabling the capture of naturally emitted total VOCs. The *e*‐nose was calibrated at the software signal level to ensure stable and repeatable responses, focusing on pattern recognition of total VOC profiles rather than quantification of specific gases. ^[^ ^68^ ^]^ This strategy, optimized for pattern recognition and multivariate analysis, was validated using known reference samples (e.g., postmortem blood, antemortem blood plasma) under controlled laboratory conditions. The system demonstrated robust and reproducible signal behavior when applied to diverse forensic‐relevant biological materials (including blood, muscle tissue, putrefaction fluids, and blood plasma), confirming its utility for classification tasks based on VOC signature patterns.

### Signal Preprocessing and Feature Extraction for Machine Learning

Sensor signals were preprocessed by applying a Savitzky‐Golay filter to smooth the data, followed by range normalization using MATLAB's *normalize* function to scale the features to a fixed range. Raw signals were also retained, as amplitude differences were informative for class discrimination. PCA was applied to identify primary variance sources and potential class separation. A total of 85 features per signal— 42 features from both raw and smoothed‐normalized signals, plus one signal‐to‐noise ratio feature derived from normalized and smoothed‐normalized signals were extracted. These encompassed statistical, time‐domain, and frequency‐domain descriptors derived from the original raw signals, smoothed‐normalized signals, and their derivatives (Tables S15 and S16, Supporting Information). This comprehensive feature extraction ensured a detailed representation of the signals across both time and frequency domains, providing the ML models with a wide range of signal patterns and behaviors to capture. Balanced datasets were constructed for CASE I (98 measurements per class) and CASE II (70 measurements per class), while CASE III (binary classification across clustered PMIs) included variable sample sizes due to frequent repeats in the 1–3 days range.

To quantify each sensor's discriminative capacity, Pearsson correlation coefficients for signals across classes per sensor were computed. Averaged inter‐class correlation values, termed *similarity coefficients*, served as inverse indicators of sensor utility: higher coefficients indicated lower class‐separating power.

Unlike the inter‐sensor correlation method by Dutta et al., which identifies independently acting sensors, the method targets inter‐group discriminability, offering improved performance in class separation tasks. ^[^ ^52^ ^]^ Comparative results between the two methodologies are shown in Figure S46 (Supporting Information). The comparison proves distinct utility profiles, underlining the discriminative capabilities of the method.

Starting from all 32 sensors, we iteratively excluded those with the highest similarity coefficients (the least useful), training and testing ML models at each step. A 90‐10 train‐test split was applied, with 90% of the data used for training and validation and 10% reserved for final testing, along with a 5‐fold cross‐validation scheme. Although removing sensors reduces dimensionality, it was verified that the observation‐to‐feature ratio remained favorable in all cases— CASE I included 6272 observations for 85 features (196 samples × 32 sensors), CASE II 4480 (140 × 32), and CASE III 15616 (488 × 32), mitigating overfitting risk. This was further supported by 5‐fold cross‐validation throughout.

Each sensor‐removal iteration involved: evaluation of model performance (accuracy, sensitivity, specificity, ROC curves); feature importance analysis to assess retained sensor contributions; storage of optimized hyperparameters via MATLAB's Bayesian optimization (30 evaluations per model). This multi‐criteria framework allowed informed sensor elimination while preserving or even enhancing model performance. Final models were selected based on optimal trade‐offs between accuracy, sensitivity, and specificity, and trained on the largest possible dataset for stability analysis.

Model robustness and generalization were ensured via: stratified data splitting, applied at both observation and sample level, to ensure balanced class distributions, reducing bias in training/testing datasets; repeated training/testing of the same dataset (15×), to evaluate model stability and consistency under random splits; model comparison, assessing all possible configurations; fivefold cross‐validation to assess model performance on independent data subsets, minimizing overfitting risk to the training set.

These strategies ensured balanced data representation, model stability, performance consistency across algorithms, and generalization through cross‐validation, mitigating overfitting.

In total, 796 models were trained and tested: 154 for each of CASE I and II (including 43 models for model selection, 32 for random‐split sensor removal, 32 for stratified‐split sensor removal, 32 for training‐driven sensor removal and 15 for robustness), and 488 for CASE III (involving four classifiers). All processing and model development were conducted in MATLAB R2024a.^[^ ^69^ ^]^ An overview of the methodology is presented in Figure S47 (Supporting Information).

### Statistical Analysis

Three primary classification tasks were addressed:

**CASE I**: Discrimination between postmortem and antemortem human biosamples (98 measurements per class);
**CASE II**: Differentiation of animal (pig meat) and human biosamples (70 measurements per class);
**CASE III**: Estimation of PMI using an animal model (488 repeated measurements on 50 pig meat samples across one month).

All statistical analyses were performed in MATLAB. For each classification task, mean responses of the 32 *e*‐nose sensors were calculated and visualized with 95% CIs. Feature importance analysis and PCA were employed to assess data structure and identify key discriminative features. Sensor utility was evaluated by computing the mean Pearson correlation coefficient per sensor, calculated across all inter‐class signal pairs and averaged to obtain a sensor‐level similarity metric. Final models for each classification task were subject to 15‐fold robustness testing, with unique data redistributions between training and test datasets and within five subfolders for validation. Classifier performance metrics— including accuracy, sensitivity, and specificity— were reported as mean values with 95% CIs.

A majority voting algorithm was used to finalize classification outcomes, requiring at least 90% agreement across sensors for sample assignment (e.g., to the postmortem class).

## 5 Current Limitations

The MOS sensors used, while highly sensitive, remain susceptible to environmental influences, sensor drift, and cross‐reactivity. Based on our experience, polar and non‐polar VOCs interact differently with sensor materials, which may result in limited sensor's ability to detect non‐polar VOCs with zero dipole moment.^[^ ^70^ ^]^ These challenges can compromise stability and complicate calibration, especially in real‐world forensic environments. The ML models— although trained and tested using stratified sampling, cross‐validation, and robustness testing— were developed on a limited dataset. As such, their generalizability to more diverse forensic scenarios, including different causes of death, decomposition conditions, and sample types, remains uncertain. Furthermore, reproducibility across different devices or laboratory settings may be affected by hardware‐specific characteristics and the context‐sensitive nature of VOC emissions, underscoring the need for system standardization and cross‐platform compatibility.

Overcoming current limitations through focused technical enhancements and comprehensive validation studies will be critical to unlocking the full potential of *e*‐nose technology in forensic science and related applications.

## 6 Challenges and Future Work

Looking forward, several technical and methodological developments are essential to advance this field. Expanding the diversity of postmortem samples, especially under varied environmental and temporal conditions, will improve our understanding of decomposition‐related VOC patterns and allow for more nuanced time‐of‐death predictions. Optimizing the performance of MOS sensors, through the development of novel sensing materials to reduce drift and improve selectivity, along with the exploration of other sensor technologies, will be critical to maintaining signal quality in real‐world applications. Moreover, validating our models with contaminated or mixed samples under operational forensic conditions is a priority, especially when evaluating robustness against environmental interference.

To facilitate real‐world deployment, future work will also focus on sensor reduction and *e*‐nose miniaturization, ensuring classification accuracy is preserved through targeted feature selection and optimized model tuning. The establishment of standardized preprocessing protocols, coupled with integration into smart devices, will promote cross‐platform compatibility and practical field implementation. Ultimately, interdisciplinary collaboration among forensic experts, scientists, engineers, and regulatory bodies will be essential for realizing a reliable, reproducible, and scalable framework for forensic VOC profiling using low‐cost sensors and advanced analytical methods.

## Conflict of Interest

The authors declare no conflict of interest.

## Supporting information



Supporting Information

Supporting Information

Supporting Information

## Data Availability

The data that support the findings of this study are available from the corresponding author upon reasonable request.
