# Supplementary material for: Adaptive Machine Learning for Electronic Nose‐Based Forensic VOC Classification
Source: Adv Sci (Weinh). 2025 Jun 24;12(36):e04657. doi: 10.1002/advs.202504657 (PMC12463043; doi:10.1002/advs.202504657)
Supplement: Supplementary file 1 — Supporting Information [file ADVS-12-e04657-s001.docx]

Supporting Information

Adaptive Machine Learning for Electronic Nose-Based Forensic VOC Classification

*Ivan Shtepliuk^1^, Kerstin Montelius^2^, Jens Eriksson^1^ and Donatella Puglisi^1*^*

^1^Department of Physics, Chemistry and Biology, Linköping University, 581 83, Linköping, Sweden

^2^Department of Forensic Genetics and Forensic Toxicology, National Board of Forensic Medicine, 587 58 Linköping, Sweden

E-mail: donatella.puglisi@liu.se

**Table S1**. Summary of MOS sensors and target gases.

| **Sensor ID** | **Sensor No.** | **Model** | **Target Gases** |
| --- | --- | --- | --- |
| *S1* | *1, 9, 17, 25* | *TGS2602* | *VOCs (Toluene), Ethanol, Ammonia, Hydrogen Sulfide* |
| *S2* | *2, 10, 18, 26* | *TGS2603* | *Ethanol, Hydrogen Sulfide, Hydrogen, Amine-series and sulfurous odor gases: Trimethylamine, Methyl Mercaptan* |
| *S3* | *3, 11, 19, 27* | *TGS2620* | *Methane, Carbon Monoxide, Isobutane, Ethanol, Hydrogen* |
| *S4* | *4, 12, 20, 28* | *TGS2611-E00* | *Methane* |
| *S5* | *5, 13, 21, 29* | *TGS2600* | *Hydrogen, Carbon Monoxide, Isobutane, Ethanol, Hydrogen* |
| *S6* | *6, 14, 22, 30* | *TGS2611-C00* | *Methane, Isobutane, Hydrogen, Ethanol* |
| *S7* | *7, 15, 23, 31* | *TGS2444* | *Ammonia* |
| *S8* | *8, 16, 24, 32* | *TGS2610* | *Isobutane, Propane, Ethanol, Hydrogen, Methane* |

**Table S2**. Sensors retained at each iteration of the sensor-elimination procedure for postmortem vs. antemortem classification (CASE I).

| **Step** | **No. of Sensors** | **Remaining Sensors at Each Step of Iterative Removal** |
| --- | --- | --- |
| 1 | 32 | 1 2 3 4 5 6 7 8 9 10 11 12 13 14 15 16 17 18 19 20 21 22 23 24 25 26 27 28 29 30 31 32 |
| 2 | 31 | 1 2 3 4 5 6 7 8 9 10 11 12 13 14 15 16 17 18 19 20 21 22 23 24 26 27 28 29 30 31 32 |
| 3 | 30 | 1 2 3 4 5 6 7 8 9 10 11 12 13 14 15 16 18 19 20 21 22 23 24 26 27 28 29 30 31 32 |
| 4 | 29 | 1 2 3 4 5 6 8 9 10 11 12 13 14 15 16 18 19 20 21 22 23 24 26 27 28 29 30 31 32 |
| 5 | 28 | 1 2 3 4 5 6 8 9 10 11 12 13 14 15 16 19 20 21 22 23 24 26 27 28 29 30 31 32 |
| 6 | 27 | 1 2 3 4 5 6 8 9 10 11 12 13 14 15 19 20 21 22 23 24 26 27 28 29 30 31 32 |
| 7 | 26 | 1 2 3 4 5 6 8 9 10 11 12 13 14 15 19 20 21 22 24 26 27 28 29 30 31 32 |
| 8 | 25 | 1 2 3 4 5 6 8 9 10 11 12 13 14 15 19 20 21 22 24 26 27 28 29 30 31 |
| 9 | 24 | 1 2 3 4 5 6 8 9 10 11 12 13 14 15 19 20 21 22 26 27 28 29 30 31 |
| 10 | 23 | 1 2 3 4 5 6 8 9 10 11 12 13 14 15 19 20 21 22 26 27 28 29 31 |
| 11 | 22 | 1 2 3 4 5 6 9 10 11 12 13 14 15 19 20 21 22 26 27 28 29 31 |
| 12 | 21 | 1 2 3 4 5 6 9 10 11 12 13 14 15 19 20 21 22 27 28 29 31 |
| 13 | 20 | 1 2 3 4 5 6 9 11 12 13 14 15 19 20 21 22 27 28 29 31 |
| 14 | 19 | 1 2 3 4 5 6 9 11 12 13 14 19 20 21 22 27 28 29 31 |
| 15 | 18 | 1 2 3 4 6 9 11 12 13 14 19 20 21 22 27 28 29 31 |
| 16 | 17 | 1 2 3 4 6 9 11 12 13 19 20 21 22 27 28 29 31 |
| 17 | 16 | 1 3 4 6 9 11 12 13 19 20 21 22 27 28 29 31 |
| 18 | 15 | 1 3 4 6 9 11 12 13 19 20 21 27 28 29 31 |
| 19 | 14 | 1 3 4 9 11 12 13 19 20 21 27 28 29 31 |
| 20 | 13 | 1 3 4 9 11 12 13 19 20 21 27 28 31 |
| 21 | 12 | 1 3 4 9 11 12 19 20 21 27 28 31 |
| 22 | 11 | 3 4 9 11 12 19 20 21 27 28 31 |
| 23 | 10 | 3 4 9 11 12 19 20 27 28 31 |
| 24 | 9 | 3 9 11 12 19 20 27 28 31 |
| 25 | 8 | 3 9 11 12 19 20 28 31 |
| 26 | 7 | 9 11 12 19 20 28 31 |
| 27 | 6 | 9 11 12 19 20 31 |
| 28 | 5 | 9 11 12 20 31 |
| 29 | 4 | 9 11 12 31 |
| 30 | 3 | 9 12 31 |
| 31 | 2 | 9 31 |
| 32 | 1 | 31 |


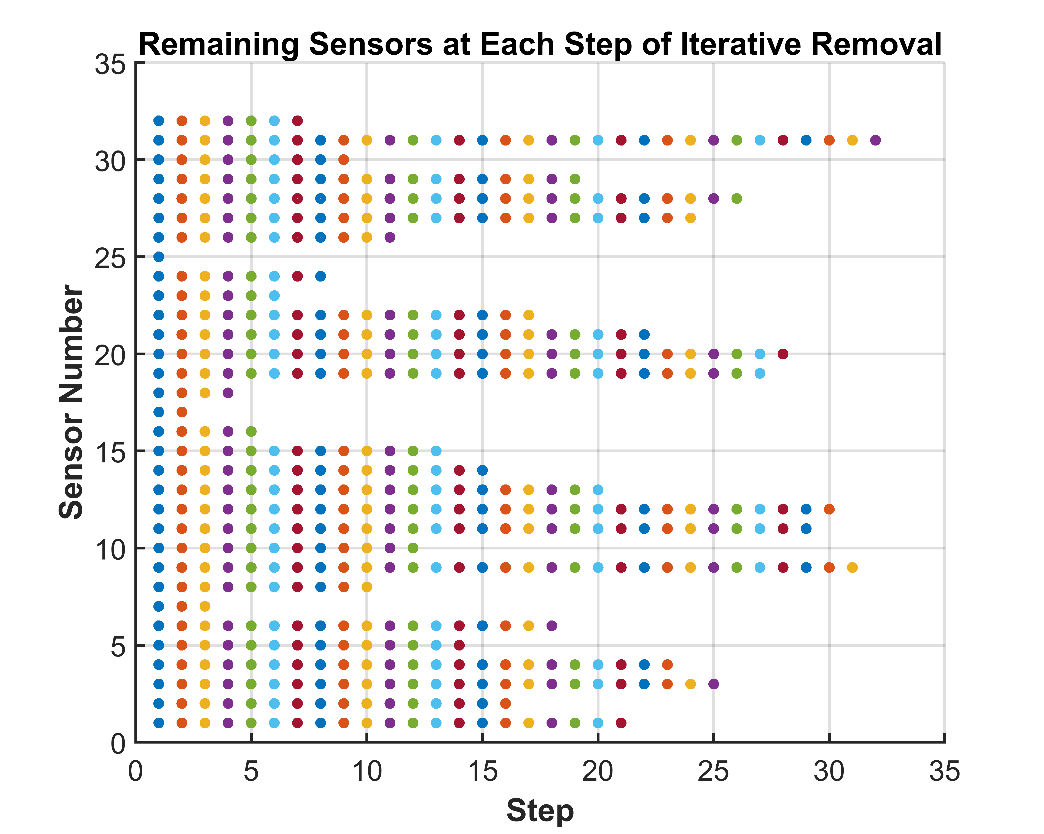


**Figure S1.** Graphical representation of **Table S2**.

**Table S3**. Evolution of validation and test performance metrics over 32 steps of the iterative sensor-elimination procedure for postmortem vs. antemortem classification (CASE I). Bold red: step selected.

| **Step** | **Train Accuracy** | **Train Sensitivity** | **Train Specificity** | **Test Accuracy** | **Test Sensitivity** | **Test Specificity** |
| --- | --- | --- | --- | --- | --- | --- |
| **1** | **0.97679** | **0.98123** | **0.97236** | **0.98724** | **0.99042** | **0.98408** |
| 2 | 0.97952 | 0.98245 | 0.97659 | 0.98023 | 0.98350 | 0.97697 |
| 3 | 0.96183 | 0.96674 | 0.95692 | 0.97449 | 0.97619 | 0.97279 |
| 4 | 0.97830 | 0.98202 | 0.97459 | 0.98944 | 0.98944 | 0.98944 |
| 5 | 0.98117 | 0.98219 | 0.98016 | 0.98905 | 0.99635 | 0.98175 |
| 6 | 0.96179 | 0.96094 | 0.96264 | 0.96975 | 0.98868 | 0.95076 |
| 7 | 0.97842 | 0.97819 | 0.97864 | 0.97446 | 0.96471 | 0.98425 |
| 8 | 0.97778 | 0.97778 | 0.97778 | 0.97755 | 0.97551 | 0.97959 |
| 9 | 0.97733 | 0.97685 | 0.97780 | 0.98511 | 0.99149 | 0.97872 |
| 10 | 0.97758 | 0.97979 | 0.97536 | 0.98222 | 0.99556 | 0.96889 |
| 11 | 0.95877 | 0.96033 | 0.95722 | 0.96056 | 0.96279 | 0.95833 |
| 12 | 0.96032 | 0.95896 | 0.96168 | 0.97567 | 0.97087 | 0.98049 |
| 13 | 0.96259 | 0.96088 | 0.96429 | 0.96173 | 0.96939 | 0.95408 |
| 14 | 0.96480 | 0.95823 | 0.97136 | 0.97581 | 0.97849 | 0.97312 |
| 15 | 0.96599 | 0.96159 | 0.97040 | 0.96591 | 0.94318 | 0.98864 |
| 16 | 0.96566 | 0.96133 | 0.96998 | 0.96997 | 0.95783 | 0.98204 |
| 17 | 0.96599 | 0.96315 | 0.96884 | 0.97125 | 0.96178 | 0.98077 |
| 18 | 0.98148 | 0.97959 | 0.98337 | 0.97959 | 0.97959 | 0.97959 |
| 19 | 0.96923 | 0.97409 | 0.96437 | 0.97810 | 0.99270 | 0.96350 |
| 20 | 0.98605 | 0.98779 | 0.98431 | 0.98031 | 0.96850 | 0.99213 |
| 21 | 0.96788 | 0.96975 | 0.96601 | 0.95319 | 0.96610 | 0.94017 |
| 22 | 0.96857 | 0.96704 | 0.97010 | 0.95349 | 0.96262 | 0.94444 |
| 23 | 0.95805 | 0.95692 | 0.95918 | 0.97959 | 0.96939 | 0.98980 |
| 24 | 0.98174 | 0.98615 | 0.97733 | 0.98295 | 0.97727 | 0.98864 |
| 25 | 0.95609 | 0.95609 | 0.95609 | 0.98077 | 0.98718 | 0.97436 |
| 26 | 0.95061 | 0.94660 | 0.95462 | 0.95620 | 0.98529 | 0.92754 |
| 27 | 0.96506 | 0.97164 | 0.95849 | 0.98291 | 1.00000 | 0.96552 |
| 28 | 0.96485 | 0.96825 | 0.96145 | 0.94898 | 0.95918 | 0.93878 |
| 29 | 0.96034 | 0.97450 | 0.94618 | 0.97436 | 1.00000 | 0.94872 |
| 30 | 0.97358 | 0.98868 | 0.95849 | 1.00000 | 1.00000 | 1.00000 |
| 31 | 0.98017 | 0.98295 | 0.97740 | 0.94872 | 0.95000 | 0.94737 |
| 32 | 0.99435 | 1.00000 | 0.98864 | 1.00000 | 1.00000 | 1.00000 |

**Figure S2**. Graphical representation of **Table S3**.

**

**Figure S3**. Model performance metrics (accuracy, sensitivity, specificity) plotted against the number of sensors retained for CASE I.


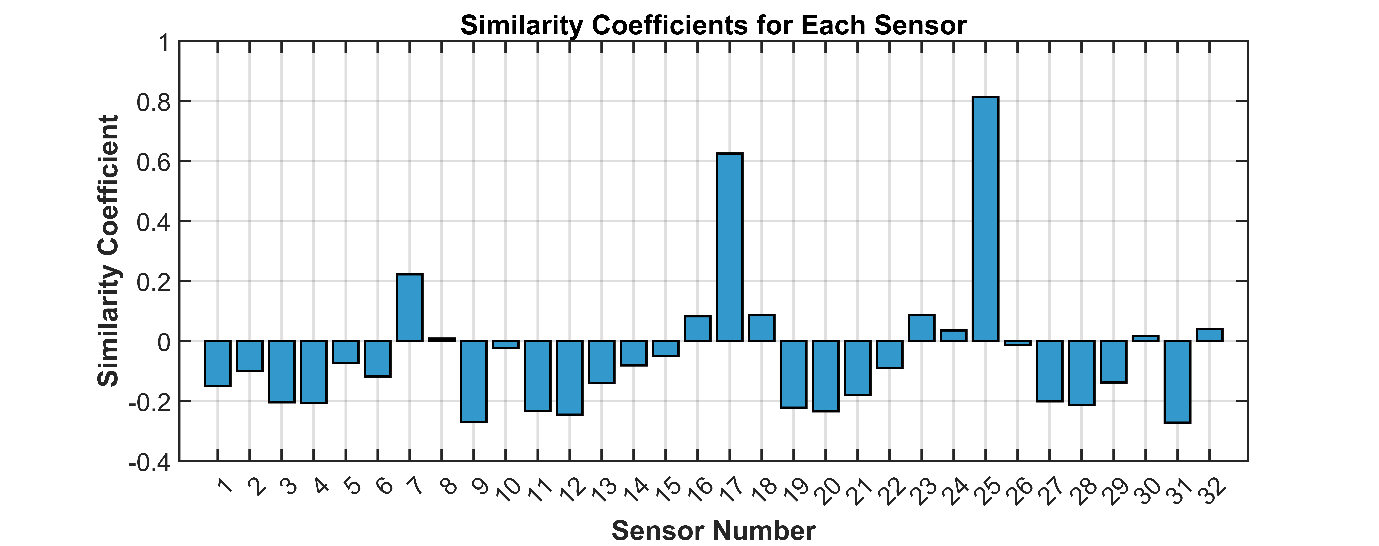


**Figure S4.** Similarity coefficients for each of the 32 sensors in discriminating deceased vs. living individuals (CASE I), calculated using the training dataset. Bars denote mean Pearson correlations across all inter-class signal pairs per sensor.


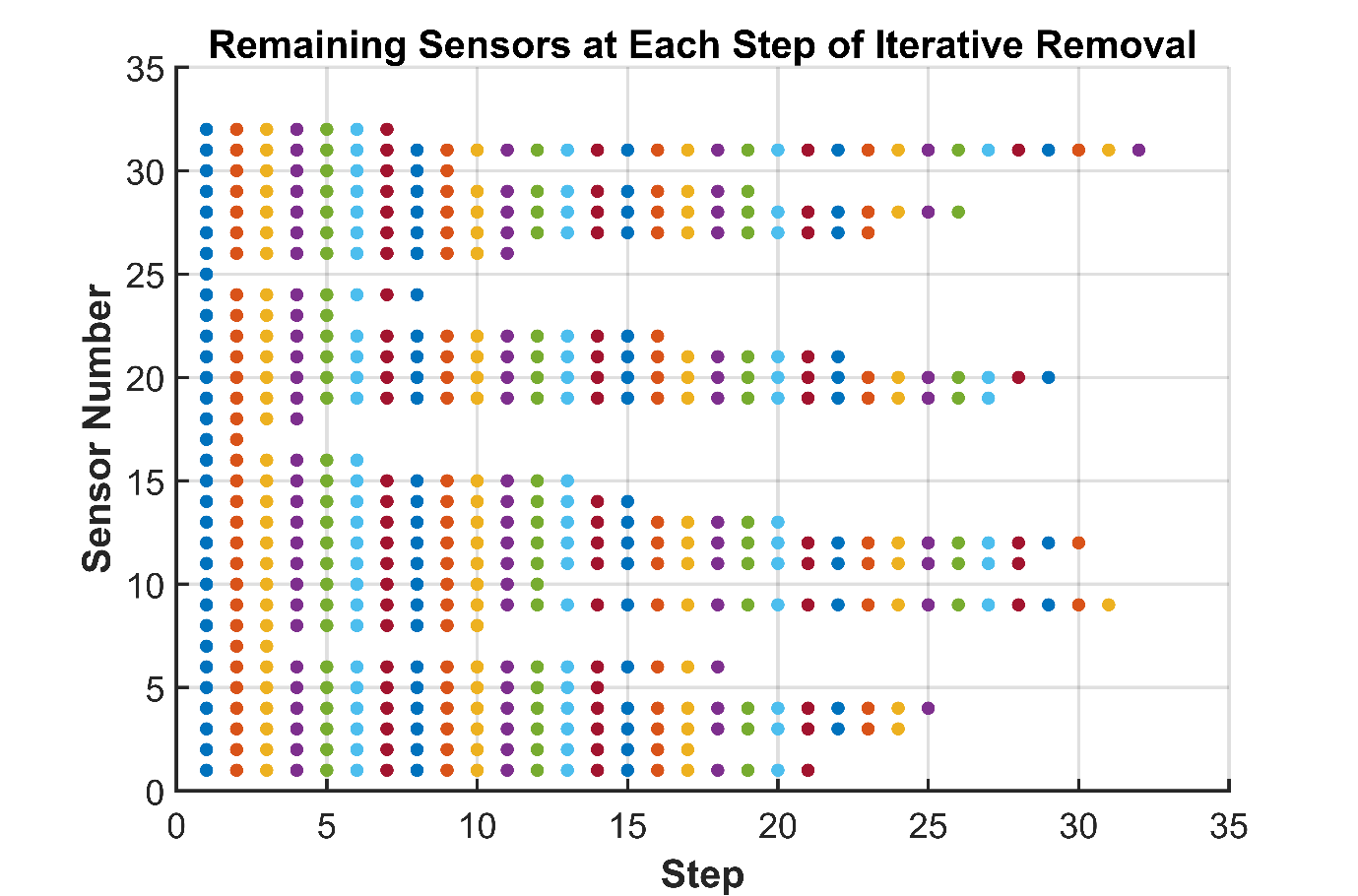


**Figure S5**. Graphical representation of sensor retention across removal steps (applied to training dataset) for postmortem vs. antemortem discrimination (CASE I).


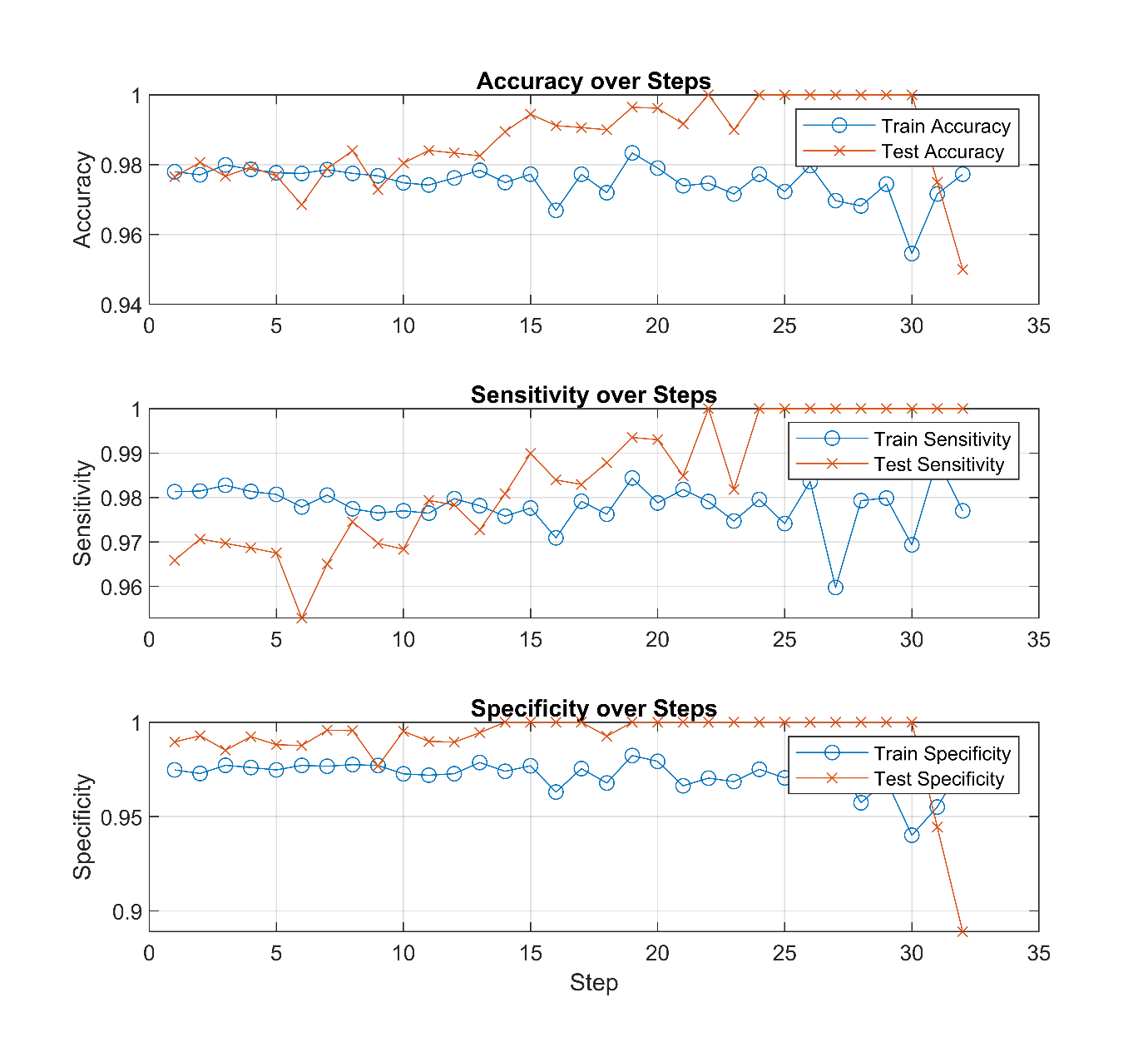


**Figure S6**. Model performance metrics (accuracy, sensitivity, specificity) plotted against the number of sensors retained for CASE I, with the sensor utility ranking algorithm applied to the training dataset only.


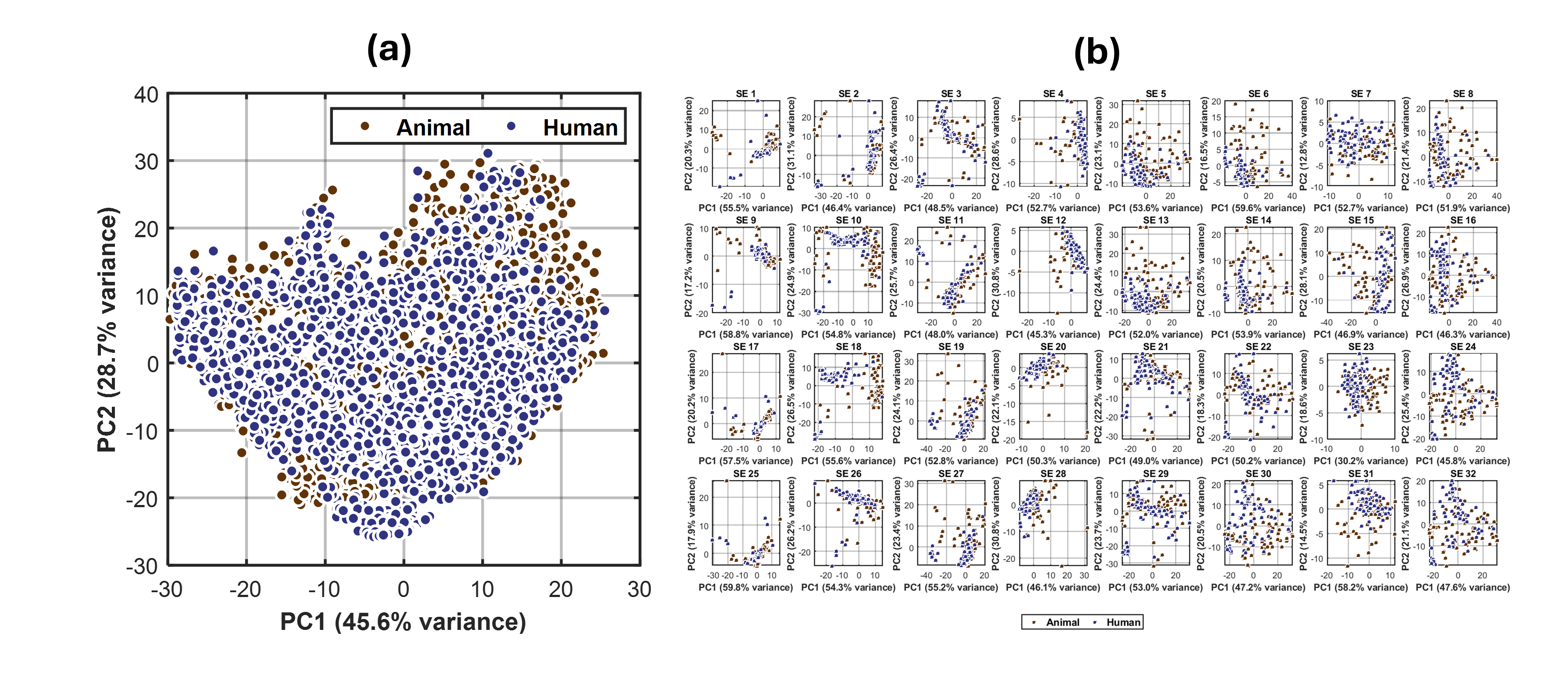


**Figure S7**. Principal component analysis (PCA) for animal vs. human classification (CASE II). a) PCA plot of the full e-nose dataset, showing class overlap. b) Sensor-level PCA plots for each of the 32 sensors, illustrating individual contribution to class separation.

**Table S4**. Sensors retained at each iteration of the sensor-elimination procedure for animal vs. human classification (CASE II).

| **Step** | **No. of Sensors** | **Remaining Sensors at Each Step of Iterative Removal** |
| --- | --- | --- |
| 1 | 32 | 1 2 3 4 5 6 7 8 9 10 11 12 13 14 15 16 17 18 19 20 21 22 23 24 25 26 27 28 29 30 31 32 |
| 2 | 31 | 1 2 3 4 5 6 7 8 9 10 11 13 14 15 16 17 18 19 20 21 22 23 24 25 26 27 28 29 30 31 32 |
| 3 | 30 | 1 2 3 5 6 7 8 9 10 11 13 14 15 16 17 18 19 20 21 22 23 24 25 26 27 28 29 30 31 32 |
| 4 | 29 | 1 2 3 5 6 7 8 9 10 11 13 14 15 16 17 18 19 21 22 23 24 25 26 27 28 29 30 31 32 |
| 5 | 28 | 1 2 3 5 6 7 8 9 10 11 13 14 15 16 18 19 21 22 23 24 25 26 27 28 29 30 31 32 |
| 6 | 27 | 1 2 3 5 6 7 8 9 10 11 13 14 15 16 18 19 21 22 23 24 25 26 27 29 30 31 32 |
| 7 | 26 | 1 2 3 5 6 7 8 9 10 11 13 14 15 16 18 19 21 22 23 24 26 27 29 30 31 32 |
| 8 | 25 | 1 2 3 5 6 7 8 10 11 13 14 15 16 18 19 21 22 23 24 26 27 29 30 31 32 |
| 9 | 24 | 1 2 3 5 6 7 8 10 11 13 14 15 16 18 19 21 22 23 24 27 29 30 31 32 |
| 10 | 23 | 1 2 3 5 6 7 8 10 11 13 14 15 16 18 19 21 22 23 24 27 29 30 32 |
| 11 | 22 | 2 3 5 6 7 8 10 11 13 14 15 16 18 19 21 22 23 24 27 29 30 32 |
| 12 | 21 | 2 3 5 6 7 8 10 11 13 14 15 16 18 19 21 22 24 27 29 30 32 |
| 13 | 20 | 2 3 5 6 8 10 11 13 14 15 16 18 19 21 22 24 27 29 30 32 |
| 14 | 19 | 3 5 6 8 10 11 13 14 15 16 18 19 21 22 24 27 29 30 32 |
| 15 | 18 | 3 5 6 8 10 11 13 14 15 16 18 19 21 22 24 29 30 32 |
| 16 | 17 | 3 5 6 8 10 11 13 14 15 16 18 21 22 24 29 30 32 |
| 17 | 16 | 3 5 6 8 10 13 14 15 16 18 21 22 24 29 30 32 |
| 18 | 15 | 5 6 8 10 13 14 15 16 18 21 22 24 29 30 32 |
| 19 | 14 | 5 8 10 13 14 15 16 18 21 22 24 29 30 32 |
| 20 | 13 | 5 8 10 13 15 16 18 21 22 24 29 30 32 |
| 21 | 12 | 5 8 10 13 15 16 18 22 24 29 30 32 |
| 22 | 11 | 5 8 13 15 16 18 22 24 29 30 32 |
| 23 | 10 | 5 8 15 16 18 22 24 29 30 32 |
| 24 | 9 | 5 8 15 16 18 22 24 30 32 |
| 25 | 8 | 5 8 16 18 22 24 30 32 |
| 26 | 7 | 8 16 18 22 24 30 32 |
| 27 | 6 | 16 18 22 24 30 32 |
| 28 | 5 | 18 22 24 30 32 |
| 29 | 4 | 18 24 30 32 |
| 30 | 3 | 18 30 32 |
| 31 | 2 | 18 30 |
| 32 | 1 | 18 |


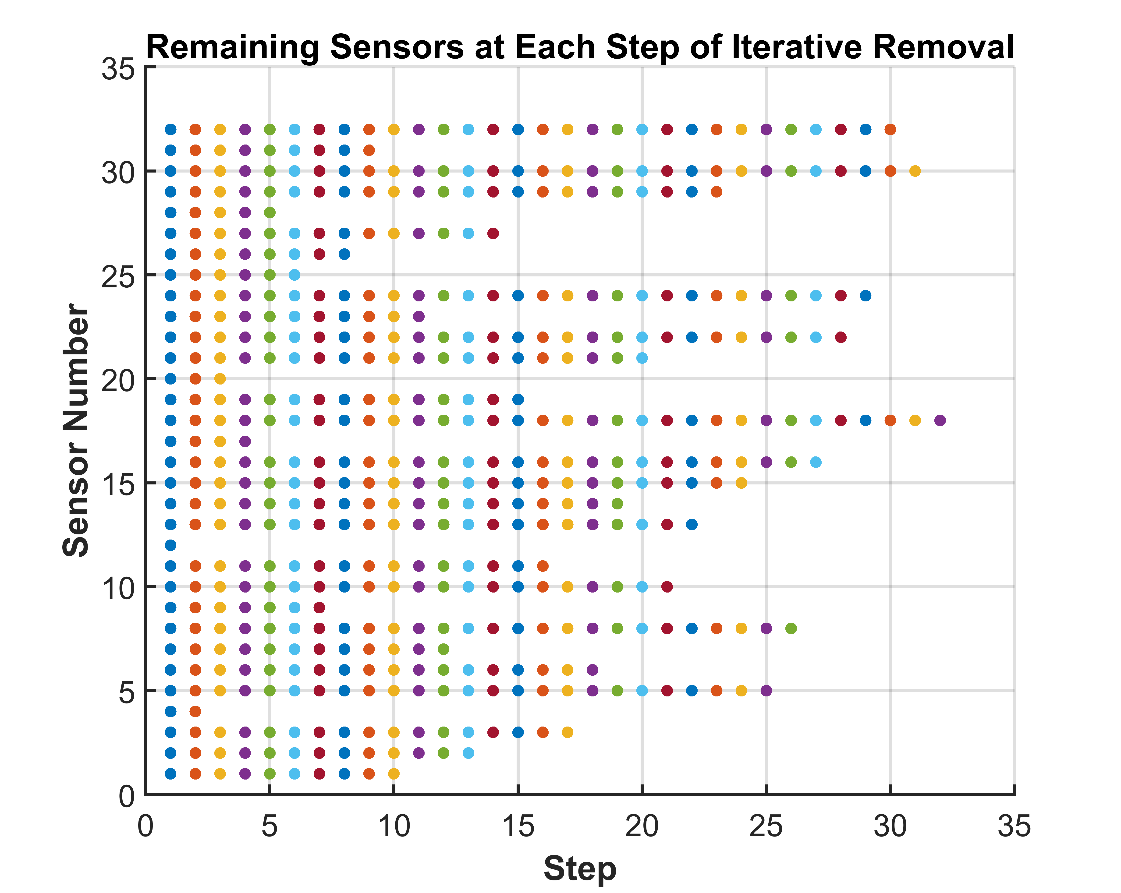


**Figure S8**. Graphical representation of **Table S4**.

**Table S5**. Evolution of validation and test performance metrics over 32 steps of the iterative sensor-elimination procedure for animal vs. human classification (CASE II). Bold red: step selected.

| **Step** | **Train Accuracy** | **Train Sensitivity** | **Train Specificity** | **Test Accuracy** | **Test Sensitivity** | **Test Specificity** |
| --- | --- | --- | --- | --- | --- | --- |
| 1 | 0.93700 | 0.93155 | 0.94246 | 0.94866 | 0.93750 | 0.95982 |
| 2 | 0.93625 | 0.93088 | 0.94163 | 0.93548 | 0.92627 | 0.94470 |
| 3 | 0.94709 | 0.93915 | 0.95503 | 0.95000 | 0.95714 | 0.94286 |
| 4 | 0.94773 | 0.93870 | 0.95676 | 0.97044 | 0.98030 | 0.96059 |
| 5 | 0.94870 | 0.93991 | 0.95748 | 0.93878 | 0.93367 | 0.94388 |
| **6** | **0.96767** | **0.96649** | **0.96884** | **0.96032** | **0.93122** | **0.98942** |
| 7 | 0.94994 | 0.94017 | 0.95971 | 0.95330 | 0.96154 | 0.94505 |
| 8 | 0.95333 | 0.95048 | 0.95619 | 0.94857 | 0.94286 | 0.95429 |
| 9 | 0.95503 | 0.94511 | 0.96495 | 0.95238 | 0.95238 | 0.95238 |
| 10 | 0.95307 | 0.94479 | 0.96135 | 0.95342 | 0.95031 | 0.95652 |
| 11 | 0.95779 | 0.95238 | 0.96320 | 0.96753 | 0.96104 | 0.97403 |
| 12 | 0.97354 | 0.97203 | 0.97506 | 0.98299 | 0.97959 | 0.98639 |
| 13 | 0.97143 | 0.96508 | 0.97778 | 0.97143 | 0.95714 | 0.98571 |
| 14 | 0.96282 | 0.96157 | 0.96408 | 0.98496 | 0.97744 | 0.99248 |
| 15 | 0.96781 | 0.96561 | 0.97002 | 0.97619 | 0.98413 | 0.96825 |
| 16 | 0.97199 | 0.96452 | 0.97946 | 0.95378 | 0.94958 | 0.95798 |
| 17 | 0.97222 | 0.96528 | 0.97917 | 0.96875 | 0.96429 | 0.97321 |
| 18 | 0.95661 | 0.95344 | 0.95979 | 0.93810 | 0.94286 | 0.93333 |
| 19 | 0.95351 | 0.94671 | 0.96032 | 0.97959 | 0.9898 | 0.96939 |
| 20 | 0.96642 | 0.95971 | 0.97314 | 0.97802 | 0.97802 | 0.97802 |
| 21 | 0.95899 | 0.95767 | 0.96032 | 0.97024 | 0.96429 | 0.97619 |
| 22 | 0.95094 | 0.94661 | 0.95527 | 0.96753 | 0.97403 | 0.96104 |
| 23 | 0.95476 | 0.94603 | 0.96349 | 0.92857 | 0.91429 | 0.94286 |
| 24 | 0.96561 | 0.96296 | 0.96825 | 0.96825 | 0.95238 | 0.98413 |
| 25 | 0.95635 | 0.95635 | 0.95635 | 0.96429 | 0.94643 | 0.98214 |
| 26 | 0.96372 | 0.95692 | 0.97052 | 0.96939 | 0.95918 | 0.97959 |
| 27 | 0.96825 | 0.96032 | 0.97619 | 0.98810 | 1.00000 | 0.97619 |
| 28 | 0.97143 | 0.96190 | 0.98095 | 0.97143 | 1.00000 | 0.94286 |
| 29 | 0.95635 | 0.95635 | 0.95635 | 1.00000 | 1.00000 | 1.00000 |
| 30 | 0.92593 | 0.92063 | 0.93122 | 0.90476 | 0.85714 | 0.95238 |
| 31 | 0.94841 | 0.95238 | 0.94444 | 0.92857 | 0.85714 | 1.00000 |
| 32 | 0.95238 | 0.95238 | 0.95238 | 0.92857 | 0.85714 | 1.00000 |

**Figure S9**. Graphical representation of **Table S5**.


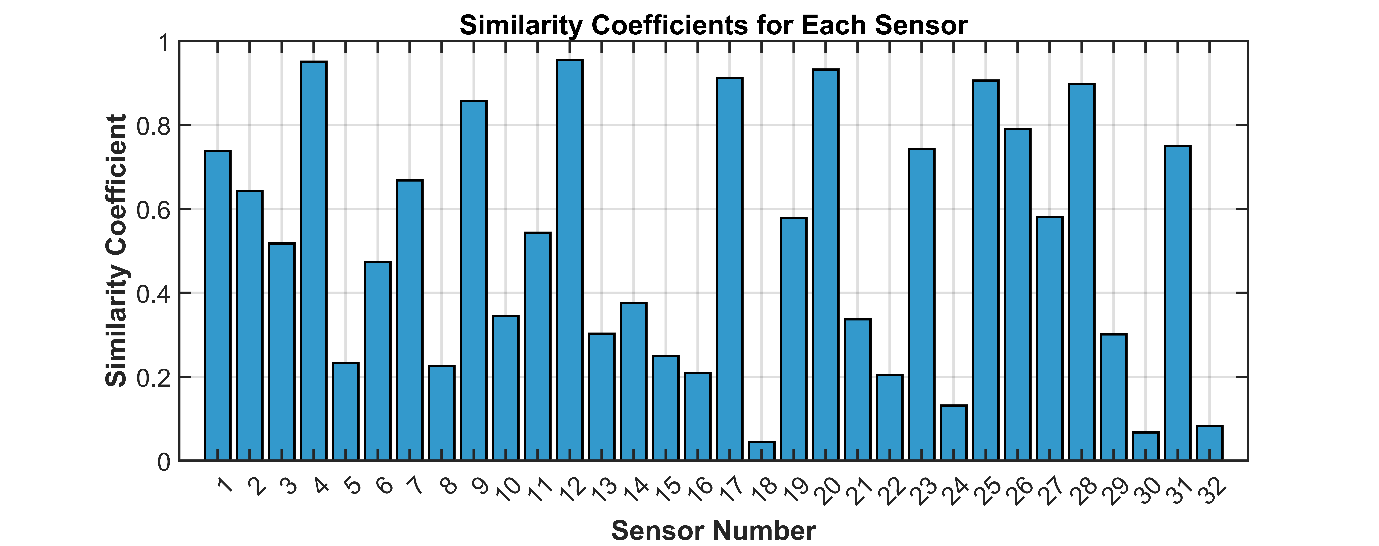


**Figure S10**. Similarity coefficients across the 32-sensor array discriminating animal vs. human biosamples, calculated using the training dataset. Bars denote the mean Pearson correlations between all inter-class signal pairs for each sensor.


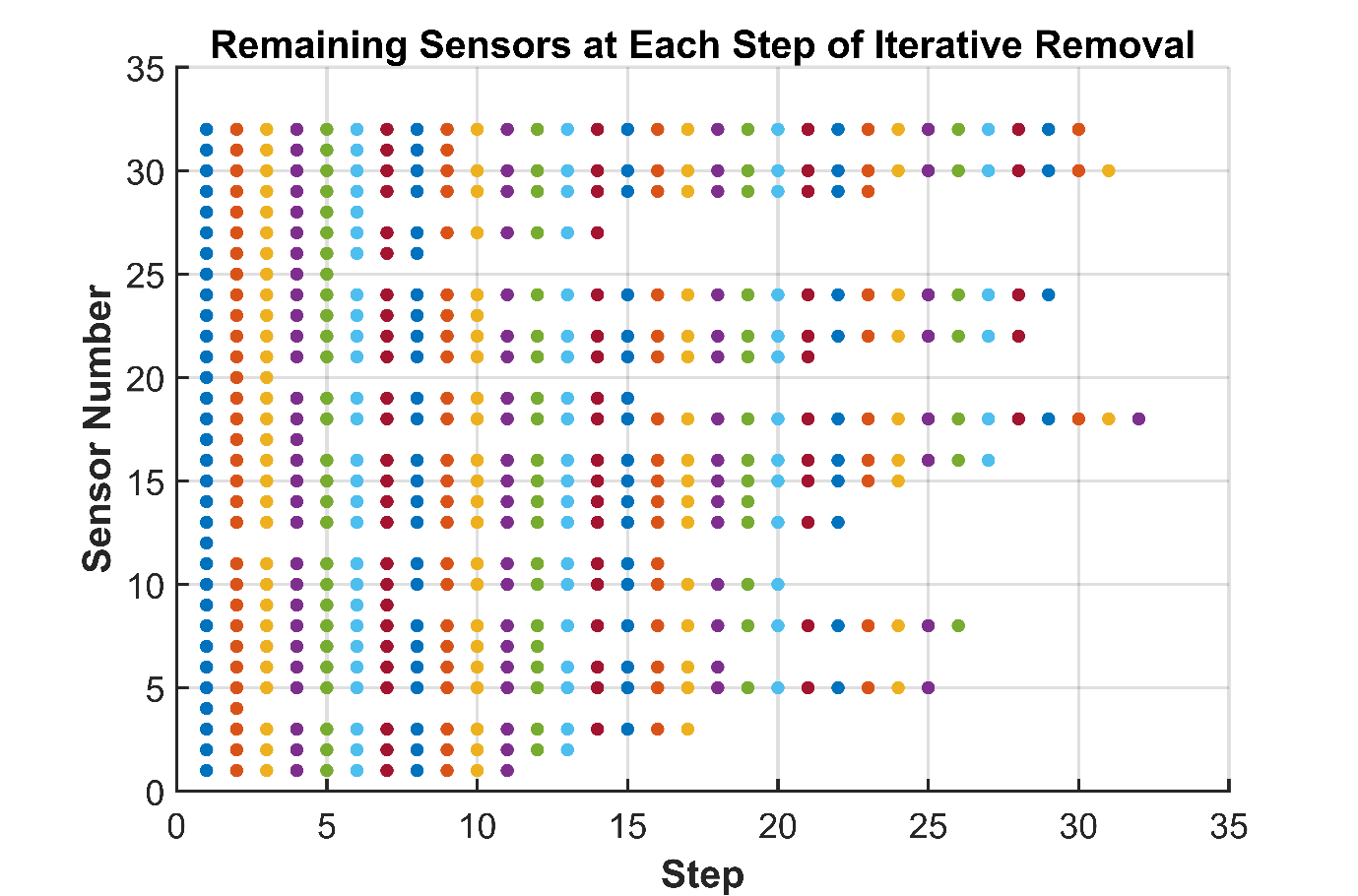


**Figure S11**. Graphical representation of sensor retention across removal steps (applied to training dataset) for animal vs. human discrimination (CASE II).


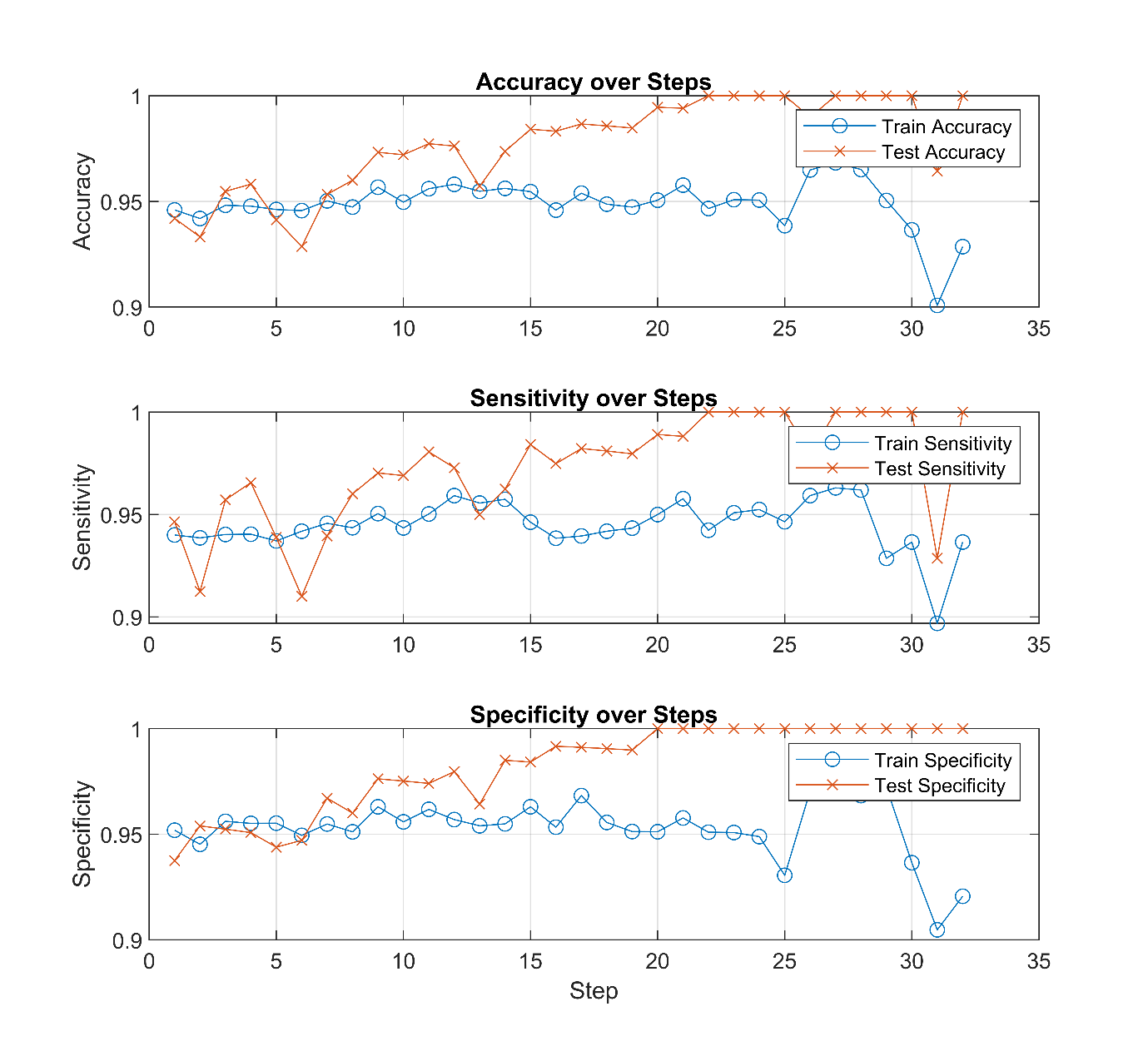


**Figure S12**. Model performance metrics (accuracy, sensitivity, specificity) plotted against the number of sensors retained for CASE II, with the sensor utility ranking algorithm applied to the training dataset only.


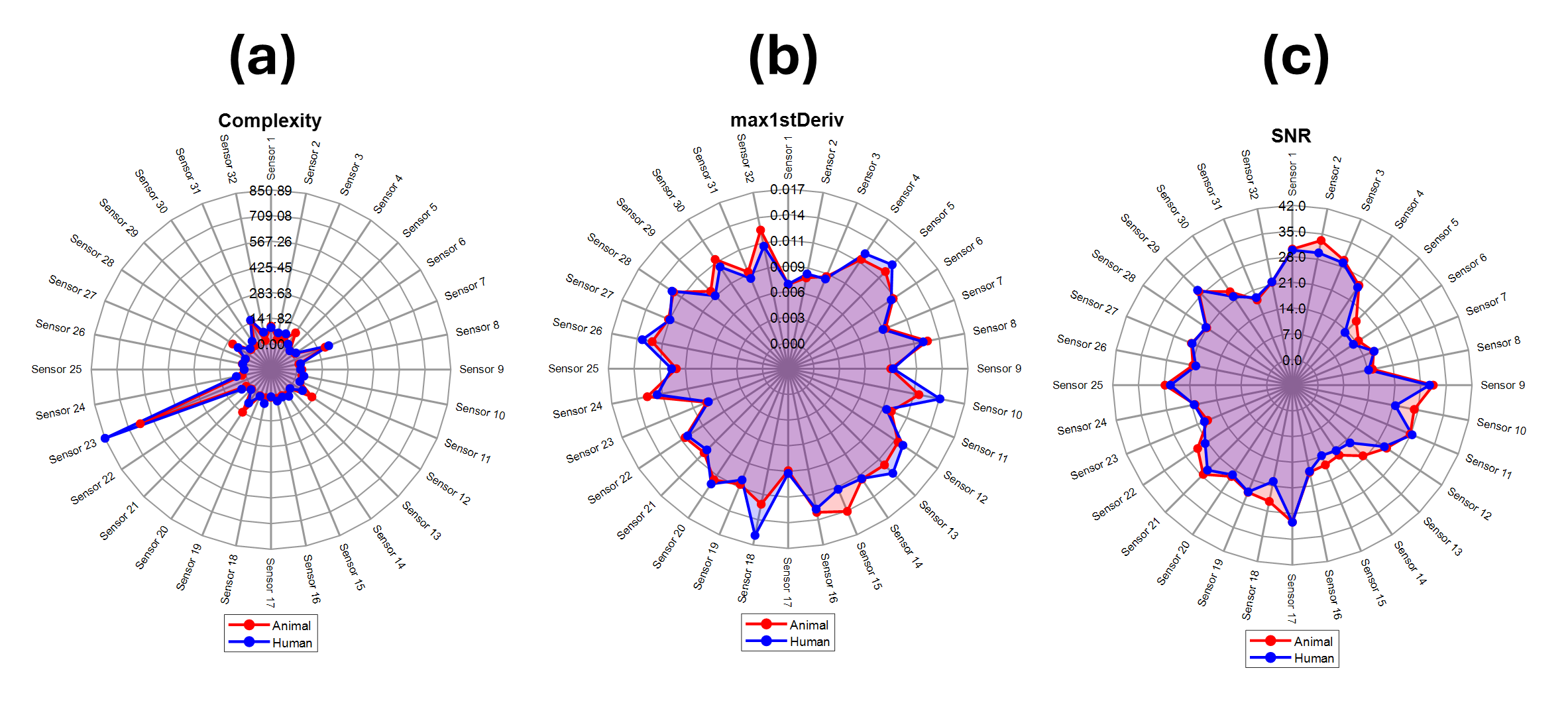


**Figure S13**. Radar plots showing the average values of key features— (a) *complexity*, (b) *max1stDeriv*, and (c) *SNR*— across all 32 sensors for animal and human biosample classes, highlighting their contributions to class differentiation.


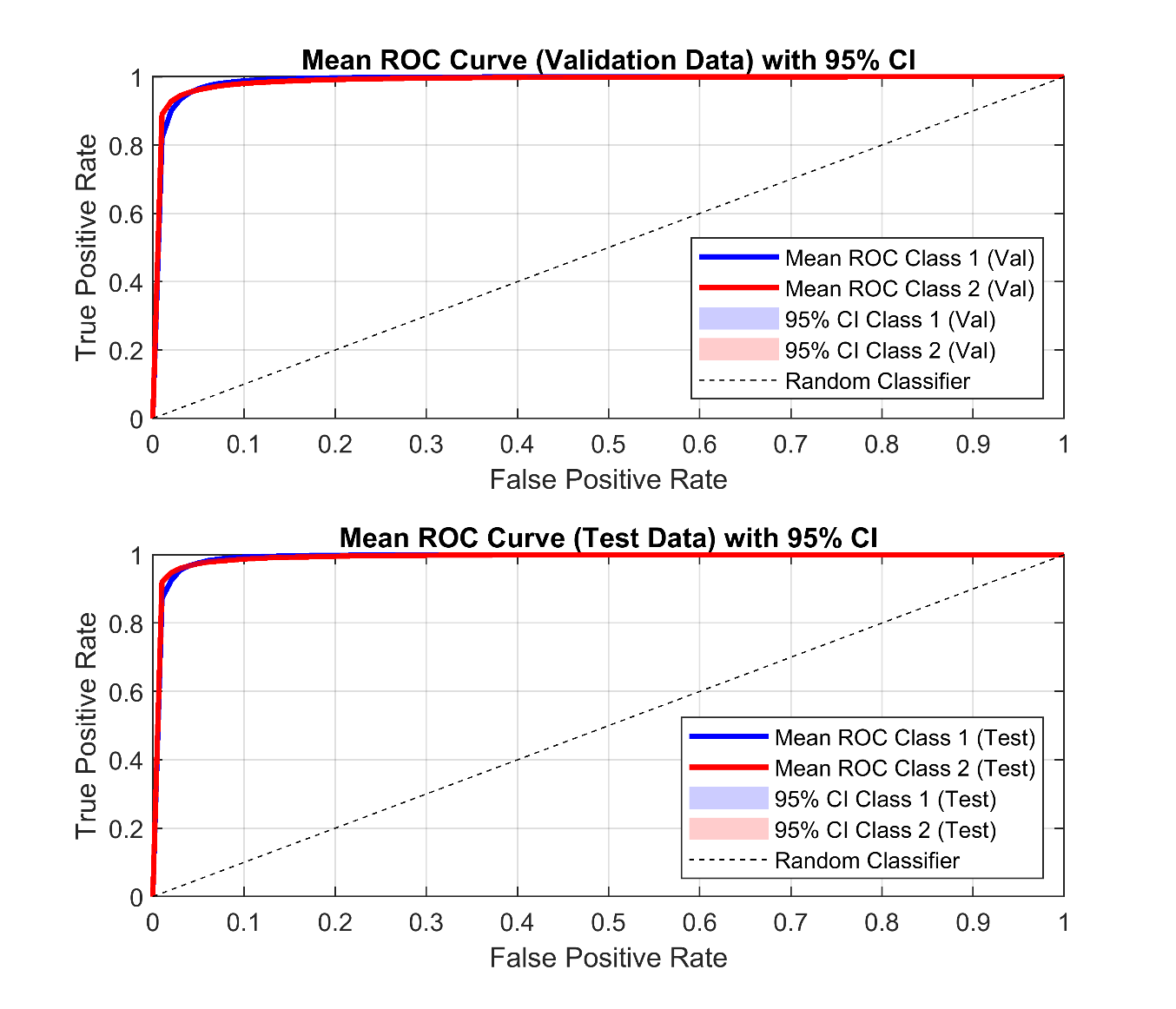


**Figure S14**. Mean ROC curves with 95% CIs for validation and test datasets (Classifier A1), showing robust classification of animal samples at PMI: 1–3 days (Class 1) and PMI: 4–32 days (Class 2).


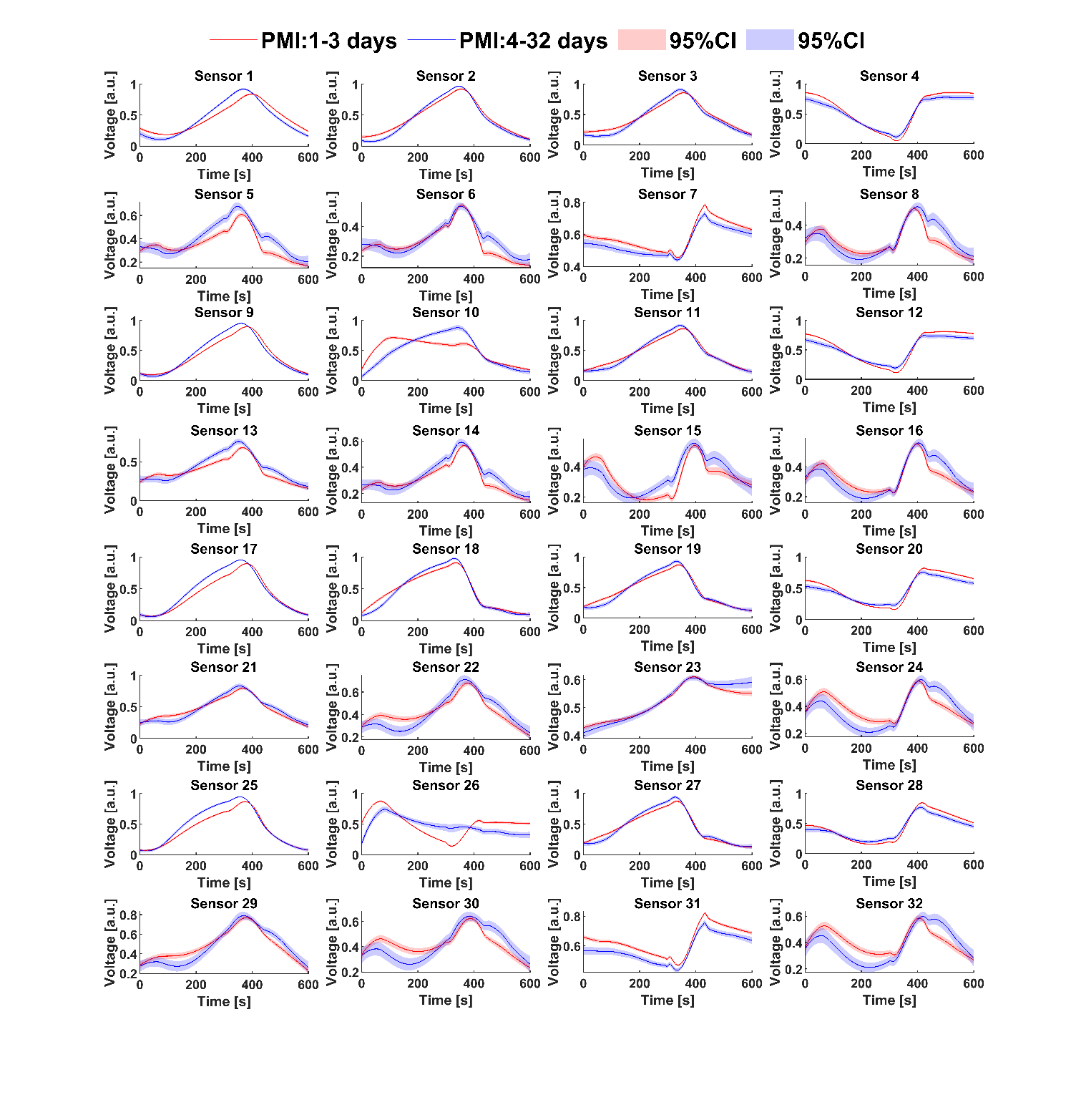


**Figure S15**. Multi-sensor response patterns of the *e*-nose to VOCs from animal samples at different PMIs (Classifier A1). The panels show normalized voltage signals with 95% CIs for 32 MOS sensors. Red: 1–3 days; Blue: 4–32 days PMIs. Distinct response patterns highlight the e-nose ability to differentiate between classes based on their respective VOC profiles.


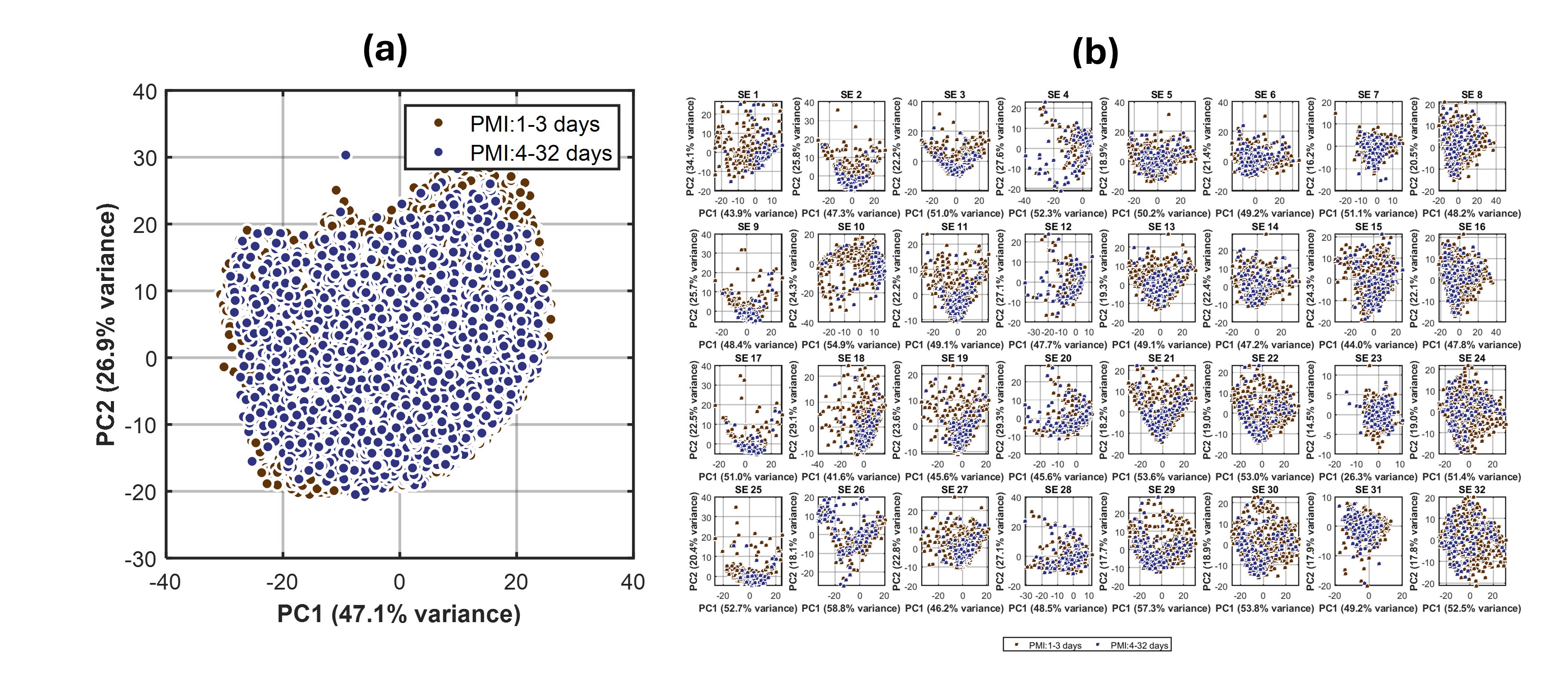


**Figure S16**. a) PCA plot of the full dataset, showing poor class separation (Classifier A1). b) Individual PCA plots for all 32 sensors, highlighting the contribution of each sensor to class differentiation.


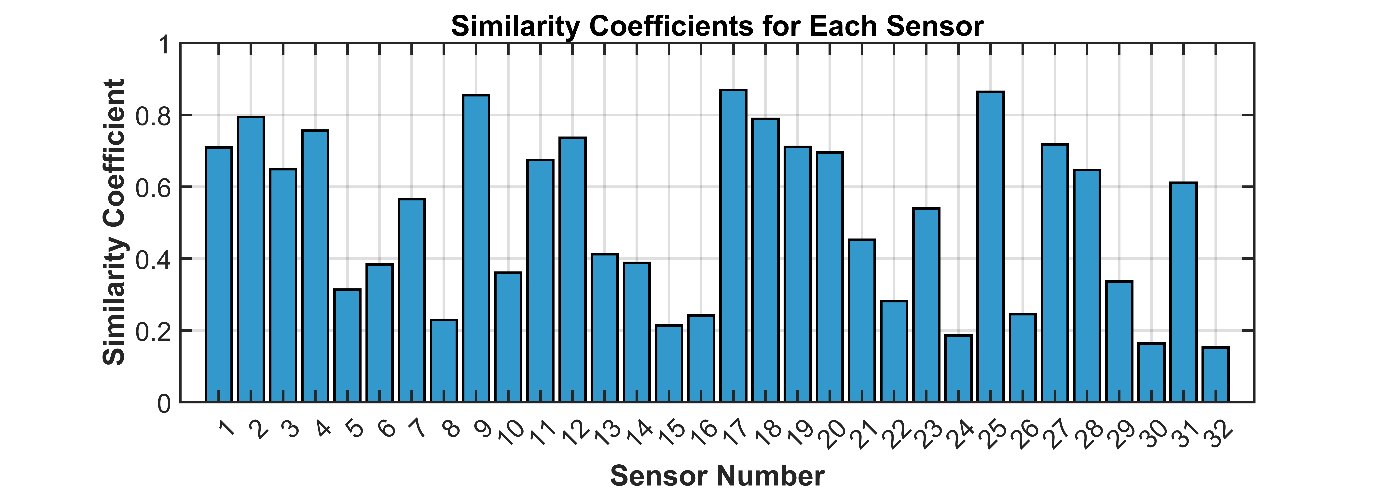


**Figure S17.** Similarity coefficients across the 32-sensor array discriminating animal samples at different PMIs (Classifier A1). Bars denote the mean Pearson correlations between all inter-class signal pairs for each sensor. Lower similarity coefficients indicate greater discriminatory power between classes.

**Table S6**. Sensors retained at each iteration of the sensor-elimination procedure for PMI: 1–3 days vs. PMI: 4–32 days classification (Classifier A1).

| **Step** | **No. of Sensors** | **Remaining Sensors at Each Step of Iterative Removal** |
| --- | --- | --- |
| 1 | 32 | 1 2 3 4 5 6 7 8 9 10 11 12 13 14 15 16 17 18 19 20 21 22 23 24 25 26 27 28 29 30 31 32 |
| 2 | 31 | 1 2 3 4 5 6 7 8 9 10 11 12 13 14 15 16 18 19 20 21 22 23 24 25 26 27 28 29 30 31 32 |
| 3 | 30 | 1 2 3 4 5 6 7 8 9 10 11 12 13 14 15 16 18 19 20 21 22 23 24 26 27 28 29 30 31 32 |
| 4 | 29 | 1 2 3 4 5 6 7 8 10 11 12 13 14 15 16 18 19 20 21 22 23 24 26 27 28 29 30 31 32 |
| 5 | 28 | 1 3 4 5 6 7 8 10 11 12 13 14 15 16 18 19 20 21 22 23 24 26 27 28 29 30 31 32 |
| 6 | 27 | 1 3 4 5 6 7 8 10 11 12 13 14 15 16 19 20 21 22 23 24 26 27 28 29 30 31 32 |
| 7 | 26 | 1 3 5 6 7 8 10 11 12 13 14 15 16 19 20 21 22 23 24 26 27 28 29 30 31 32 |
| 8 | 25 | 1 3 5 6 7 8 10 11 13 14 15 16 19 20 21 22 23 24 26 27 28 29 30 31 32 |
| 9 | 24 | 1 3 5 6 7 8 10 11 13 14 15 16 19 20 21 22 23 24 26 28 29 30 31 32 |
| 10 | 23 | 1 3 5 6 7 8 10 11 13 14 15 16 20 21 22 23 24 26 28 29 30 31 32 |
| 11 | 22 | 3 5 6 7 8 10 11 13 14 15 16 20 21 22 23 24 26 28 29 30 31 32 |
| 12 | 21 | 3 5 6 7 8 10 11 13 14 15 16 21 22 23 24 26 28 29 30 31 32 |
| 13 | 20 | 3 5 6 7 8 10 13 14 15 16 21 22 23 24 26 28 29 30 31 32 |
| 14 | 19 | 5 6 7 8 10 13 14 15 16 21 22 23 24 26 28 29 30 31 32 |
| 15 | 18 | 5 6 7 8 10 13 14 15 16 21 22 23 24 26 29 30 31 32 |
| 16 | 17 | 5 6 7 8 10 13 14 15 16 21 22 23 24 26 29 30 32 |
| 17 | 16 | 5 6 8 10 13 14 15 16 21 22 23 24 26 29 30 32 |
| 18 | 15 | 5 6 8 10 13 14 15 16 21 22 24 26 29 30 32 |
| 19 | 14 | 5 6 8 10 13 14 15 16 22 24 26 29 30 32 |
| 20 | 13 | 5 6 8 10 14 15 16 22 24 26 29 30 32 |
| 21 | 12 | 5 6 8 10 15 16 22 24 26 29 30 32 |
| 22 | 11 | 5 8 10 15 16 22 24 26 29 30 32 |
| 23 | 10 | 5 8 15 16 22 24 26 29 30 32 |
| 24 | 9 | 5 8 15 16 22 24 26 30 32 |
| 25 | 8 | 8 15 16 22 24 26 30 32 |
| 26 | 7 | 8 15 16 24 26 30 32 |
| 27 | 6 | 8 15 16 24 30 32 |
| 28 | 5 | 8 15 24 30 32 |
| 29 | 4 | 15 24 30 32 |
| 30 | 3 | 24 30 32 |
| 31 | 2 | 30 32 |
| 32 | 1 | 32 |


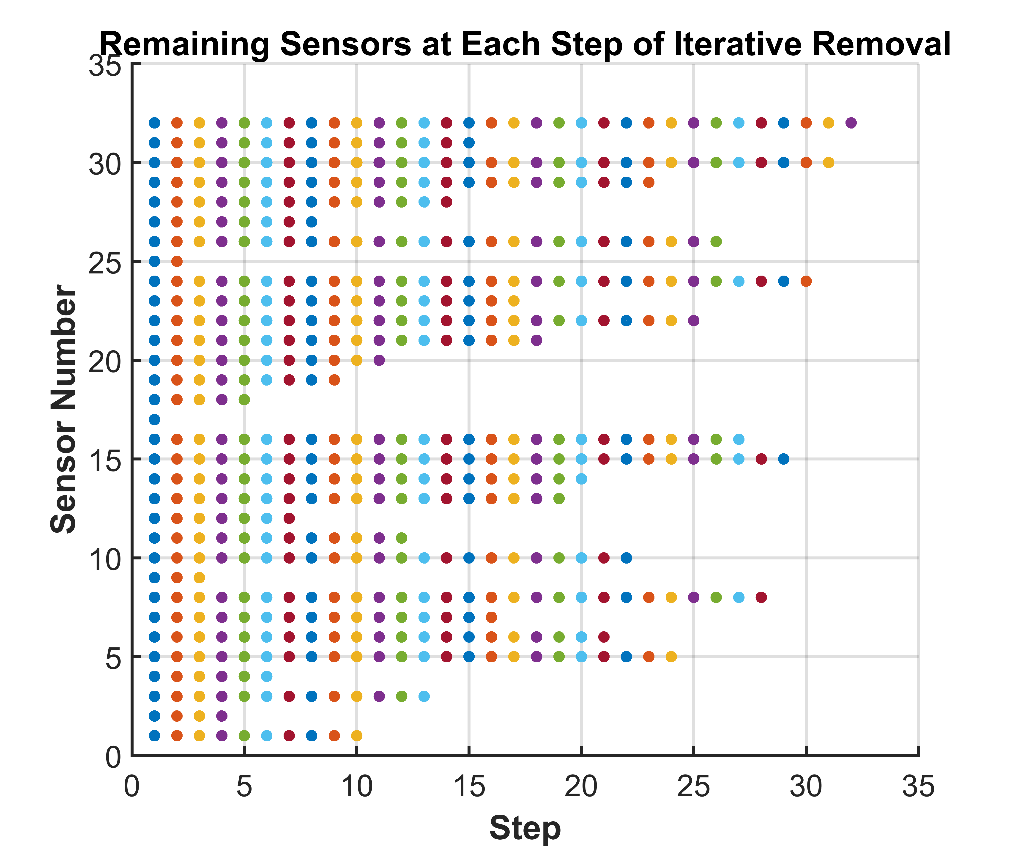


**Figure S18**. Graphical representation of **Table S6**.

**Table S7**. Evolution of validation and test performance metrics over 32 steps of the iterative sensor-elimination procedure for PMI: 1–3 days vs. PMI: 4–32 days classification (Classifier A1). Bold red: step selected.

| **Step** | **Train Accuracy** | **Train Sensitivity** | **Train Specificity** | **Test Accuracy** | **Test Sensitivity** | **Test Specificity** |
| --- | --- | --- | --- | --- | --- | --- |
| 1 | 0.88346 | 0.97338 | 0.57804 | 0.88981 | 0.97264 | 0.60845 |
| 2 | 0.88110 | 0.96825 | 0.58508 | 0.90212 | 0.97774 | 0.64535 |
| 3 | 0.88016 | 0.97102 | 0.57157 | 0.87773 | 0.96463 | 0.58258 |
| 4 | 0.89040 | 0.97124 | 0.61581 | 0.8947 | 0.97164 | 0.63354 |
| **5** | **0.96821** | **0.99358** | **0.88202** | **0.96779** | **0.99147** | **0.88746** |
| 6 | 0.96804 | 0.99029 | 0.89247 | 0.97874 | 0.99213 | 0.93333 |
| 7 | 0.96646 | 0.99263 | 0.87760 | 0.97082 | 0.99388 | 0.89236 |
| 8 | 0.96858 | 0.98963 | 0.89712 | 0.97131 | 0.99258 | 0.89892 |
| 9 | 0.96974 | 0.99263 | 0.89199 | 0.97182 | 0.98674 | 0.92105 |
| 10 | 0.95387 | 0.97975 | 0.86597 | 0.96702 | 0.98155 | 0.91765 |
| 11 | 0.95902 | 0.99169 | 0.84804 | 0.96365 | 0.99276 | 0.86475 |
| 12 | 0.98569 | 0.99691 | 0.94757 | 0.99121 | 0.99494 | 0.97854 |
| 13 | 0.98714 | 0.99602 | 0.95696 | 0.9918 | 0.99867 | 0.96847 |
| 14 | 0.96249 | 0.98961 | 0.87046 | 0.95361 | 0.99163 | 0.82381 |
| 15 | 0.96104 | 0.98805 | 0.86937 | 0.96925 | 0.99264 | 0.88945 |
| 16 | 0.96049 | 0.98838 | 0.8658 | 0.96984 | 0.99532 | 0.88298 |
| 17 | 0.96258 | 0.98876 | 0.87367 | 0.96282 | 0.99005 | 0.87006 |
| 18 | 0.94490 | 0.98821 | 0.79773 | 0.95219 | 0.98584 | 0.83832 |
| 19 | 0.94503 | 0.98800 | 0.79914 | 0.95022 | 0.98485 | 0.83226 |
| 20 | 0.94956 | 0.98345 | 0.83449 | 0.94164 | 0.97959 | 0.81250 |
| 21 | 0.95352 | 0.99263 | 0.82068 | 0.9453 | 0.99115 | 0.78947 |
| 22 | 0.95468 | 0.99143 | 0.82985 | 0.96082 | 0.99275 | 0.85246 |
| 23 | 0.96061 | 0.99322 | 0.84985 | 0.96926 | 0.98939 | 0.90090 |
| 24 | 0.9871 | 0.99935 | 0.94549 | 0.98633 | 1.00000 | 0.94000 |
| 25 | 0.99402 | 0.99963 | 0.97500 | 0.99487 | 1.00000 | 0.97727 |
| 26 | 0.99772 | 1.00000 | 0.99000 | 0.99707 | 0.99621 | 1.00000 |
| 27 | 0.99848 | 0.99951 | 0.99500 | 1.00000 | 1.00000 | 1.00000 |
| 28 | 0.99909 | 0.99882 | 1.00000 | 0.99590 | 1.00000 | 0.98214 |
| 29 | 1.00000 | 1.00000 | 1.00000 | 1.00000 | 1.00000 | 1.00000 |
| 30 | 1.00000 | 1.00000 | 1.00000 | 0.99315 | 0.99115 | 1.00000 |
| 31 | 0.99886 | 0.99853 | 1.00000 | 1.00000 | 1.00000 | 1.00000 |
| 32 | 1.00000 | 1.00000 | 1.00000 | 1.00000 | 1.00000 | 1.00000 |

**Figure S19**. Graphical representation of Table S7.


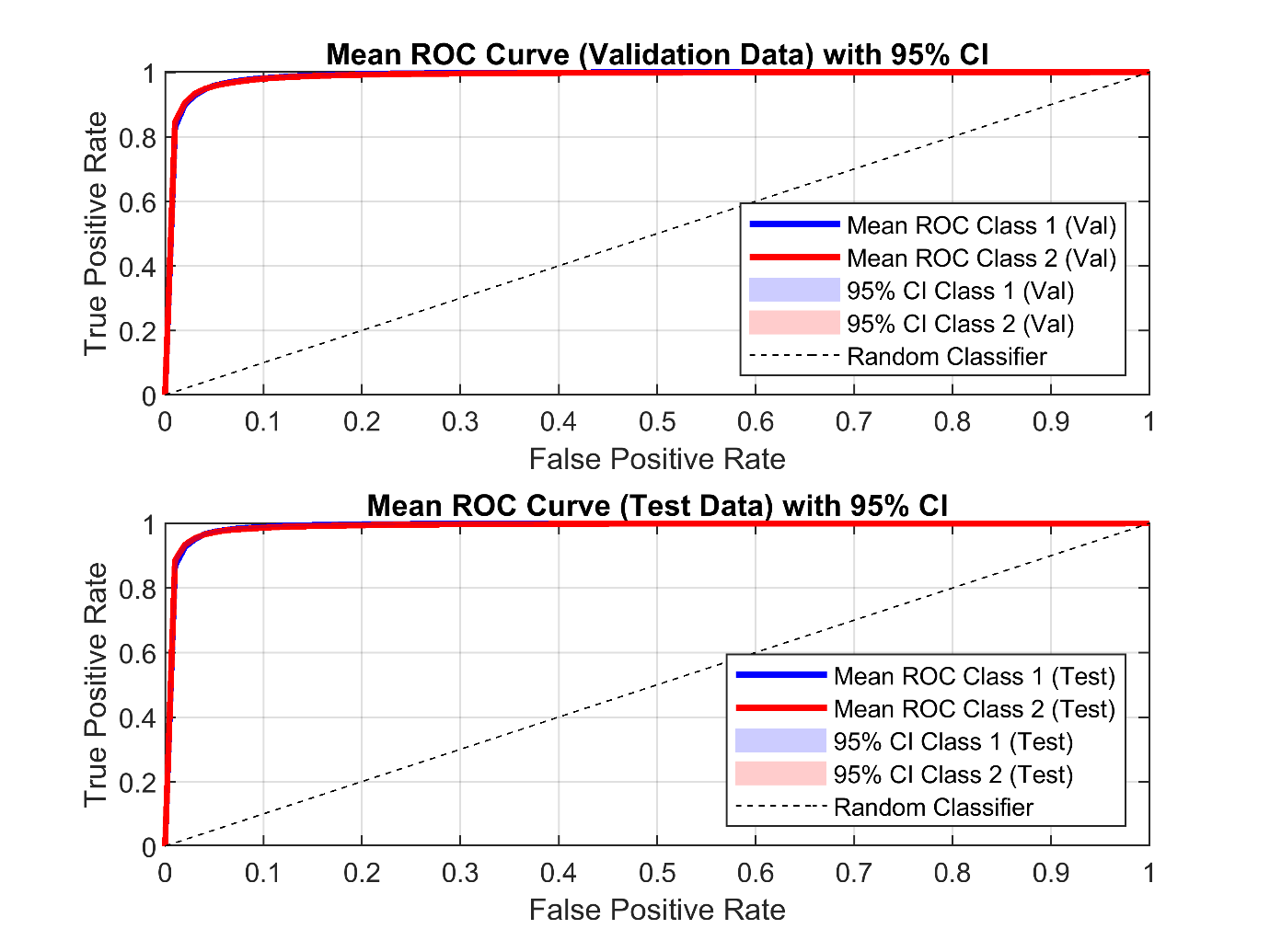


**Figure S20**. Mean ROC curves with 95% CIs for validation and test datasets (Classifier A2), showing robust classification of animal samples at PMI: 1 day (Class 1) and PMI: 2–3 days (Class 2).


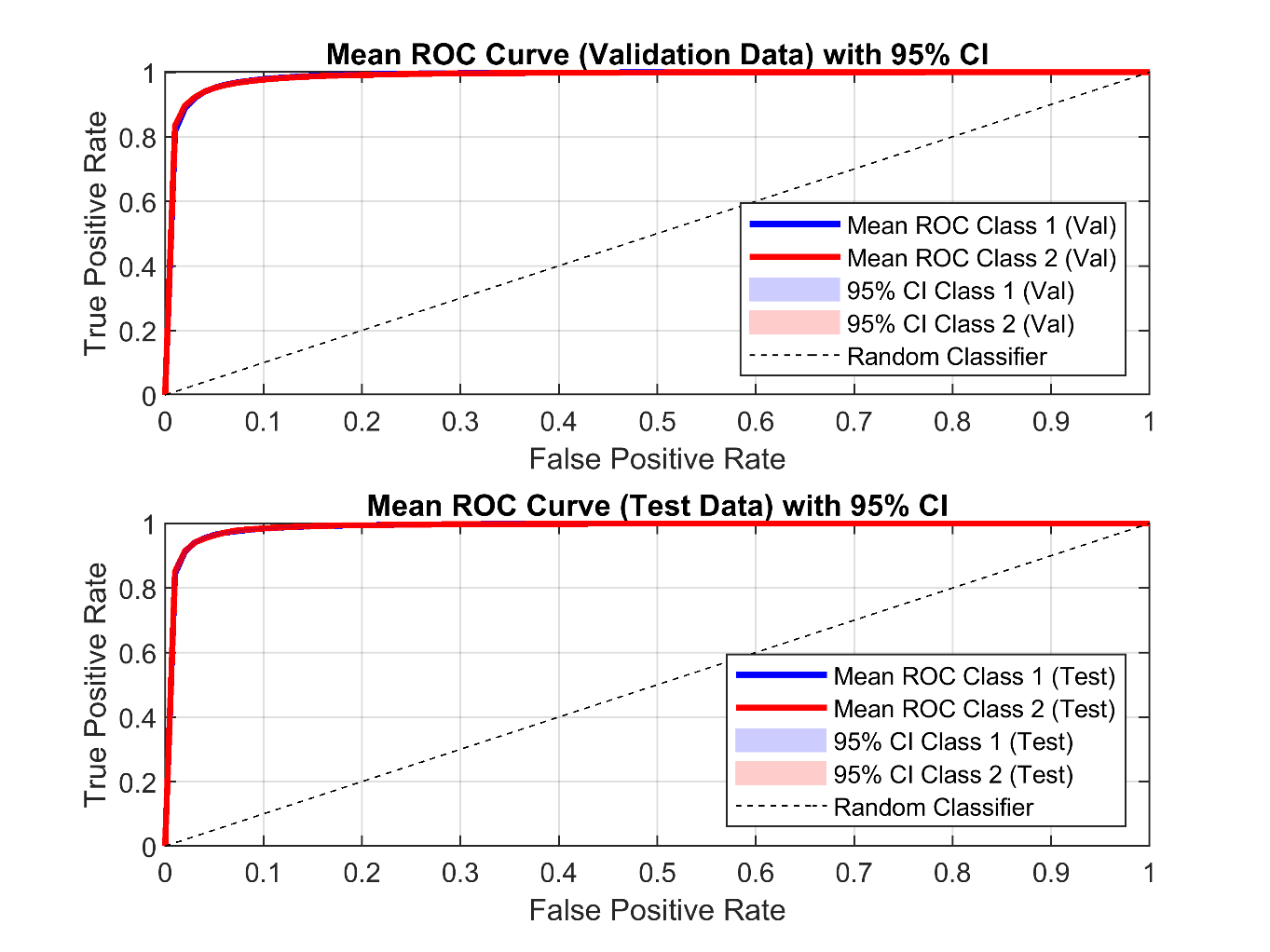


**Figure S21**. Mean ROC curves with 95% CIs for validation and test data (Classifier A3), showing robust classification of animal samples at PMI: 2 days (Class 1) and PMI: 3 days (Class 2).


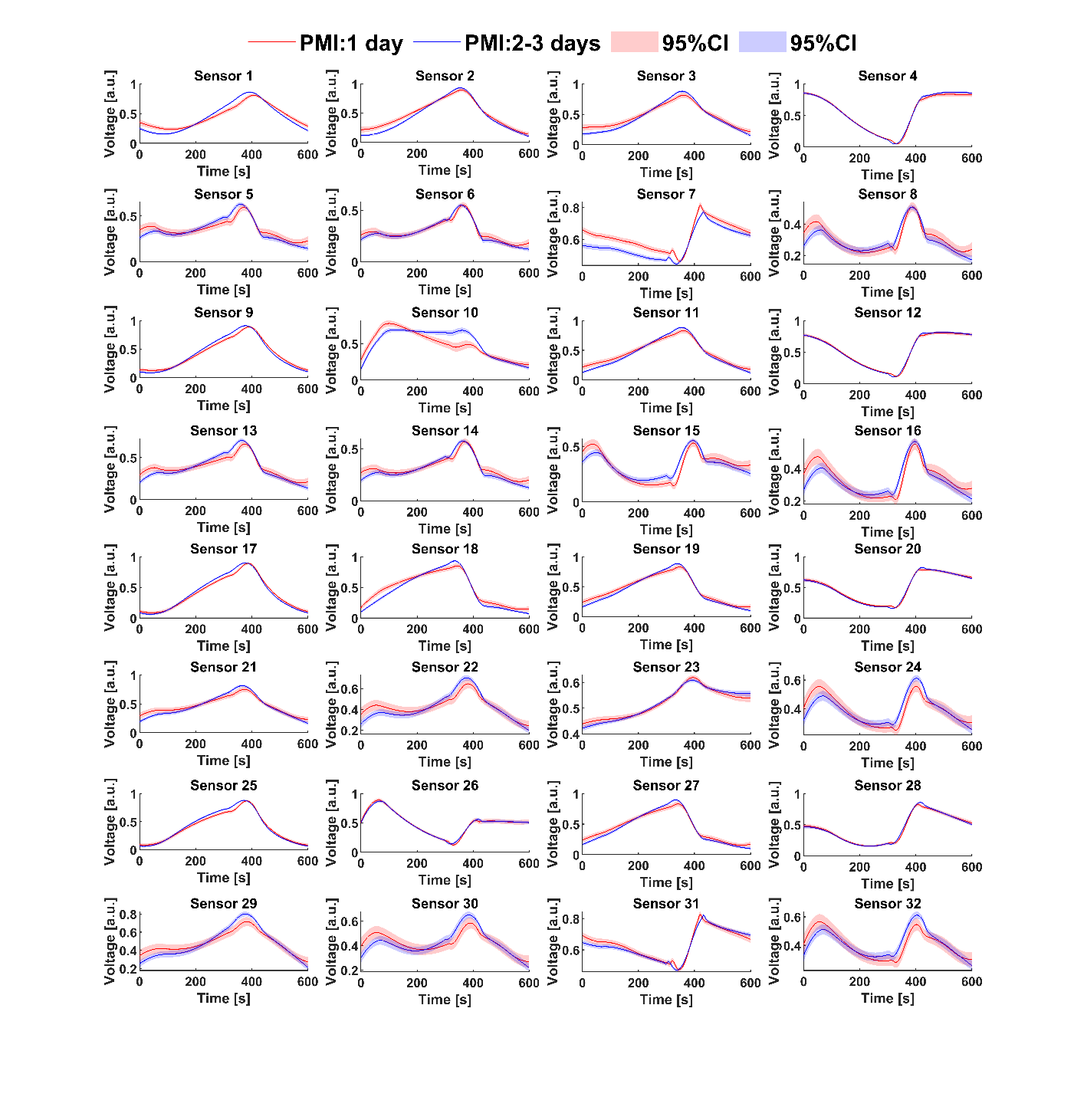


**Figure S22**. Multi-sensor response patterns of the *e*-nose to VOCs from animal samples at different PMIs (Classifier A2). The panels show normalized voltage signals with 95% CIs 32 MOS sensors. Red: 1 day; Blue: 2–3 days PMIs. Distinct response patterns highlight the e-nose ability to differentiate between classes based on their respective VOC profiles.


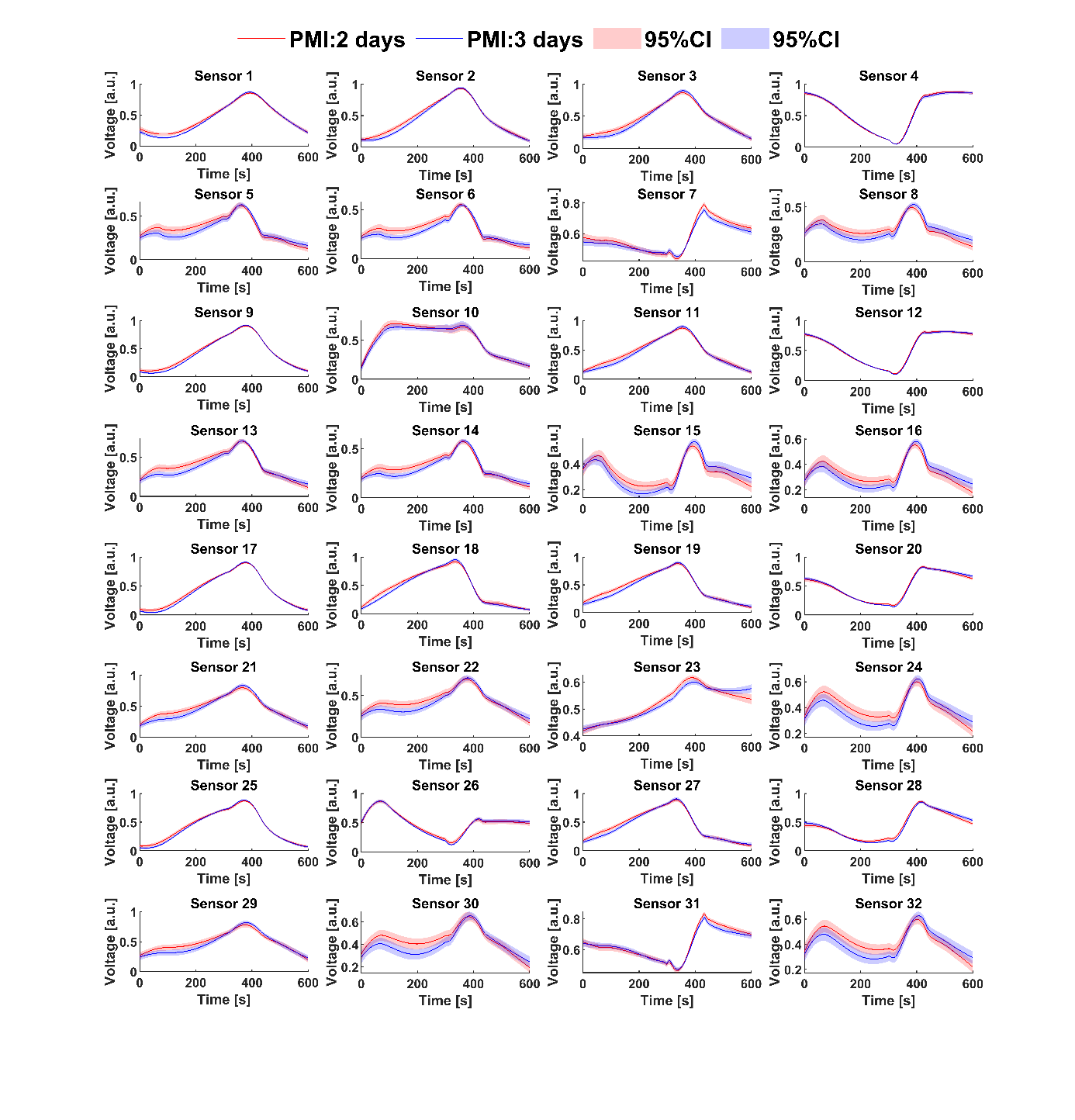


**Figure S23**. Multi-sensor response patterns of the *e*-nose to VOCs from animal samples at different PMIs (Classifier A3). The panels show normalized voltage signals with 95% CIs for all 32 MOS sensors. Red: 2 days; Blue: 3 days PMIs. Distinct response patterns highlight the e-nose ability to differentiate between classes based on their respective VOC profiles.


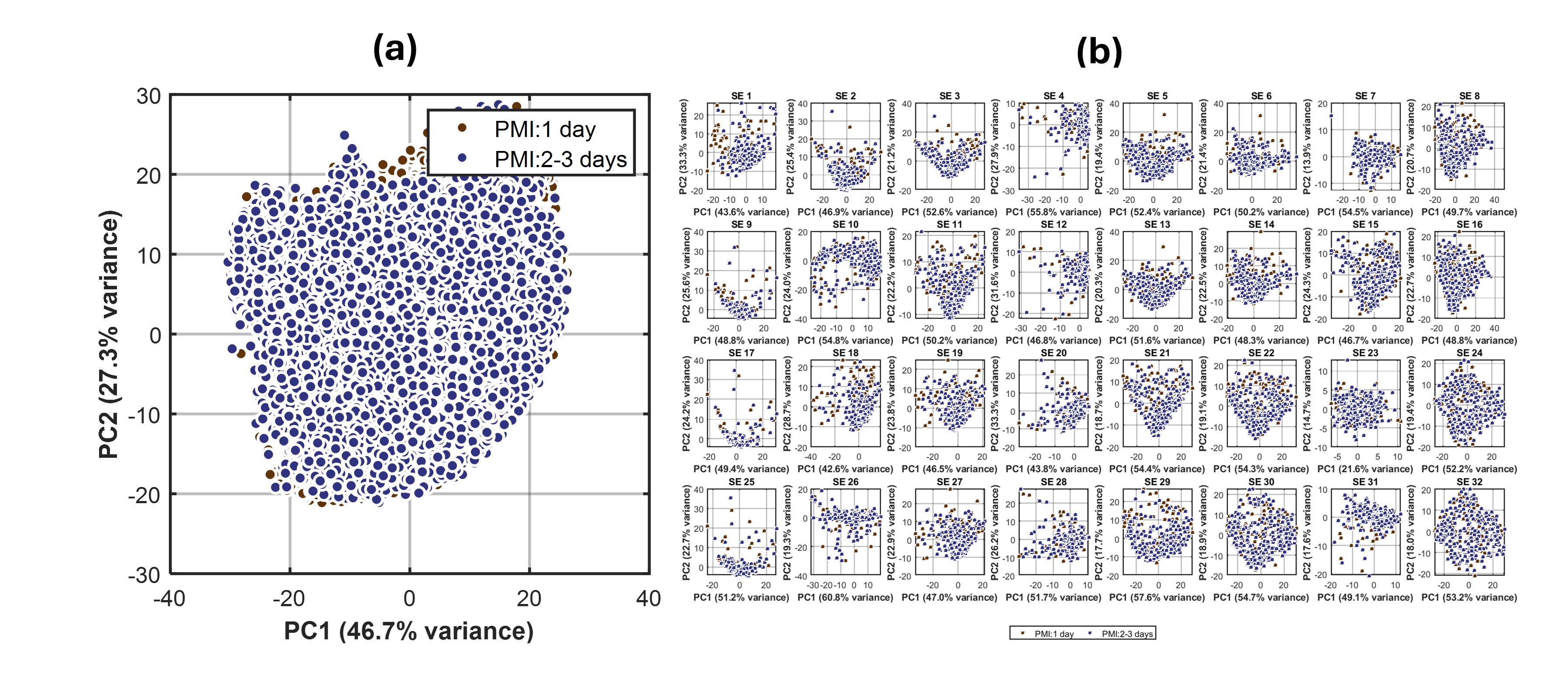


**Figure S24**. **Figure S16**. a) PCA plot of the full dataset, showing poor class separation (Classifier A2). b) Individual PCA plots for all 32 sensors, highlighting the contribution of each sensor to class differentiation.

**
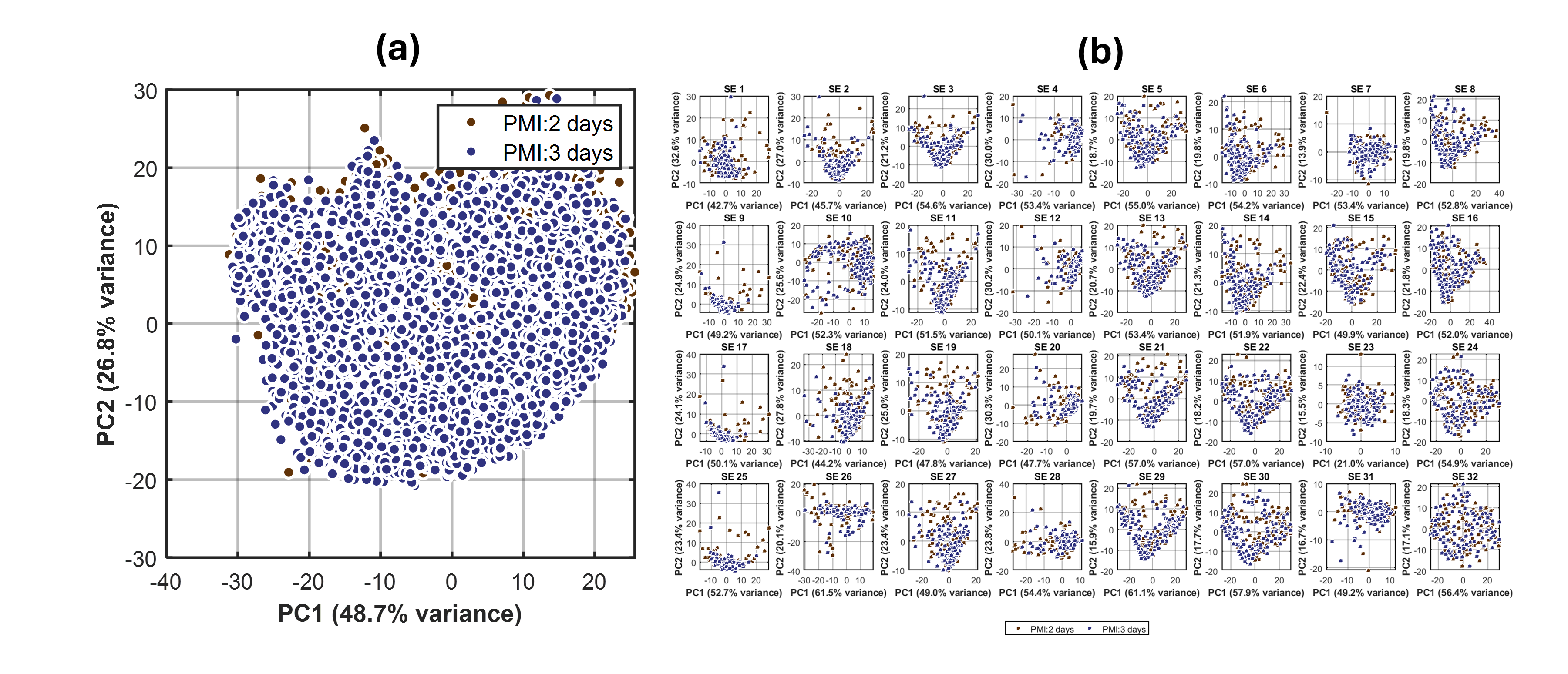
**

**Figure S25**. a) PCA plot of the full dataset, showing poor class separation (Classifier A3). b) Individual PCA plots for all 32 sensors, highlighting the contribution of each sensor to class differentiation.


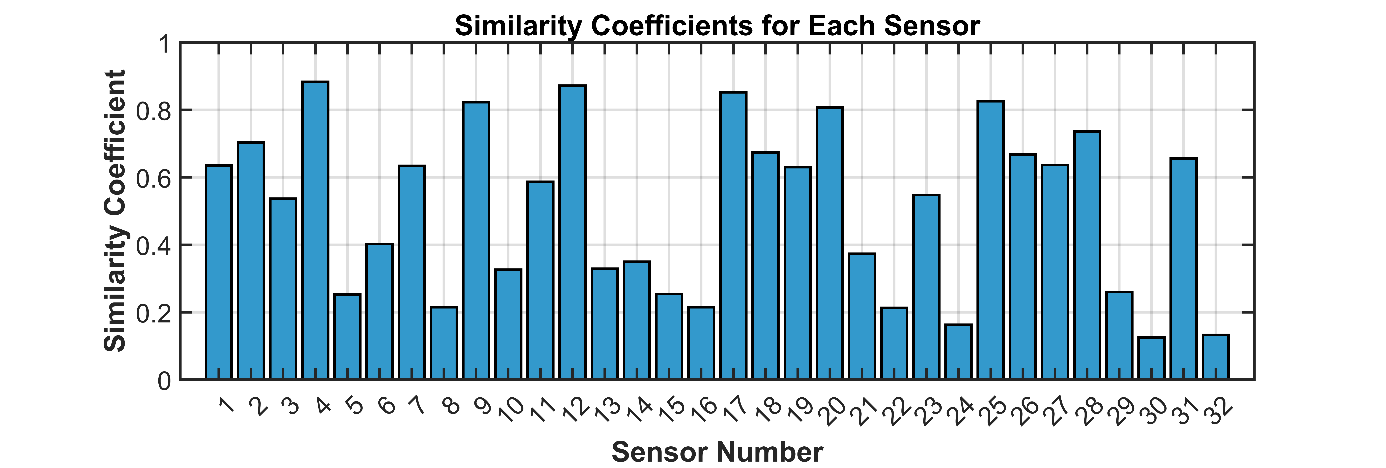


**Figure S26.** Similarity coefficients across the 32-sensor array discriminating animal samples at different PMIs (Classifier A2). Bars denote the mean Pearson correlations between all inter-class signal pairs for each sensor. Lower similarity coefficients indicate greater discriminatory power between classes.


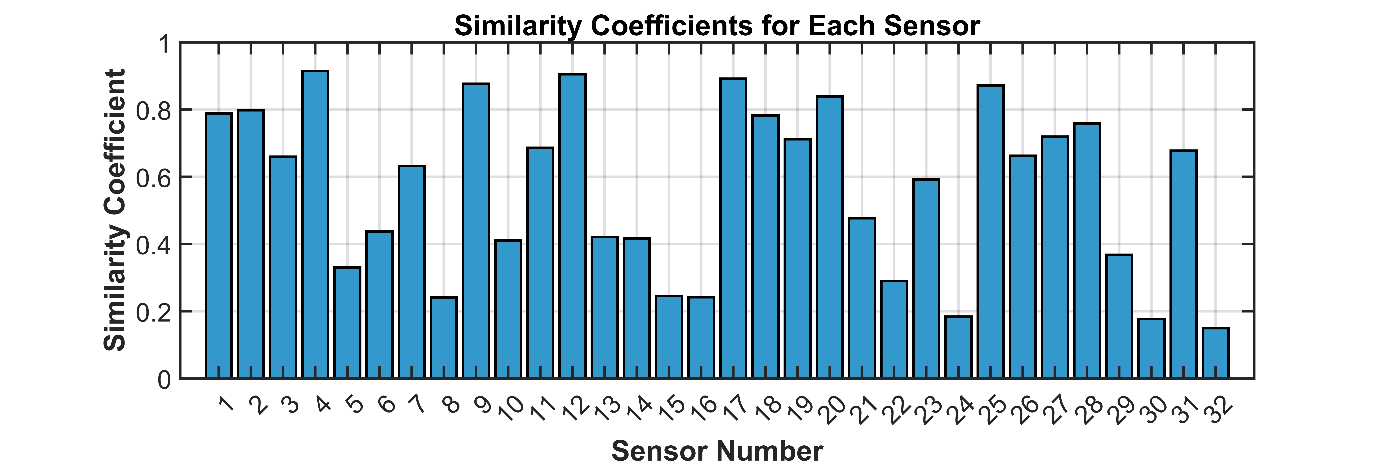


**Figure S27.** Similarity coefficients across the 32-sensor array discriminating animal samples at different PMIs (Classifier A3). Bars denote the mean Pearson correlations between all inter-class signal pairs for each sensor. Lower similarity coefficients indicate greater discriminatory power between classes.

**Table S8**. Sensors retained at each iteration of the sensor-elimination procedure for PMI: 1 day vs. PMI: 2–3 days classification (Classifier A2).

| **Step** | **No. of Sensors** | **Remaining Sensors at Each Step of Iterative Removal** |
| --- | --- | --- |
| 1 | 32 | 1 2 3 4 5 6 7 8 9 10 11 12 13 14 15 16 17 18 19 20 21 22 23 24 25 26 27 28 29 30 31 32 |
| 2 | 31 | 1 2 3 5 6 7 8 9 10 11 12 13 14 15 16 17 18 19 20 21 22 23 24 25 26 27 28 29 30 31 32 |
| 3 | 30 | 1 2 3 5 6 7 8 9 10 11 13 14 15 16 17 18 19 20 21 22 23 24 25 26 27 28 29 30 31 32 |
| 4 | 29 | 1 2 3 5 6 7 8 9 10 11 13 14 15 16 18 19 20 21 22 23 24 25 26 27 28 29 30 31 32 |
| 5 | 28 | 1 2 3 5 6 7 8 9 10 11 13 14 15 16 18 19 20 21 22 23 24 26 27 28 29 30 31 32 |
| 6 | 27 | 1 2 3 5 6 7 8 10 11 13 14 15 16 18 19 20 21 22 23 24 26 27 28 29 30 31 32 |
| 7 | 26 | 1 2 3 5 6 7 8 10 11 13 14 15 16 18 19 21 22 23 24 26 27 28 29 30 31 32 |
| 8 | 25 | 1 2 3 5 6 7 8 10 11 13 14 15 16 18 19 21 22 23 24 26 27 29 30 31 32 |
| 9 | 24 | 1 3 5 6 7 8 10 11 13 14 15 16 18 19 21 22 23 24 26 27 29 30 31 32 |
| 10 | 23 | 1 3 5 6 7 8 10 11 13 14 15 16 19 21 22 23 24 26 27 29 30 31 32 |
| 11 | 22 | 1 3 5 6 7 8 10 11 13 14 15 16 19 21 22 23 24 27 29 30 31 32 |
| 12 | 21 | 1 3 5 6 7 8 10 11 13 14 15 16 19 21 22 23 24 27 29 30 32 |
| 13 | 20 | 1 3 5 6 7 8 10 11 13 14 15 16 19 21 22 23 24 29 30 32 |
| 14 | 19 | 3 5 6 7 8 10 11 13 14 15 16 19 21 22 23 24 29 30 32 |
| 15 | 18 | 3 5 6 8 10 11 13 14 15 16 19 21 22 23 24 29 30 32 |
| 16 | 17 | 3 5 6 8 10 11 13 14 15 16 21 22 23 24 29 30 32 |
| 17 | 16 | 3 5 6 8 10 13 14 15 16 21 22 23 24 29 30 32 |
| 18 | 15 | 3 5 6 8 10 13 14 15 16 21 22 24 29 30 32 |
| 19 | 14 | 5 6 8 10 13 14 15 16 21 22 24 29 30 32 |
| 20 | 13 | 5 6 8 10 13 14 15 16 21 22 24 29 30 32 |
| 21 | 12 | 5 8 10 13 14 15 16 22 24 29 30 32 |
| 22 | 11 | 5 8 10 13 15 16 22 24 29 30 32 |
| 23 | 10 | 5 8 10 15 16 22 24 29 30 32 |
| 24 | 9 | 5 8 15 16 22 24 29 30 32 |
| 25 | 8 | 5 8 15 16 22 24 30 32 |
| 26 | 7 | 5 8 16 22 24 30 32 |
| 27 | 6 | 8 16 22 24 30 32 |
| 28 | 5 | 8 22 24 30 32 |
| 29 | 4 | 22 24 30 32 |
| 30 | 3 | 24 30 32 |
| 31 | 2 | 30 32 |
| 32 | 1 | 30 |


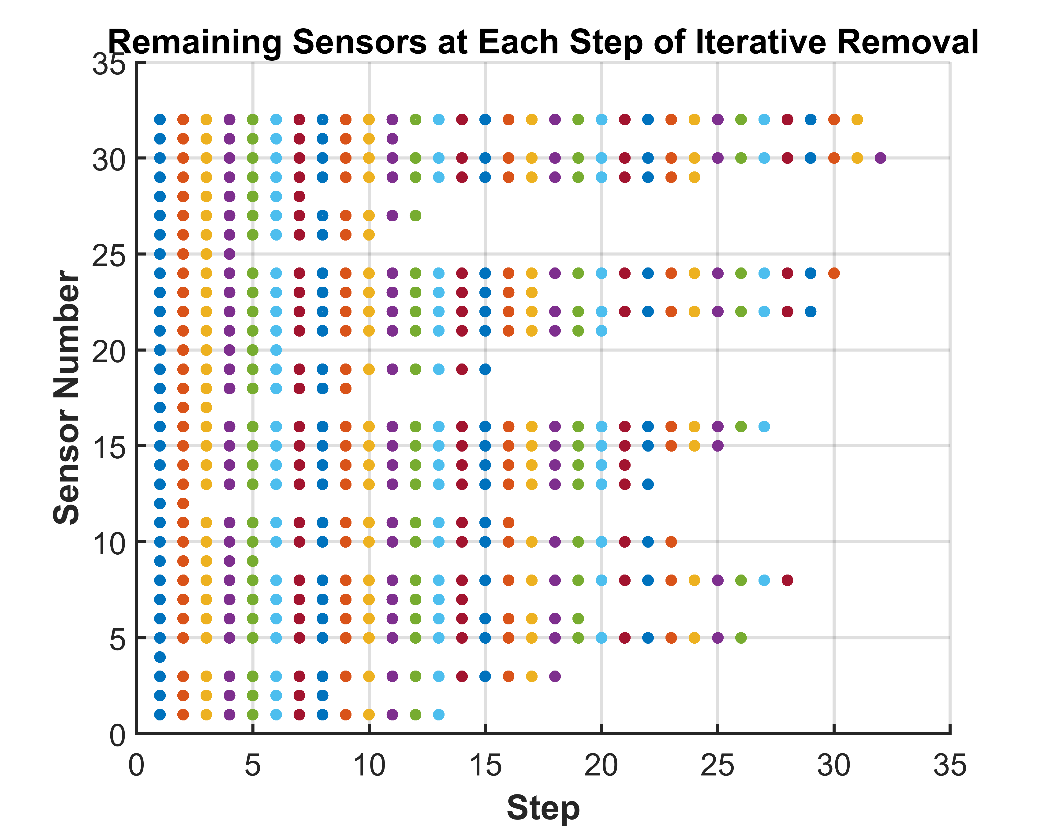


**Figure S28**. Graphical representation of **Table S8**.

**Table S9**. Sensors retained at each iteration of the sensor-elimination procedure for PMI: 2 days vs. PMI: 3 days classification (Classifier A3).

| **Step** | **No. of Sensors** | **Remaining Sensors at Each Step of Iterative Removal** |
| --- | --- | --- |
| 1 | 32 | 1 2 3 4 5 6 7 8 9 10 11 12 13 14 15 16 17 18 19 20 21 22 23 24 25 26 27 28 29 30 31 32 |
| 2 | 31 | 1 2 3 5 6 7 8 9 10 11 12 13 14 15 16 17 18 19 20 21 22 23 24 25 26 27 28 29 30 31 32 |
| 3 | 30 | 1 2 3 5 6 7 8 9 10 11 13 14 15 16 17 18 19 20 21 22 23 24 25 26 27 28 29 30 31 32 |
| 4 | 29 | 1 2 3 5 6 7 8 9 10 11 13 14 15 16 18 19 20 21 22 23 24 25 26 27 28 29 30 31 32 |
| 5 | 28 | 1 2 3 5 6 7 8 10 11 13 14 15 16 18 19 20 21 22 23 24 25 26 27 28 29 30 31 32 |
| 6 | 27 | 1 2 3 5 6 7 8 10 11 13 14 15 16 18 19 20 21 22 23 24 26 27 28 29 30 31 32 |
| 7 | 26 | 1 2 3 5 6 7 8 10 11 13 14 15 16 18 19 21 22 23 24 26 27 28 29 30 31 32 |
| 8 | 25 | 1 3 5 6 7 8 10 11 13 14 15 16 18 19 21 22 23 24 26 27 28 29 30 31 32 |
| 9 | 24 | 3 5 6 7 8 10 11 13 14 15 16 18 19 21 22 23 24 26 27 28 29 30 31 32 |
| 10 | 23 | 3 5 6 7 8 10 11 13 14 15 16 19 21 22 23 24 26 27 28 29 30 31 32 |
| 11 | 22 | 3 5 6 7 8 10 11 13 14 15 16 19 21 22 23 24 26 27 29 30 31 32 |
| 12 | 21 | 3 5 6 7 8 10 11 13 14 15 16 19 21 22 23 24 26 29 30 31 32 |
| 13 | 20 | 3 5 6 7 8 10 11 13 14 15 16 21 22 23 24 26 29 30 31 32 |
| 14 | 19 | 3 5 6 7 8 10 13 14 15 16 21 22 23 24 26 29 30 31 32 |
| 15 | 18 | 3 5 6 7 8 10 13 14 15 16 21 22 23 24 26 29 30 32 |
| 16 | 17 | 3 5 6 7 8 10 13 14 15 16 21 22 23 24 29 30 32 |
| 17 | 16 | 5 6 7 8 10 13 14 15 16 21 22 23 24 29 30 32 |
| 18 | 15 | 5 6 8 10 13 14 15 16 21 22 23 24 29 30 32 |
| 19 | 14 | 5 6 8 10 13 14 15 16 21 22 24 29 30 32 |
| 20 | 13 | 5 6 8 10 13 14 15 16 22 24 29 30 32 |
| 21 | 12 | 5 8 10 13 14 15 16 22 24 29 30 32 |
| 22 | 11 | 5 8 10 14 15 16 22 24 29 30 32 |
| 23 | 10 | 5 8 10 15 16 22 24 29 30 32 |
| 24 | 9 | 5 8 15 16 22 24 29 30 32 |
| 25 | 8 | 5 8 15 16 22 24 30 32 |
| 26 | 7 | 8 15 16 22 24 30 32 |
| 27 | 6 | 8 15 16 24 30 32 |
| 28 | 5 | 8 16 24 30 32 |
| 29 | 4 | 8 24 30 32 |
| 30 | 3 | 24 30 32 |
| 31 | 2 | 30 32 |
| 32 | 1 | 32 |


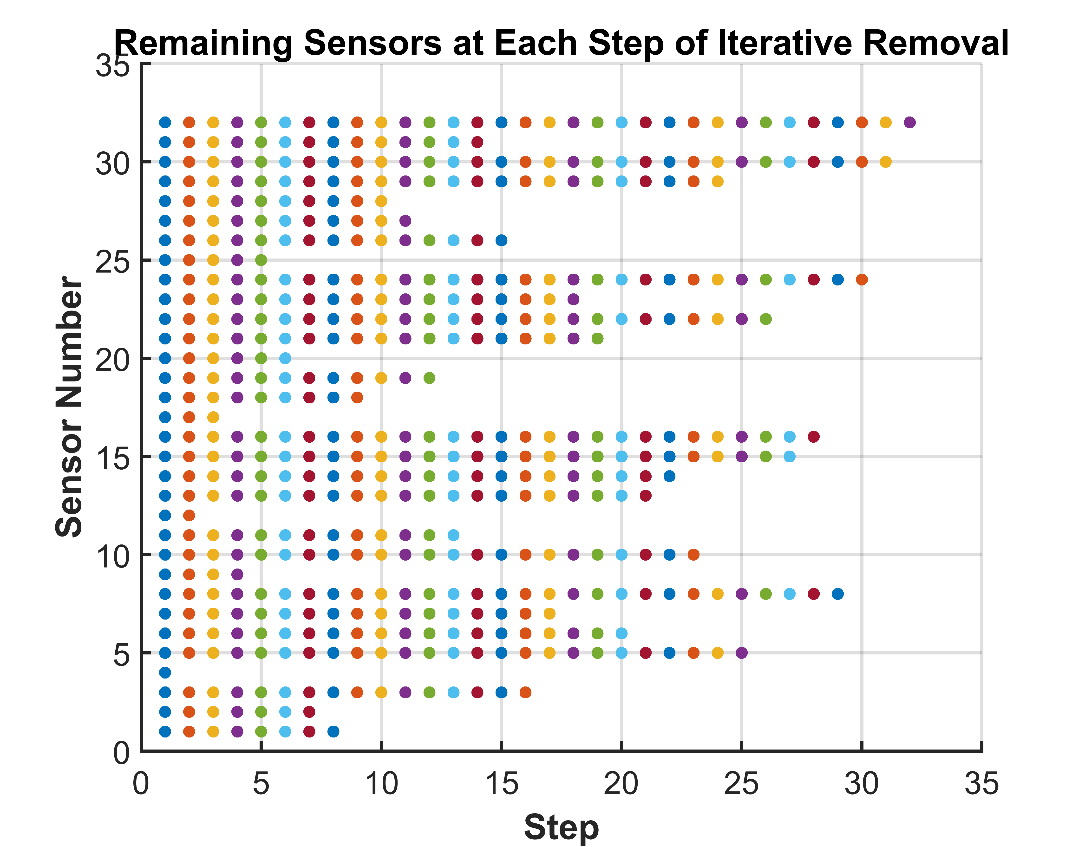


**Figure S29**. Graphical representation of **Table S9**.

**Table S10**. Evolution of validation and test performance metrics over 32 steps of the iterative sensor-elimination procedure for PMI: 1 day vs. PMI: 2–3 days classification (Classifier A2). Bold red: step selected.

| **Step** | **Train Accuracy** | **Train Sensitivity** | **Train Specificity** | **Test Accuracy** | **Test Sensitivity** | **Test Specificity** |
| --- | --- | --- | --- | --- | --- | --- |
| 1 | 0.95285 | 0.9057 | 0.97567 | 0.94859 | 0.90102 | 0.97167 |
| **2** | **0.95142** | **0.90268** | **0.97502** | **0.96233** | **0.92913** | **0.97840** |
| 3 | 0.94037 | 0.88317 | 0.96807 | 0.95668 | 0.88889 | 0.98950 |
| 4 | 0.94705 | 0.89411 | 0.9727 | 0.95883 | 0.91854 | 0.97829 |
| 5 | 0.95053 | 0.90355 | 0.97329 | 0.96209 | 0.94477 | 0.97046 |
| 6 | 0.76370 | 0.41419 | 0.93293 | 0.72960 | 0.35241 | 0.91241 |
| 7 | 0.75346 | 0.41383 | 0.91790 | 0.78776 | 0.48750 | 0.93333 |
| 8 | 0.76023 | 0.42233 | 0.92388 | 0.77495 | 0.47557 | 0.91969 |
| 9 | 0.91024 | 0.82198 | 0.95298 | 0.93805 | 0.88475 | 0.96388 |
| 10 | 0.90582 | 0.80605 | 0.95416 | 0.91119 | 0.79433 | 0.96752 |
| 11 | 0.89859 | 0.78038 | 0.95586 | 0.93366 | 0.86667 | 0.96601 |
| 12 | 0.90921 | 0.81161 | 0.95647 | 0.91530 | 0.84884 | 0.94747 |
| 13 | 0.85072 | 0.73487 | 0.90682 | 0.85146 | 0.76829 | 0.89173 |
| 14 | 0.90879 | 0.81559 | 0.95395 | 0.92179 | 0.81974 | 0.97101 |
| 15 | 0.88032 | 0.72855 | 0.95383 | 0.87758 | 0.72851 | 0.94967 |
| 16 | 0.89686 | 0.78268 | 0.95215 | 0.90938 | 0.78947 | 0.96752 |
| 17 | 0.88856 | 0.77088 | 0.94558 | 0.90879 | 0.83163 | 0.94595 |
| 18 | 0.78350 | 0.52017 | 0.91105 | 0.79469 | 0.55978 | 0.90814 |
| 19 | 0.90318 | 0.81226 | 0.9472 | 0.91651 | 0.84302 | 0.95211 |
| 20 | 0.78440 | 0.49653 | 0.92393 | 0.78571 | 0.45283 | 0.94562 |
| 21 | 0.86739 | 0.69752 | 0.94969 | 0.87611 | 0.74830 | 0.93770 |
| 22 | 0.77444 | 0.46880 | 0.92247 | 0.78986 | 0.50370 | 0.92832 |
| 23 | 0.74831 | 0.39024 | 0.92170 | 0.77454 | 0.40650 | 0.95276 |
| 24 | 0.89031 | 0.78335 | 0.94215 | 0.89971 | 0.81818 | 0.93886 |
| 25 | 0.91234 | 0.81941 | 0.95735 | 0.91030 | 0.75510 | 0.98522 |
| 26 | 0.89141 | 0.77548 | 0.94753 | 0.90494 | 0.74419 | 0.98305 |
| 27 | 0.89784 | 0.77744 | 0.95624 | 0.91593 | 0.84932 | 0.94771 |
| 28 | 0.78374 | 0.47292 | 0.93438 | 0.83511 | 0.52459 | 0.98425 |
| 29 | 0.91532 | 0.82844 | 0.95738 | 0.96000 | 0.97959 | 0.95050 |
| 30 | 0.79371 | 0.48795 | 0.94169 | 0.82301 | 0.51351 | 0.97368 |
| 31 | 0.83211 | 0.62613 | 0.93217 | 0.80000 | 0.45833 | 0.96078 |
| 32 | 0.72941 | 0.47748 | 0.85153 | 0.78378 | 0.50000 | 0.92000 |

**Figure S30**. Graphical representation of Table S10.

**Table S11**. Evolution of validation and test performance metrics over 32 steps of the iterative sensor-elimination procedure for PMI: 2 days vs. PMI: 3 days classification (Classifier A3). Bold red: step selected.

| **Step** | **Train Accuracy** | **Train Sensitivity** | **Train Specificity** | **Test Accuracy** | **Test Sensitivity** | **Test Specificity** |
| --- | --- | --- | --- | --- | --- | --- |
| 1 | 0.69286 | 0.66629 | 0.71822 | 0.72783 | 0.72222 | 0.73317 |
| 2 | 0.69310 | 0.66040 | 0.72429 | 0.66709 | 0.63021 | 0.70223 |
| 3 | 0.69627 | 0.66876 | 0.72251 | 0.72441 | 0.69892 | 0.74872 |
| **4** | **0.94796** | **0.94625** | **0.94960** | **0.95652** | **0.94429** | **0.96817** |
| 5 | 0.94860 | 0.94464 | 0.95238 | 0.95781 | 0.94524 | 0.96978 |
| 6 | 0.92564 | 0.92236 | 0.92877 | 0.94307 | 0.95210 | 0.93447 |
| 7 | 0.94532 | 0.93970 | 0.95069 | 0.94394 | 0.95342 | 0.93491 |
| 8 | 0.70796 | 0.68387 | 0.73094 | 0.74173 | 0.71935 | 0.76308 |
| 9 | 0.71205 | 0.68346 | 0.73932 | 0.75041 | 0.72054 | 0.77885 |
| 10 | 0.71377 | 0.69809 | 0.72873 | 0.75514 | 0.72982 | 0.77926 |
| 11 | 0.71451 | 0.68689 | 0.74087 | 0.70251 | 0.67279 | 0.73077 |
| 12 | 0.90606 | 0.90444 | 0.90761 | 0.91745 | 0.90385 | 0.93040 |
| 13 | 0.90398 | 0.89651 | 0.91111 | 0.94685 | 0.94758 | 0.94615 |
| 14 | 0.71570 | 0.69118 | 0.73909 | 0.71577 | 0.66809 | 0.76113 |
| 15 | 0.89842 | 0.89298 | 0.90361 | 0.91685 | 0.90583 | 0.92735 |
| 16 | 0.89709 | 0.89410 | 0.89995 | 0.91183 | 0.91905 | 0.90498 |
| 17 | 0.72307 | 0.69933 | 0.74573 | 0.81034 | 0.78283 | 0.83654 |
| 18 | 0.89910 | 0.89546 | 0.90256 | 0.92388 | 0.89247 | 0.95385 |
| 19 | 0.75226 | 0.73001 | 0.77350 | 0.77746 | 0.76301 | 0.79121 |
| 20 | 0.74596 | 0.73053 | 0.76068 | 0.75455 | 0.72050 | 0.78698 |
| 21 | 0.72886 | 0.70672 | 0.75000 | 0.78618 | 0.76351 | 0.80769 |
| 22 | 0.73837 | 0.72638 | 0.74981 | 0.72043 | 0.72794 | 0.71329 |
| 23 | 0.72266 | 0.71864 | 0.72650 | 0.74803 | 0.75000 | 0.74615 |
| 24 | 0.74976 | 0.73831 | 0.76068 | 0.74123 | 0.74775 | 0.73504 |
| 25 | 0.77857 | 0.76932 | 0.78739 | 0.74877 | 0.75758 | 0.74038 |
| 26 | 0.79700 | 0.79028 | 0.80342 | 0.79096 | 0.86047 | 0.72527 |
| 27 | 0.88776 | 0.87910 | 0.89601 | 0.92105 | 0.90541 | 0.93590 |
| 28 | 0.90376 | 0.90681 | 0.90085 | 0.92126 | 0.91935 | 0.92308 |
| 29 | 0.74208 | 0.75391 | 0.73077 | 0.76238 | 0.77551 | 0.75000 |
| 30 | 0.89504 | 0.91642 | 0.87464 | 0.92105 | 0.91892 | 0.92308 |
| 31 | 0.81659 | 0.83036 | 0.80342 | 0.80000 | 0.66667 | 0.92308 |
| 32 | 0.73799 | 0.75000 | 0.72650 | 0.76000 | 0.75000 | 0.76923 |

**Figure S31**. Graphical representation of Table **S11**.


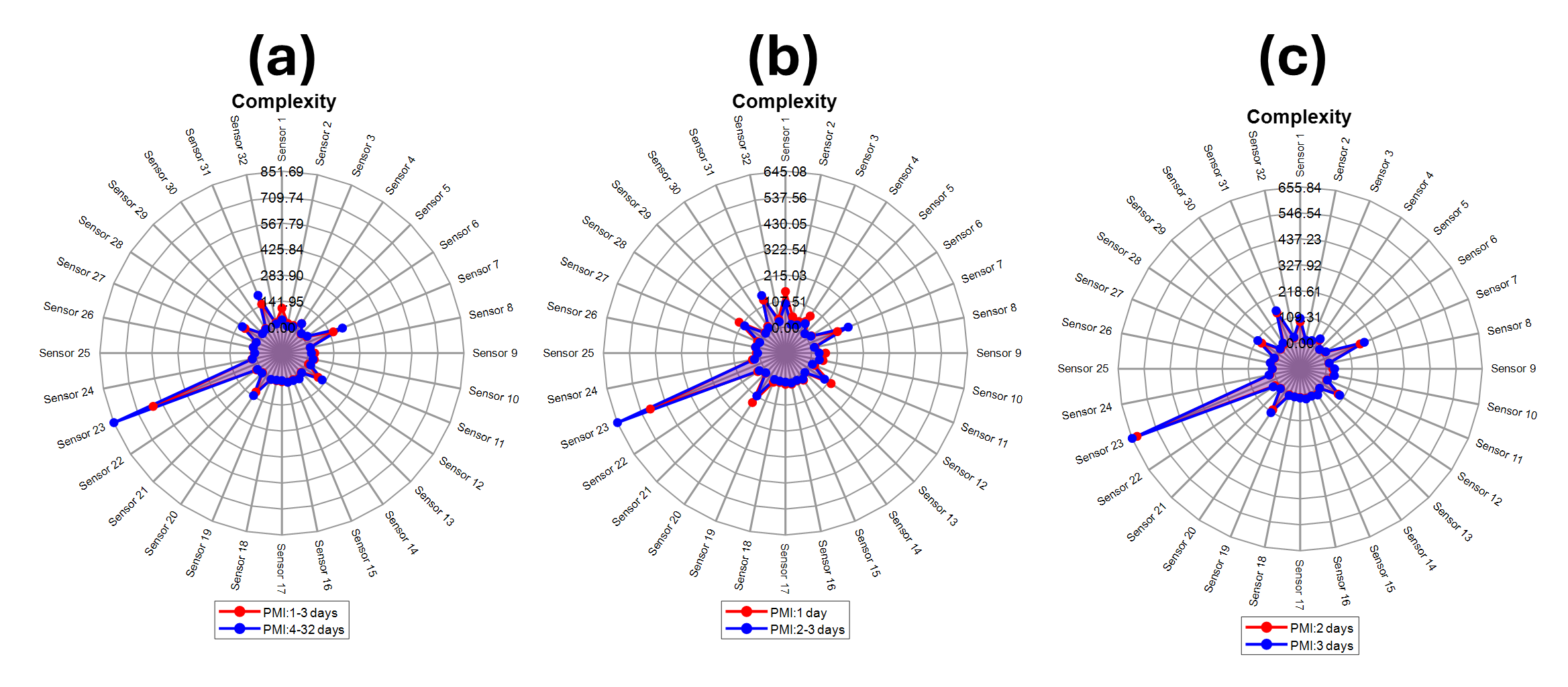


**Figure S32.** Radar plots of *complexity* for Classifier (a) A1, (b) A2, and (c) A3, highlighting its contribution to model performance.

**Table S12**. Optimized hyperparameters for three Optimizable Ensemble classifiers distinguishing PMIs.

| Classifier | Method | Number of Learning Cycles | Learning Rate | Minimum Leaf Size |
| --- | --- | --- | --- | --- |
| A1 | GentleBoost | 494 | 0.0147760 | 2 |
| A2 | AdaBoostM1 | 497 | 0.9659500 | 39 |
| A3 | GentleBoost | 495 | 0.0014714 | 2 |


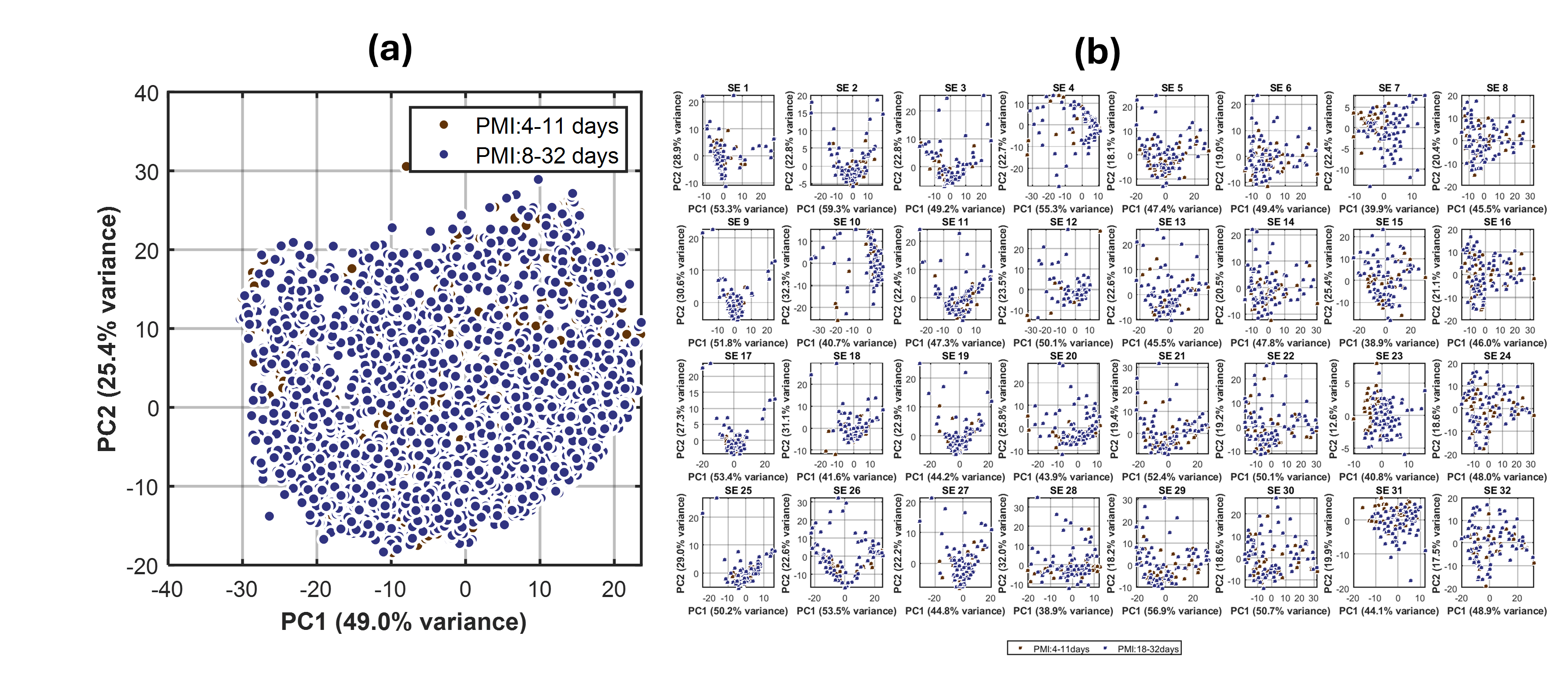


**Figure S33**. A) PCA plot of the full dataset, showing poor class separation (Classifier A4). B) Individual PCA plots for all 32 sensors, highlighting the contribution of each sensor to class differentiation.


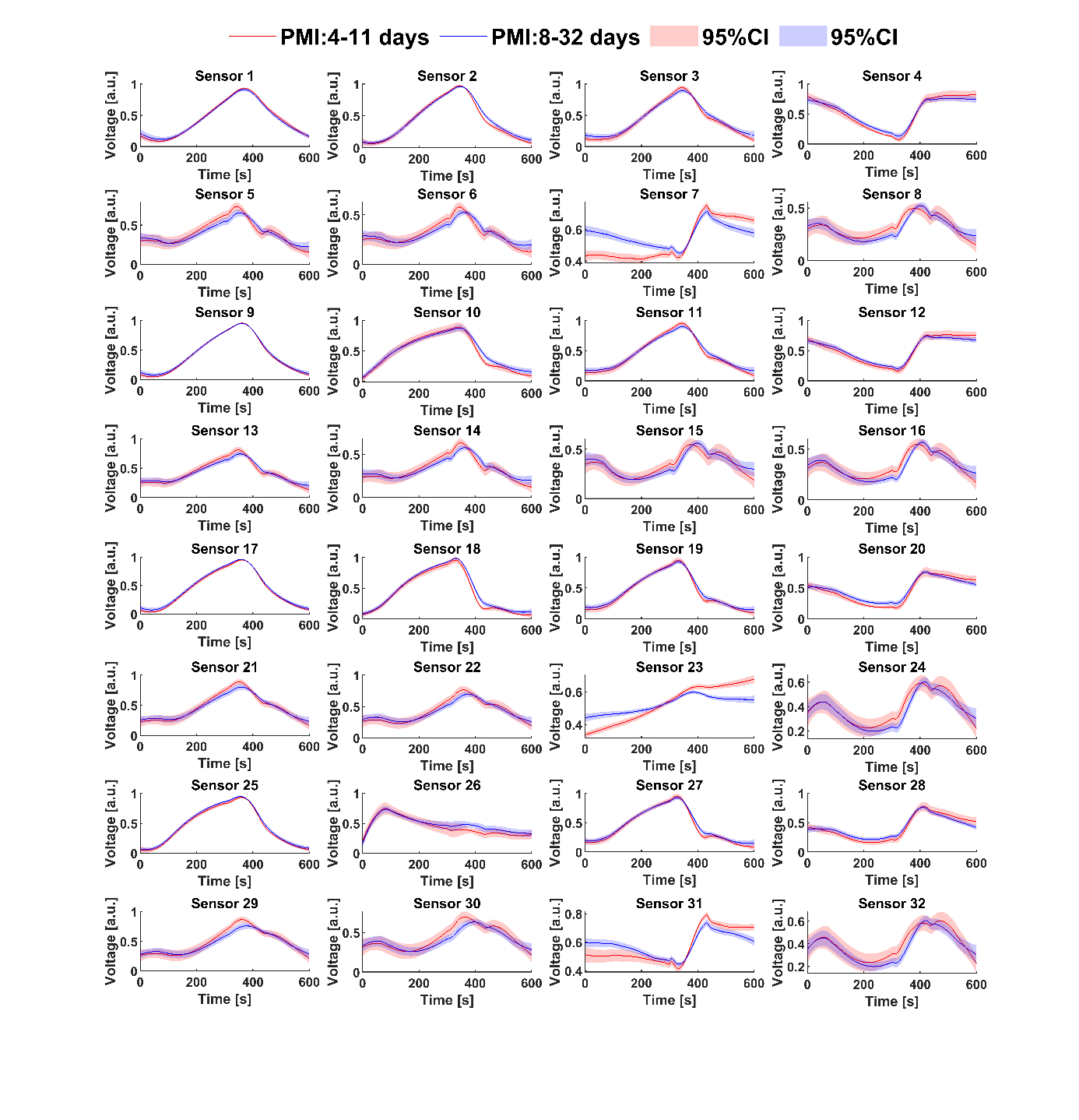


**Figure S34**. Multi-sensor response patterns of the *e*-nose to VOCs from animal samples at different PMIs (Classifier A4). The panels show normalized voltage signals with 95% Cis for 32 MOS sensors. Red: 4–11 days; Blue: 8–32 days PMIs. Distinct response patterns highlight the e-nose ability to differentiate between classes based on their respective VOC profiles.


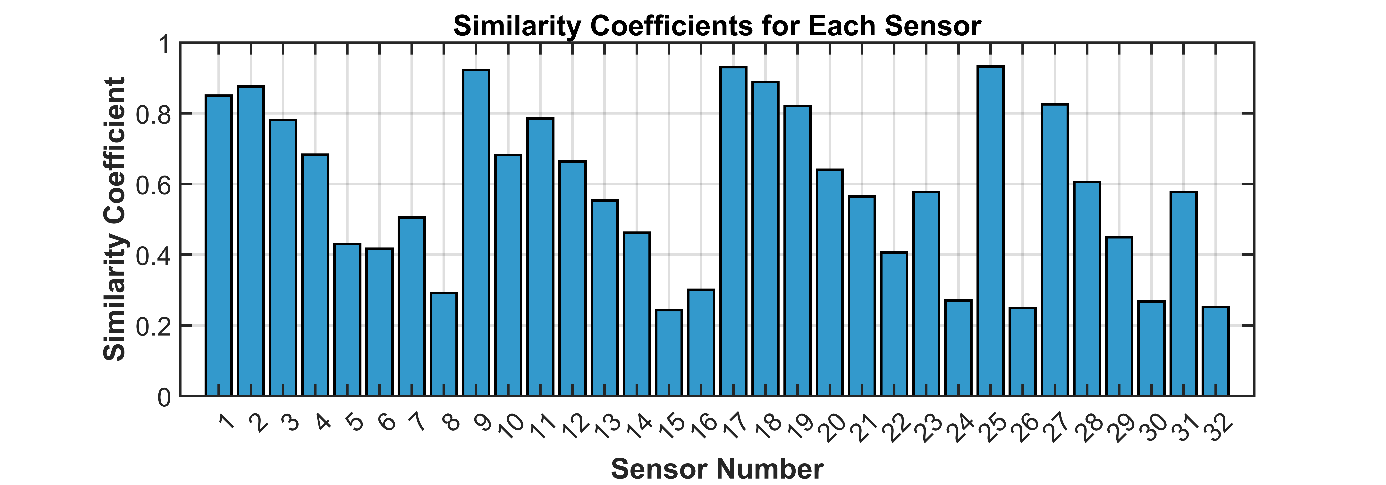


**Figure S35.** Similarity coefficients across the 32-sensor array discriminating animal samples at different PMIs (Classifier A4). Bars denote the mean Pearson correlations between all inter-class signal pairs for each sensor. Lower similarity coefficients indicate greater discriminatory power between classes.

**Table S13**. Sensors retained at each iteration of the sensor-elimination procedure for PMI: 4–11 days vs. PMI: 8–32 days classification (Classifier A4).

| **Step** | **No. of Sensors** | **Remaining Sensors at Each Step of Iterative Removal** |
| --- | --- | --- |
| 1 | 32 | 1 2 3 4 5 6 7 8 9 10 11 12 13 14 15 16 17 18 19 20 21 22 23 24 25 26 27 28 29 30 31 32 |
| 2 | 31 | 1 2 3 4 5 6 7 8 9 10 11 12 13 14 15 16 17 18 19 20 21 22 23 24 26 27 28 29 30 31 32 |
| 3 | 30 | 1 2 3 4 5 6 7 8 9 10 11 12 13 14 15 16 18 19 20 21 22 23 24 26 27 28 29 30 31 32 |
| 4 | 29 | 1 2 3 4 5 6 7 8 10 11 12 13 14 15 16 18 19 20 21 22 23 24 26 27 28 29 30 31 32 |
| 5 | 28 | 1 2 3 4 5 6 7 8 10 11 12 13 14 15 16 19 20 21 22 23 24 26 27 28 29 30 31 32 |
| 6 | 27 | 1 3 4 5 6 7 8 10 11 12 13 14 15 16 19 20 21 22 23 24 26 27 28 29 30 31 32 |
| 7 | 26 | 3 4 5 6 7 8 10 11 12 13 14 15 16 19 20 21 22 23 24 26 27 28 29 30 31 32 |
| 8 | 25 | 3 4 5 6 7 8 10 11 12 13 14 15 16 19 20 21 22 23 24 26 28 29 30 31 32 |
| 9 | 24 | 3 4 5 6 7 8 10 11 12 13 14 15 16 20 21 22 23 24 26 28 29 30 31 32 |
| 10 | 23 | 3 4 5 6 7 8 10 12 13 14 15 16 20 21 22 23 24 26 28 29 30 31 32 |
| 11 | 22 | 4 5 6 7 8 10 12 13 14 15 16 20 21 22 23 24 26 28 29 30 31 32 |
| 12 | 21 | 5 6 7 8 10 12 13 14 15 16 20 21 22 23 24 26 28 29 30 31 32 |
| 13 | 20 | 5 6 7 8 12 13 14 15 16 20 21 22 23 24 26 28 29 30 31 32 |
| 14 | 19 | 5 6 7 8 13 14 15 16 20 21 22 23 24 26 28 29 30 31 32 |
| 15 | 18 | 5 6 7 8 13 14 15 16 21 22 23 24 26 28 29 30 31 32 |
| 16 | 17 | 5 6 7 8 13 14 15 16 21 22 23 24 26 29 30 31 32 |
| 17 | 16 | 5 6 7 8 13 14 15 16 21 22 23 24 26 29 30 32 |
| 18 | 15 | 5 6 7 8 13 14 15 16 21 22 24 26 29 30 32 |
| 19 | 14 | 5 6 7 8 13 14 15 16 22 24 26 29 30 32 |
| 20 | 13 | 5 6 7 8 14 15 16 22 24 26 29 30 32 |
| 21 | 12 | 5 6 8 14 15 16 22 24 26 29 30 32 |
| 22 | 11 | 5 6 8 15 16 22 24 26 29 30 32 |
| 23 | 10 | 5 6 8 15 16 22 24 26 30 32 |
| 24 | 9 | 6 8 15 16 22 24 26 30 32 |
| 25 | 8 | 8 15 16 22 24 26 30 32 |
| 26 | 7 | 8 15 16 24 26 30 32 |
| 27 | 6 | 8 15 24 26 30 32 |
| 28 | 5 | 15 24 26 30 32 |
| 29 | 4 | 15 26 30 32 |
| 30 | 3 | 15 26 32 |
| 31 | 2 | 15 26 |
| 32 | 1 | 15 |


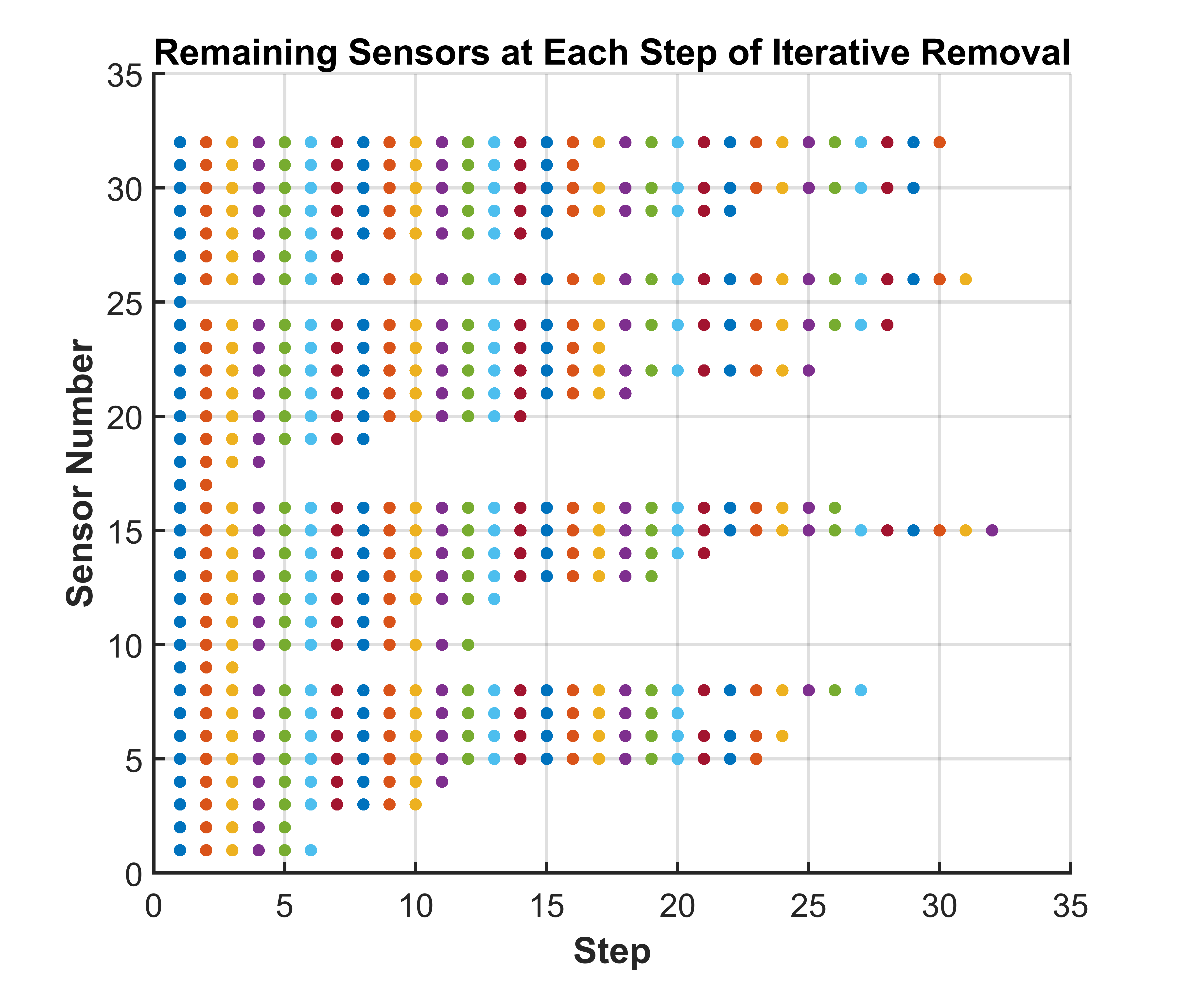


**Figure S36**. Graphical representation of **Table S13.**

**Table S14**. Evolution of validation and test performance metrics over 32 steps of the iterative sensor-elimination procedure for PMI: 4-11 days vs. PMI: 8-32 days classification (Classifier A4). Bold red: step selected.

| **Step** | **Train Accuracy** | **Train Sensitivity** | **Train Specificity** | **Test Accuracy** | **Test Sensitivity** | **Test Specificity** |
| --- | --- | --- | --- | --- | --- | --- |
| 1 | 0.77604 | 0.46272 | 0.91434 | 0.77746 | 0.44037 | 0.92683 |
| 2 | 0.75944 | 0.40991 | 0.91387 | 0.79651 | 0.50476 | 0.92469 |
| 3 | 0.76577 | 0.45752 | 0.90188 | 0.83784 | 0.59804 | 0.94372 |
| **4** | **0.94410** | **0.84685** | **0.98706** | **0.96885** | **0.93878** | **0.98206** |
| 5 | 0.77698 | 0.43174 | 0.92942 | 0.76452 | 0.44211 | 0.90698 |
| 6 | 0.76612 | 0.35714 | 0.94658 | 0.76254 | 0.34783 | 0.94686 |
| 7 | 0.77945 | 0.52764 | 0.89068 | 0.76042 | 0.53409 | 0.86000 |
| 8 | 0.77462 | 0.49673 | 0.89729 | 0.79061 | 0.49412 | 0.92188 |
| 9 | 0.76647 | 0.44687 | 0.90745 | 0.78947 | 0.43902 | 0.94565 |
| 10 | 0.79852 | 0.51562 | 0.92346 | 0.81569 | 0.62821 | 0.89831 |
| 11 | 0.80846 | 0.56825 | 0.9147 | 0.83197 | 0.56757 | 0.94706 |
| 12 | 0.79743 | 0.53271 | 0.91415 | 0.79828 | 0.51389 | 0.92547 |
| 13 | 0.93293 | 0.82516 | 0.98052 | 0.95946 | 0.88235 | 0.99351 |
| 14 | 0.9326 | 0.81100 | 0.98633 | 0.94286 | 0.81250 | 1.00000 |
| 15 | 0.93274 | 0.82577 | 0.97997 | 0.94975 | 0.86885 | 0.98551 |
| 16 | 0.79282 | 0.66411 | 0.84975 | 0.80851 | 0.61404 | 0.89313 |
| 17 | 0.91370 | 0.80000 | 0.96393 | 0.9322 | 0.83333 | 0.97561 |
| 18 | 0.91127 | 0.76471 | 0.97596 | 0.92771 | 0.78431 | 0.99130 |
| 19 | 0.89278 | 0.73364 | 0.96292 | 0.88387 | 0.72917 | 0.95327 |
| 20 | 0.88222 | 0.69849 | 0.96337 | 0.90278 | 0.79545 | 0.95000 |
| 21 | 0.89158 | 0.73569 | 0.96034 | 0.91729 | 0.87805 | 0.93478 |
| 22 | 0.95814 | 0.89614 | 0.98556 | 0.95902 | 0.91892 | 0.97647 |
| 23 | 0.93093 | 0.86601 | 0.95960 | 0.97297 | 0.94118 | 0.98701 |
| 24 | 0.97222 | 0.92391 | 0.99359 | 0.95960 | 0.90000 | 0.98551 |
| 25 | 0.97000 | 0.93061 | 0.98739 | 1.00000 | 1.00000 | 1.00000 |
| 26 | 0.98143 | 0.95349 | 0.99381 | 1.00000 | 1.00000 | 1.00000 |
| 27 | 0.99000 | 0.97826 | 0.99519 | 1.00000 | 1.00000 | 1.00000 |
| 28 | 0.97000 | 0.92157 | 0.99135 | 0.94545 | 0.88235 | 0.97368 |
| 29 | 0.84000 | 0.61475 | 0.93885 | 0.84091 | 0.71429 | 0.90000 |
| 30 | 0.85000 | 0.67391 | 0.92788 | 0.75758 | 0.40000 | 0.91304 |
| 31 | 0.73000 | 0.40984 | 0.87050 | 0.77273 | 0.42857 | 0.93333 |
| 32 | 0.73000 | 0.41935 | 0.86957 | 0.63636 | 0.33333 | 0.75000 |

**Figure S37**. Graphical representation of **Table S14**.


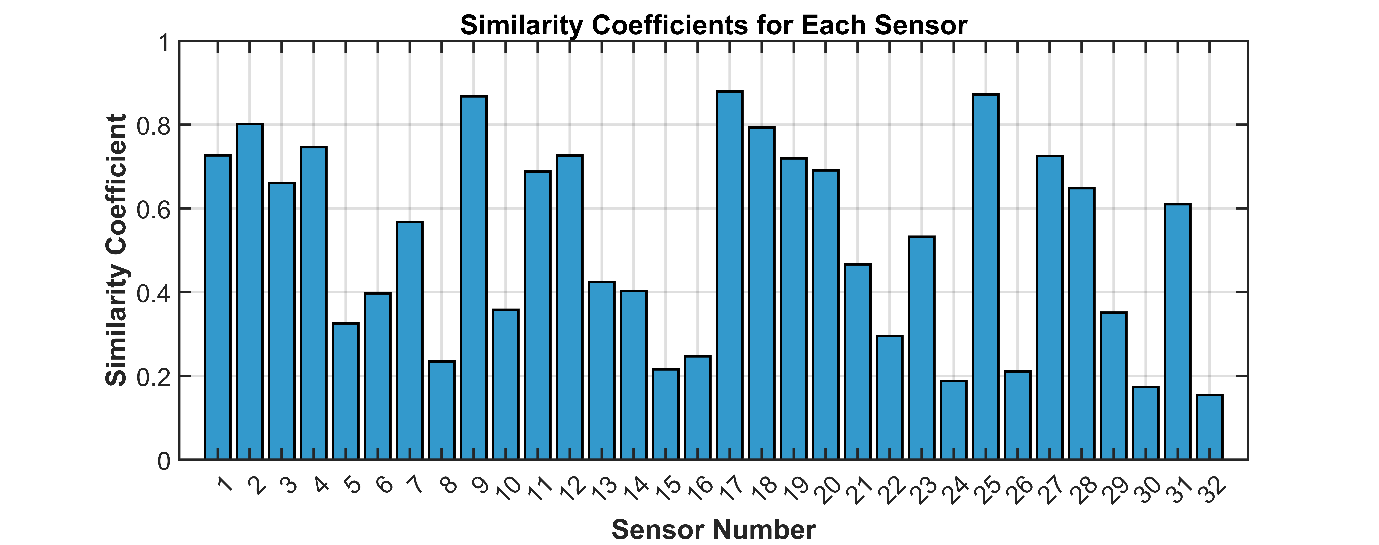


**Figure S38.** Similarity coefficients across the 32-sensor array discriminating animal samples at different PMIs (Classifier A1). Bars denote the mean Pearson correlations between all inter-class signal pairs for each sensor. Lower similarity coefficients indicate greater discriminatory power between classes. The sensor utility ranking algorithm was applied exclusively to the training dataset to optimize sensor selection.


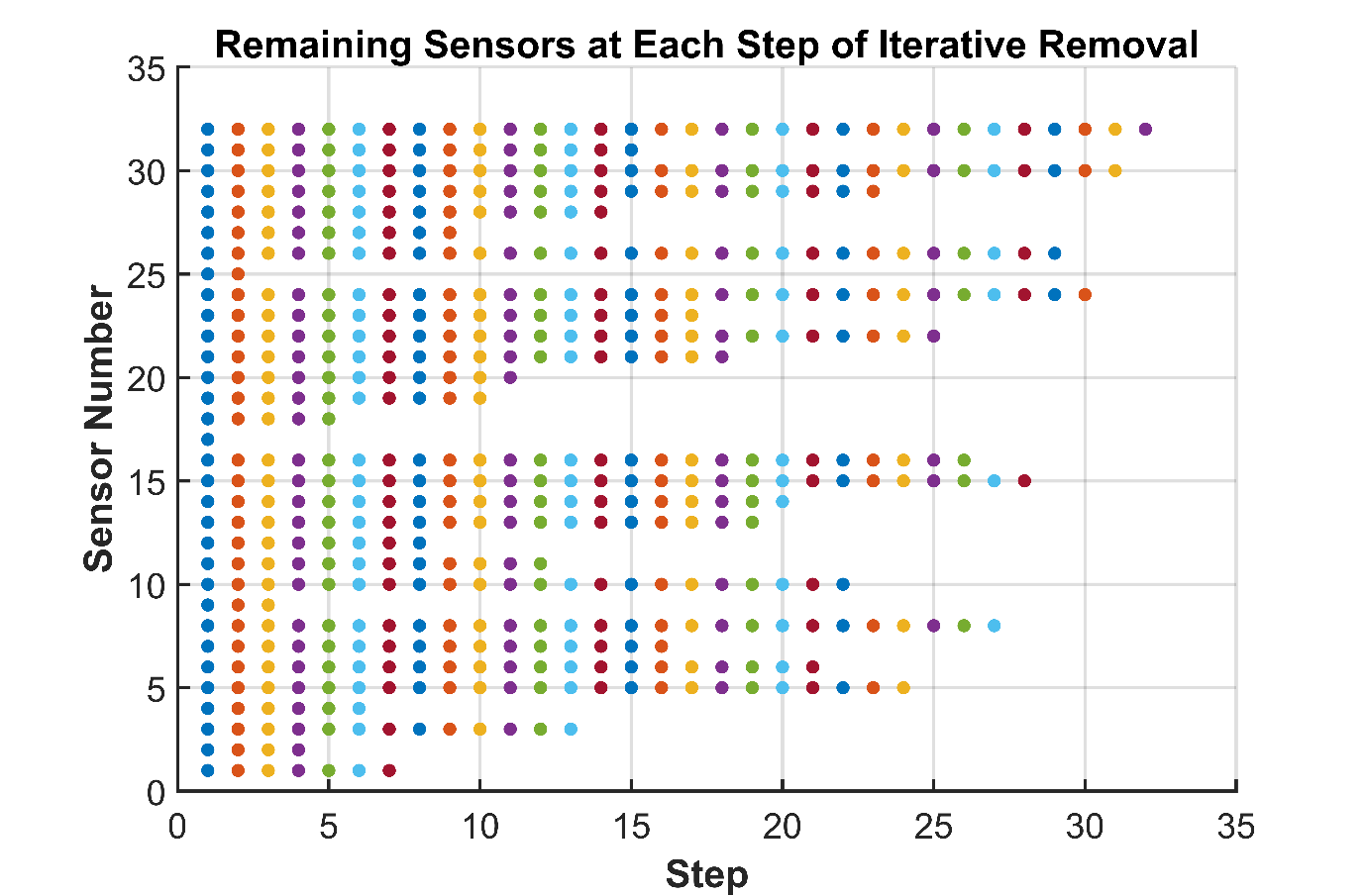


**Figure S39**. Graphical representation of sensor retention across removal steps for PMI: 1–3 days vs. PMI: 4–32 days discrimination (Classifier A1).


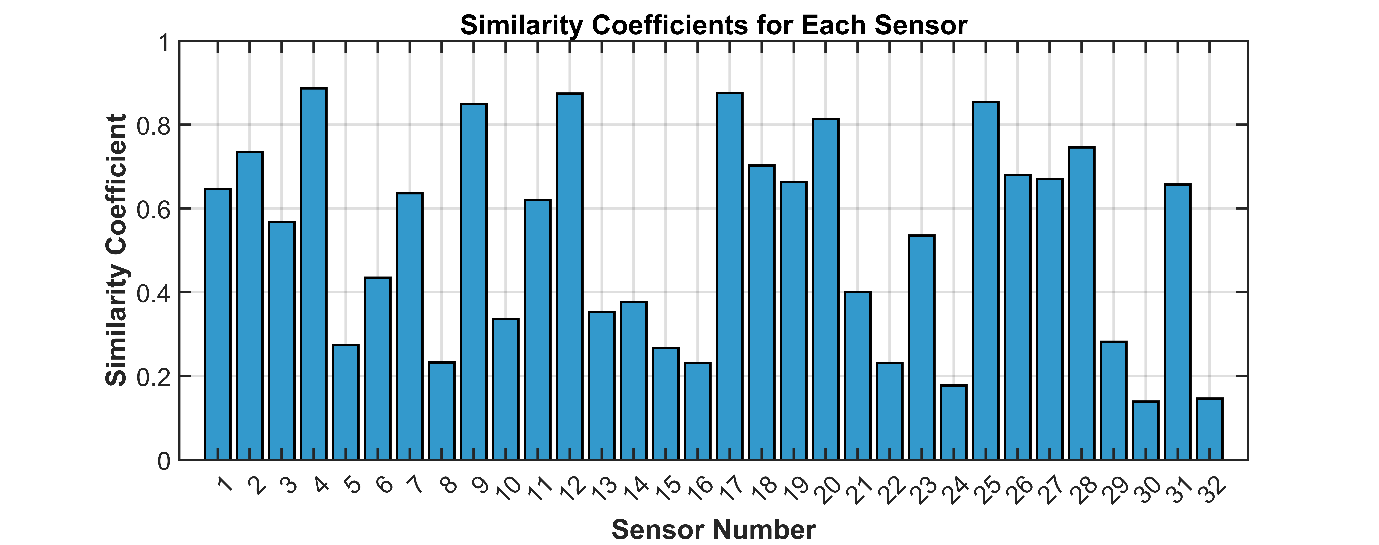


**Figure S40.** Similarity coefficients across the 32-sensor array discriminating animal samples at different PMIs (Classifier A2). Bars denote the mean Pearson correlations between all inter-class signal pairs for each sensor. Lower similarity coefficients indicate greater discriminatory power between classes. The sensor utility ranking algorithm was applied exclusively to the training dataset to optimize sensor selection.


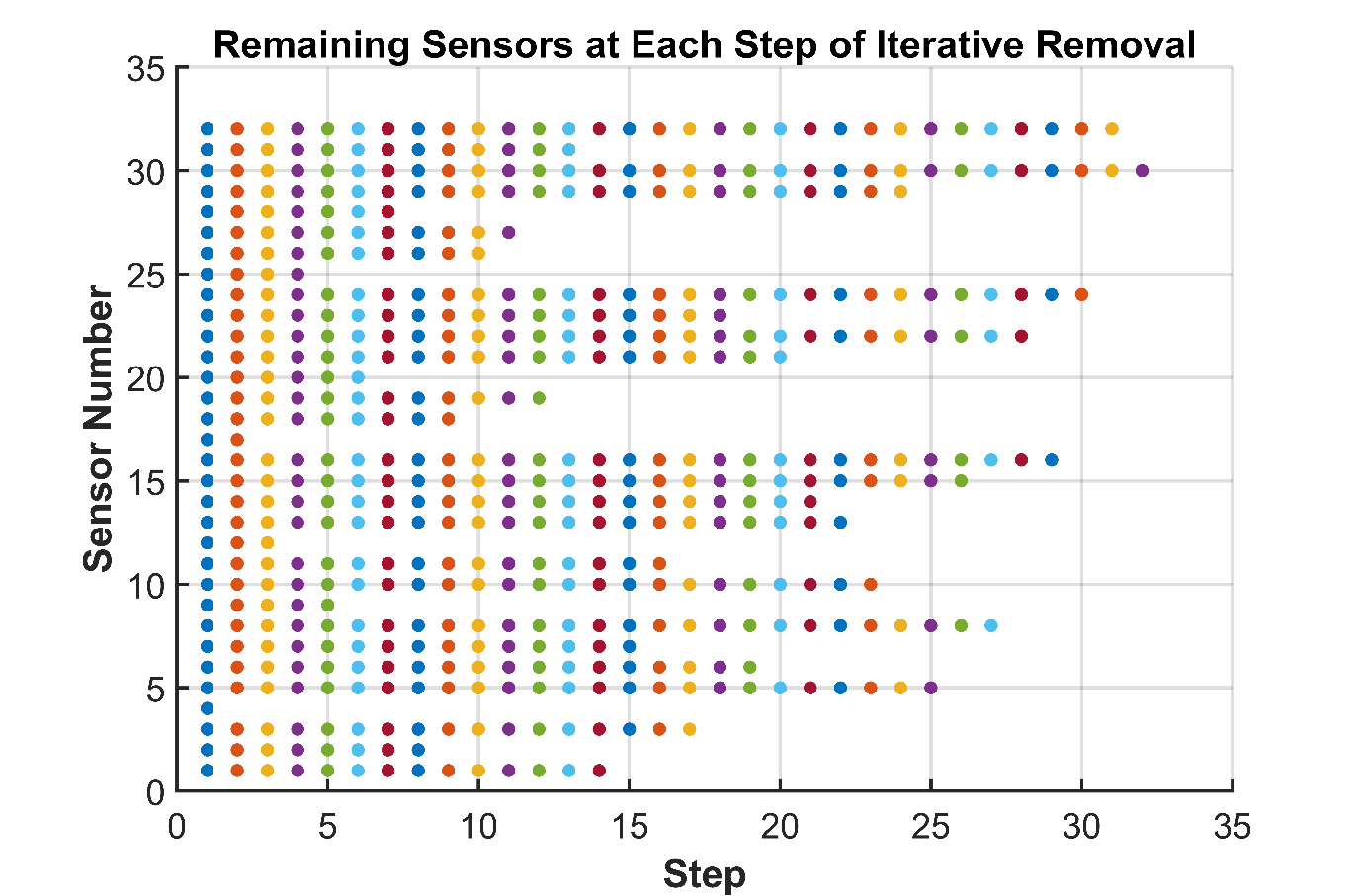


**Figure S41**. Graphical representation of sensor retention across removal steps for PMI: 1 day vs. PMI: 2–3 days discrimination (Classifier A2). The sensor utility ranking algorithm was applied exclusively to the training dataset to optimize sensor selection.


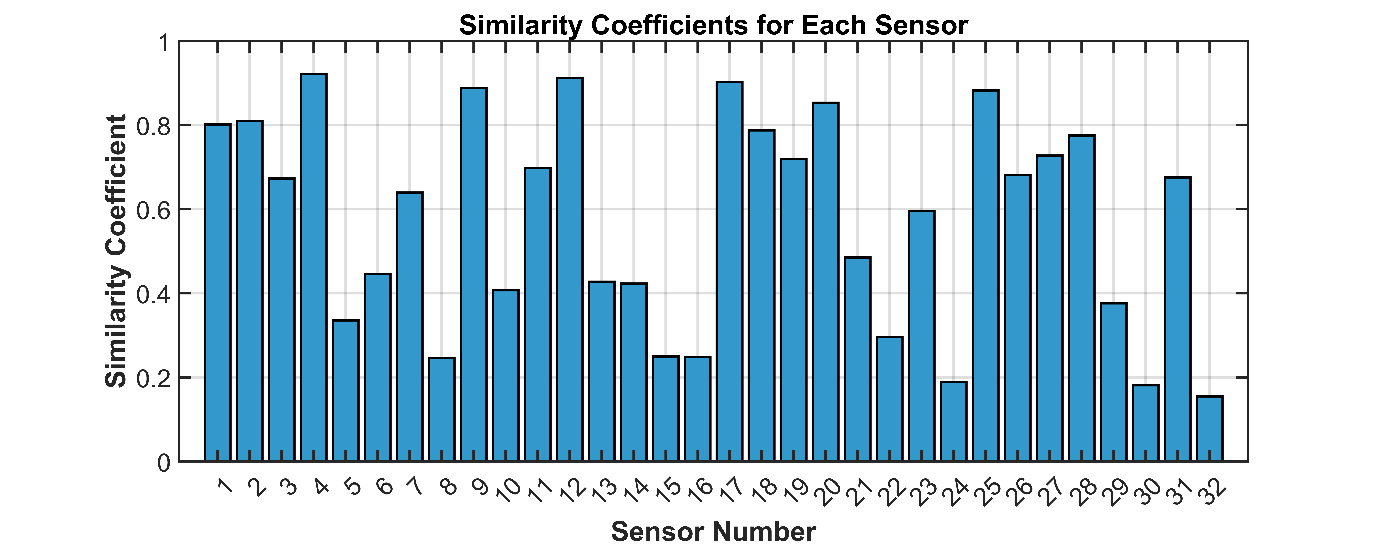


**Figure S42.** Similarity coefficients across the 32-sensor array discriminating animal samples at different PMIs (Classifier A3). Bars denote the mean Pearson correlations between all inter-class signal pairs for each sensor. Lower similarity coefficients indicate greater discriminatory power between classes. The sensor utility ranking algorithm was applied exclusively to the training dataset to optimize sensor selection.


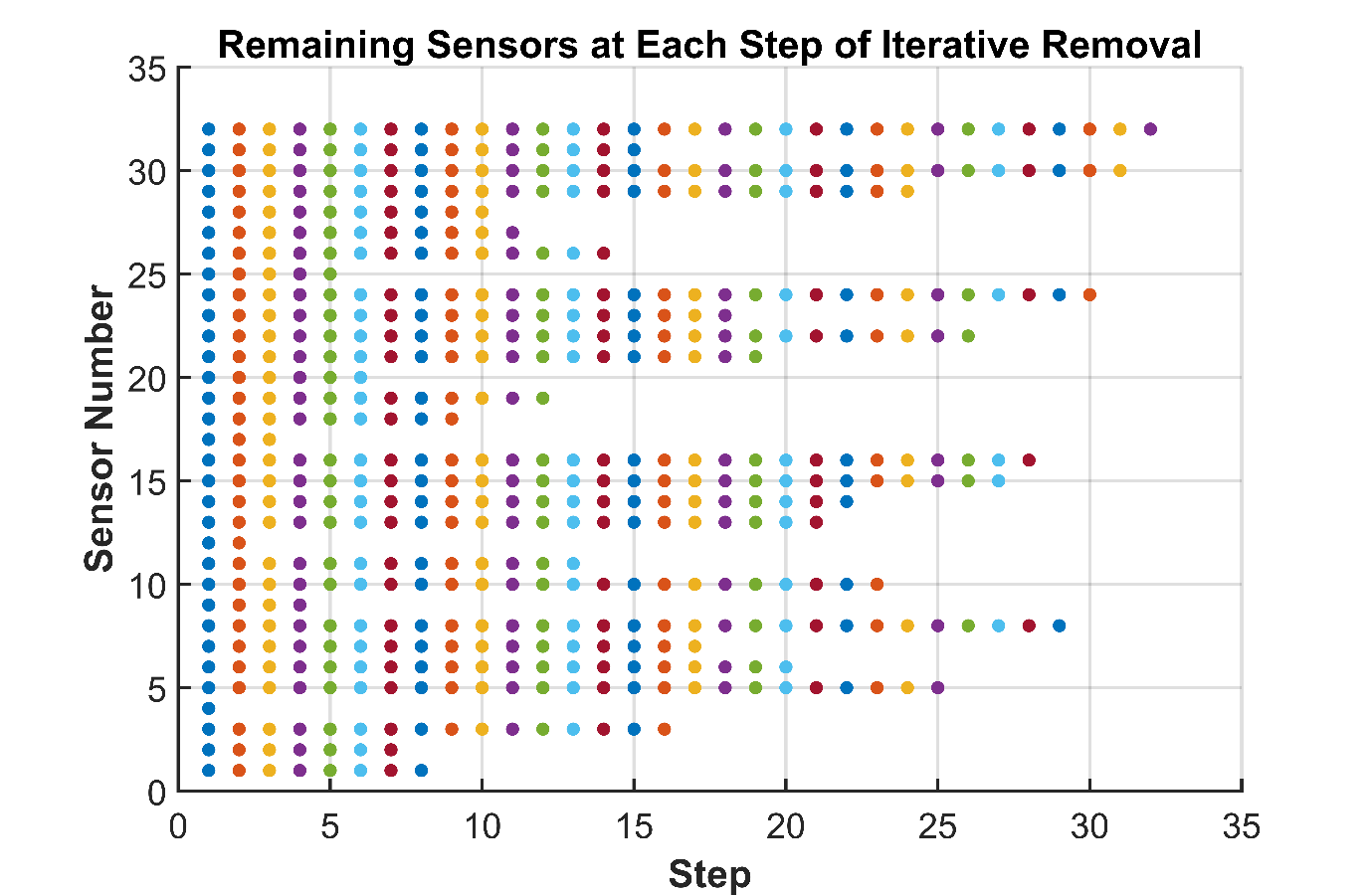


**Figure S43**. Graphical representation of sensor retention across removal steps for PMI: 2 days vs. PMI: 3 days discrimination (Classifier A3). The sensor utility ranking algorithm was applied exclusively to the training dataset to optimize sensor selection.


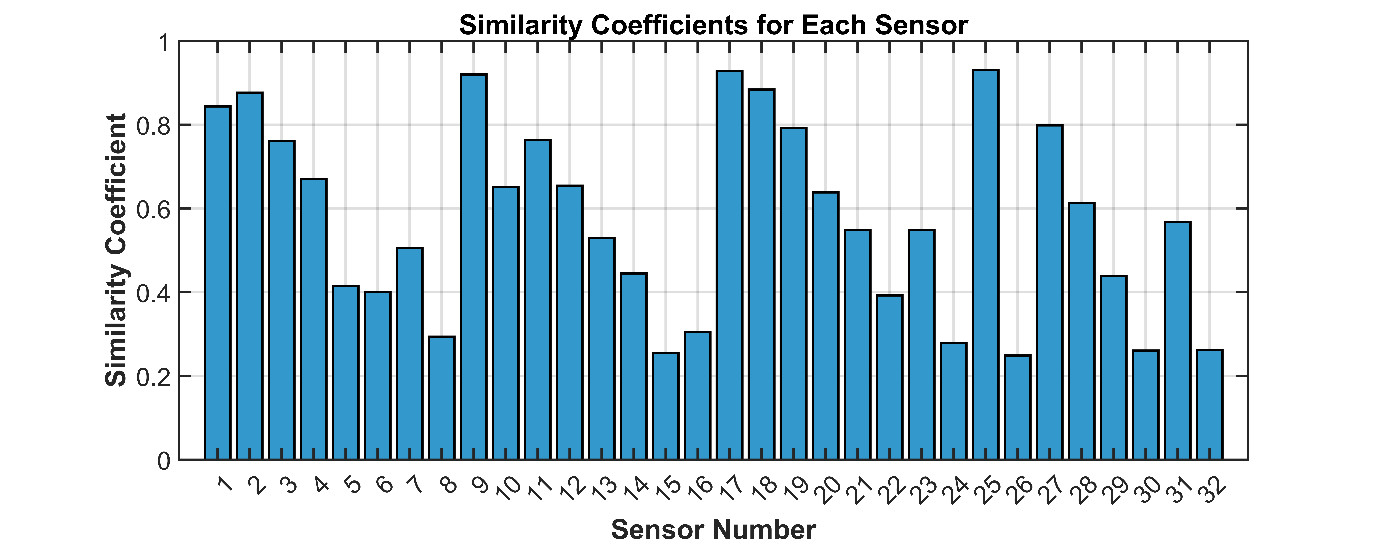


**Figure S44.** Similarity coefficients across the 32-sensor array discriminating animal samples at different PMIs (Classifier A4). Bars denote the mean Pearson correlations between all inter-class signal pairs for each sensor. Lower similarity coefficients indicate greater discriminatory power between classes. The sensor utility ranking algorithm was applied exclusively to the training dataset to optimize sensor selection.


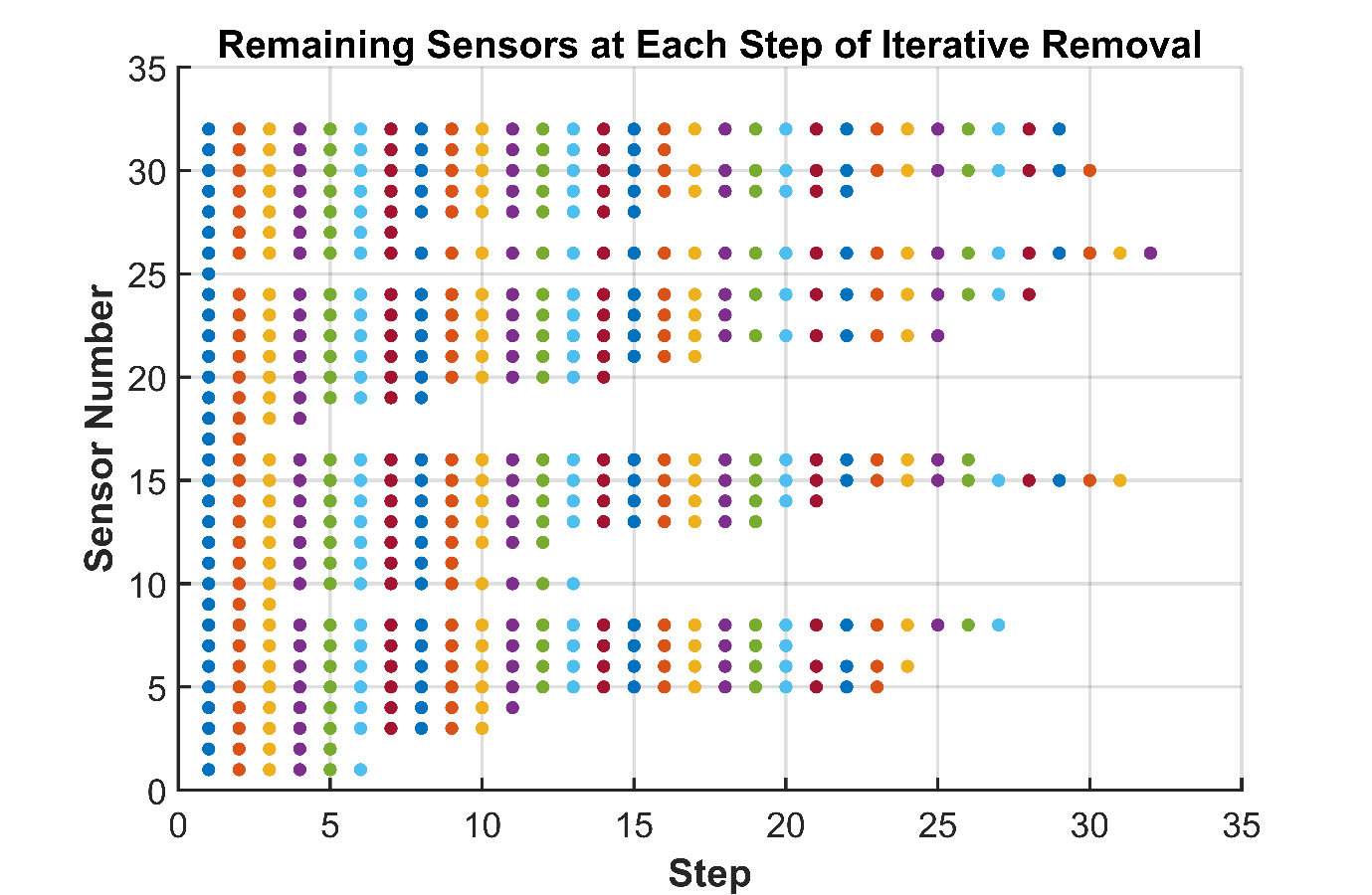


**Figure S45**. Graphical representation of sensor retention across removal steps for PMI: 4-11 day vs. PMI: 8–32 days discrimination (Classifier A4). The sensor utility ranking algorithm was applied exclusively to the training dataset to optimize sensor selection.

**Table S15.** Extracted features.

| **Feature Category** | **No. of Features** | **Features** |
| --- | --- | --- |
| Statistical | 17 | Root Mean Square (RMS), Standard Deviation (Std), Mean, Minimum (Min), Maximum (Max), Shape Factor, Skewness, Kurtosis, Range, Median, Impulse Factor, Crest Factor, Clearance Factor, Shannon Entropy, Harmonic Mean, Mean Absolute Deviation (MAD), Interquartile Range (IQR) |
| Time-Domain | 18 | Area Under Signal, Slope, Zero-Crossing Rate, Activity, Mobility, Complexity, Peak Value, Signal Energy, Time of Minimum Response (t*_min_*), Time of Maximum Response (t*_max_*), Peak-to-Peak Value (PtP), Peak Count, Maximum First Derivative, Maximum Second Derivative, Lag-1 Autocorrelation, Signal-to-Noise Ratio (SNR), Signal-to-Noise and Distortion Ratio (SINAD), Total Harmonic Distortion (THD) |
| Frequency-Domain | 7 | Peak Amplitude, Peak Location, Mean Frequency, Median Frequency, Occupied Bandwidth, Band Power, Power Bandwidth |

**Table S16.** Description of all 85 features extracted from 32 sensor responses per measurement (6000 points, 600 *s*, 10 *Hz* sampling rate). Features 1-32 were computed on both raw and smoothed-normalized signals (64 total). Feature SNR(1) was computed once using the signal residual before and after smoothing. Additional 20 features were extracted using *signalTimeFeatureExtractor* and *signalFrequencyFeatureExtractor*. SNR(1) and SNR(2) differ due to distinct methodologies: customized code for SNR(1), *signalTimeFeatureExtractor* for SNR(2). All features were computed using MATLAB R2024a.

| **Feature** | **Meaning** | **Formula** |
| --- | --- | --- |
| Root Mean Square | Measure of the effective or average power of the signal | $x_{RMS}=\sqrt{\frac{1}{N}\sum_{n=1}^{N} \left\vert x_{n} \right\vert^{2}}$ |
| Standard Deviation | Measure of the dispersion or spread of the signal values around the mean | $Std=\sqrt{\frac{1}{N-1}\sum_{i=1}^{N} \left\vert x_{i}-\mu\right\vert^{2}}$ |
| Mean | Arithmetic average of signal values | $\mu=\frac{1}{N}\sum_{i=1}^{N} x_{i}$ |
| Minimum | Smallest value observed in the signal | *min(x)* function |
| Maximum | Largest value observed in the signal | *max(x)* function |
| Area Under Signal | Cumulative sum of the absolute values of the signal over time | Numerical integration via the trapezoidal method |
| Shape Factor | Ratio between the RMS value and the mean value of the signal | $x_{SF}=\frac{x_{RMS}}{\frac{1}{N}\sum_{i=1}^{N} \left\vert x_{i} \right\vert}$ |
| Slope | Rate of change of the signal over time | Fit a first-degree polynomial to the data |
| Zero-Crossing Rate | Rate at which the signal crosses the zero axis | $ZCR=\frac{1}{2W_{L}}\sum_{n=1}^{W_{L}} \left\vert sgn\left[ x_{i}\left( n \right) \right]-sgn\left[ x_{i}\left( n \right)-1 \right] \right\vert$ |
| Skewness | Measure of the asymmetry of the distribution of signal values | $x_{skew}=\frac{\frac{1}{N}\sum_{i=1}^{N} \left\vert x_{i}-\bar{x} \right\vert^{3}}{\left[ \frac{1}{N}\sum_{i=1}^{N} \left\vert x_{i}-\bar{x} \right\vert^{2} \right]^{3/2}}$ |
| Kurtosis | Measure of the tailedness or peakedness of the distribution of signal values | $x_{kurt}=\frac{\frac{1}{N}\sum_{i=1}^{N} \left\vert x_{i}-\bar{x} \right\vert^{4}}{\left[ \frac{1}{N}\sum_{i=1}^{N} \left\vert x_{i}-\bar{x} \right\vert^{2} \right]^{2}}$ |
| Range | Difference between the max and min values of the signal | $Range=\max\left( x \right)-\min(x)$ |
| Activity | Measure of the overall variance of the signal | $var\left( x \right)=\frac{1}{N-1}\sum_{i=1}^{N} \left\vert x_{i}-\mu\right\vert^{2}$ |
| Mobility | Measure of the rapidity of changes in the signal | $Mobility=\sqrt{\frac{var\left( \dot{x}\left( t \right) \right)}{var\left( x\left( t \right) \right)}}$ |
| Complexity | Measure of the waveform complexity or irregularity of the signal, relative to its mobility | $Complexity\boldsymbol{=}\frac{\sqrt{\frac{var\left( \ddot{x}\left( t \right) \right)}{var\left( \dot{x}\left( t \right) \right)}}}{Mobility}$ |
| Median | Middle value of the array when the values are arranged in ascending order | *median(x)* function |
| Peak Value | Max absolute value of the sensor response, indicating the strongest reaction to VOCs | $x_{P}=\max_{i} \left\vert x_{i} \right\vert$ |
| Impulse Factor | Ratio of the peak absolute value to the mean sensor response, quantifying the peak sharpness | $x_{IF}=\frac{x_{P}}{\frac{1}{N}\sum_{i=1}^{N} \left\vert x_{i} \right\vert}$ |
| Crest Factor | Peak value divided by RMS | $x_{crest}=\frac{x_{P}}{\sqrt{\frac{1}{N}\sum_{i=1}^{N} x_{i}^{2}}}$ |
| Clearence Factor | Peak value divided by the squared mean value of the square roots of the absolute amplitudes | $x_{clear}=\frac{x_{P}}{\left( \frac{1}{N}\sum_{i=1}^{N} \sqrt{\left\vert x_{i} \right\vert} \right)^{2}}$ |
| Signal Energy | Cumulative energy of the sensor response, calculated as the integral of the squared response over time | $E=\int_{t_{1}}^{t_{2}} x^{2}\left( t \right)dt$ |
| Time of Minimum Response | Time at which the min sensor response occurs in the processed signal | $t_{min}=t\left[ \arg\min\left( x \right) \right]$ |
| Time of Maximum Response | Time at which the max sensor response occurs in the processed signal | $t_{max}=t\left[ \arg\max\left( x \right) \right]$ |
| Peak-to-Peak Value | Difference between max and min sensor response, representing the full range of the processed signal | $PtP=\max\left( x \right)-\min\left( x \right)$ |
| Shannon Entropy | Measure of unpredictability or irregularity in the sensor response, quantifying signal complexity | $H=-\sum_{i=1}^{N} p\left( x_{i} \right){log}_{2} p\left( x_{i} \right)$, where *p*(*x*_i_) is the probability of the response value in bin 𝑖, estimated from a histogram of *x* with *N* = 50 bins |
| Harmonic Mean | Reciprocal of the mean of reciprocals of the sensor response, emphasizing smaller values | $m=\frac{n}{\sum_{i=1}^{n} \frac{1}{x_{i}}}$ where *n* is the number of values in *x* |
| Peak Count | Number of local max in the sensor response, indicating the frequency of significant response changes | Number of peaks detected using *findpeaks* on *x* |
| Mean Absolute Deviation | Average absolute deviation of the sensor response from its median, robust to outliers | *median(abs(x – median(x)))* |
| Interquartile Range | Spread of the middle 50% of the sensor response, robust to outliers | *IQR=Q_3_ −Q_1_*, where 𝑄_3​_ and 𝑄_1_​ are the 75^th^ and 25^th^ percentiles of signal |
| Maximum First Derivative | Max absolute value of the first derivative, indicating the steepest rate of change in the response | $max1stDeriv=\max\left\vert\frac{dx}{dt} \right\vert$ |
| Maximum Second Derivative | Max absolute value of the second derivative, indicating the steepest change in the rate of change | $max2ndDeriv=\max\left\vert\frac{d^{2}x}{dt^{2}} \right\vert$ |
| Lag-1 Autocorrelation | Correlation of the sensor response with itself at a lag of one-time step, indicating signal persistence | Calculated using *autocorr* on *x* |
| Signal-to-Noise Ratio SNR(1) | SNR(1) in dB, indicating the quality of the sensor response | $SNR\left( dB \right)=10{log}_{10} \left( \frac{P_{signal}}{P_{noise}} \right)$ |
| Signal-to-Noise and Distortion Ratio | SINAD in dBc of the real-valued sinusoidal signal *x* | Computed using *signalTimeFeatureExtractor* |
| Total Harmonic Distortion | THD in dBc of the real-valued sinusoidal signal *x* | Computed using *signalTimeFeatureExtractor* |
| Signal-to-Noise Ratio SNR(2) | SNR(2) in dB of a signal *x* calculated as the ratio of its summed squared magnitude to that of the noise | Computed using *signalTimeFeatureExtractor* |
| Peak Amplitude (1–6) | Amplitudes of up to 6 significant peaks in the power spectral density (PSD) | Amplitudes of peaks in Welch PSD, computed using *signalFrequencyFeatureExtractor* with *MaxNumExtrema* = 6, *MinSeparation* = 6 bins |
| Peak Location (1–6) | Frequencies of up to 6 significant peaks in the PSD | Frequencies (*Hz*) of peaks in Welch PSD, computed using *signalFrequencyFeatureExtractor* with *MaxNumExtrema* = 6, *MinSeparation* = 6 bins |
| Mean Frequency | Average frequency of PSD, weighted by power | Computed using *signalFrequencyFeatureExtractor* |
| Median Frequency | Frequency at which PSD’s cumulative power reaches 50% of the total power | Computed using *signalFrequencyFeatureExtractor* with Welch PSD |
| Occupied Bandwidth | Bandwidth containing 99% of the signal’s power | Computed using *signalFrequencyFeatureExtractor* with Welch PSD |
| Band Power | Total power in PSD across all frequencies | Computed using *signalFrequencyFeatureExtractor* |
| Power Bandwidth | Bandwidth containing a specified percentage of the peak power (default 10 dB) | Computed using *signalFrequencyFeatureExtractor* with Welch PSD |

**

**

**Figure S46**. Comparison of sensor utility rankings for a 32-sensor e-nose using two methodologies: (1) inter-sensor correlation— sum of absolute correlation coefficients (MATLAB *corrcoeff*) across the array to identify sensors with minimal inter-correlation (Dutta *et al*.^[52]^); and (2) inter-group sensor similarity— mean Pearson correlation coefficients (MATLAB *corr*) of each sensor’s responses between classes (e.g., postmortem vs. antemortem) to identify discriminative sensors (this study).

**
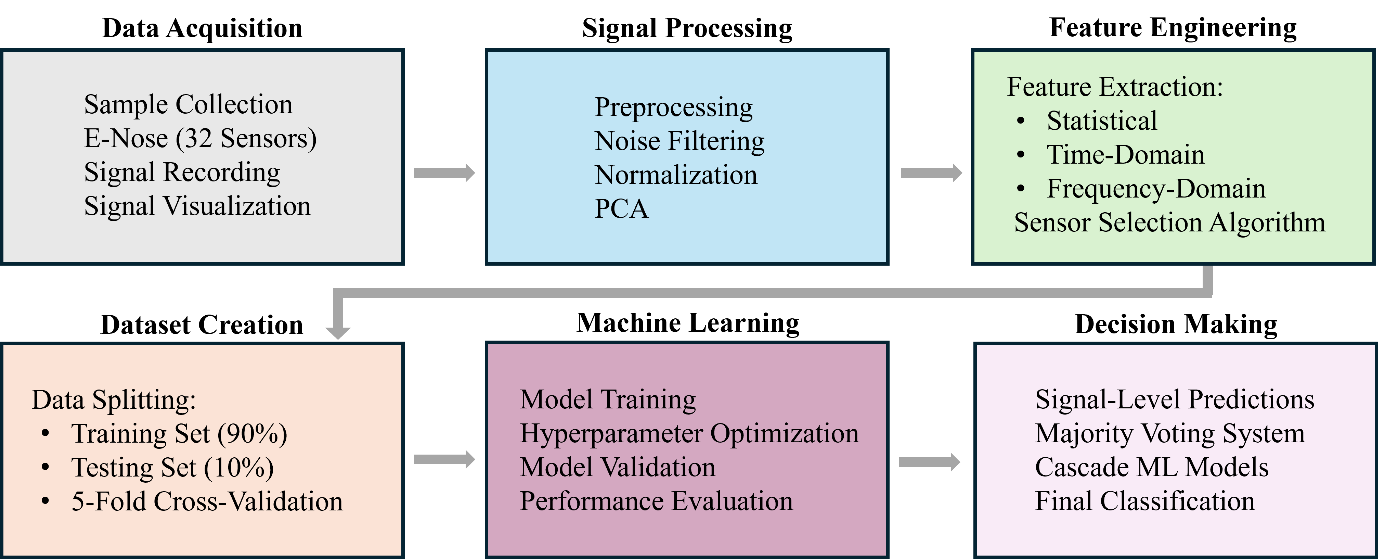
**

**Figure S47.** Machine learning (ML) workflow from data acquisition to decision making.
